# Multiplexed CRISPR/Cas9‐mediated metabolic engineering of γ‐aminobutyric acid levels in *Solanum lycopersicum*

**DOI:** 10.1111/pbi.12781

**Published:** 2017-08-02

**Authors:** Rui Li, Ran Li, Xindi Li, Daqi Fu, Benzhong Zhu, Huiqin Tian, Yunbo Luo, Hongliang Zhu

**Affiliations:** ^1^ The College of Food Science and Nutritional Engineering China Agricultural University Beijing China

**Keywords:** CRISPR/Cas9, genome editing, tomato, GABA, metabolic engineering, multiplex

## Abstract

In recent years, the type II CRISPR system has become a widely used and robust technique to implement site‐directed mutagenesis in a variety of species including model and crop plants. However, few studies manipulated metabolic pathways in plants using the CRISPR system. Here, we introduced the pYLCRISPR/Cas9 system with one or two single‐site guide RNAs to target the tomato phytoene desaturase gene. An obvious albino phenotype was observed in T0 regenerated plants, and more than 61% of the desired target sites were edited. Furthermore, we manipulated the γ‐aminobutyric acid (GABA) shunt in tomatoes using a multiplex pYLCRISPR/Cas9 system that targeted five key genes. Fifty‐three genome‐edited plants were obtained following single plant transformation, and these samples represented single to quadruple mutants. The GABA accumulation in both the leaves and fruits of genomically edited lines was significantly enhanced, and the GABA content in the leaves of quadruple mutants was 19‐fold higher than that in wild‐type plants. Our data demonstrate that the multiplex CRISPR/Cas9 system can be exploited to precisely edit tomato genomic sequences and effectively create multisite knockout mutations, which could shed new light on plant metabolic engineering regulations.

## Introduction

Zinc‐finger nucleases (ZFNs) and transcription activator‐like endonucleases (TALENs) are powerful tools that have recently been used to manipulate genome editing (Chen *et al*., 2014; Ma *et al*., 2015a; Petolino, 2015), but the use of these tools is both laborious and time‐consuming (Doudna and Charpentier, 2014). However, these problems were solved with the discovery of a third‐generation genome‐editing technology derived from the adaptive immune system of *Streptococcus pyogenes*, which is known as clustered regularly interspaced short palindromic repeats (CRISPR)/CRISPR‐associated protein 9 (Cas9) endonuclease system (Wong *et al*., 2015). Single‐guide RNA (sgRNA) and Cas9 endonuclease are the two most important elements in this system. The first 20 nucleotides (nt) at the 5′ end of an sgRNA molecule specifically recognize a three‐base‐pair (bp) protospacer adjacent motif (PAM) sequence that is downstream of the target site. Moreover, the last 80 nt, which belong to a conserved nucleotide sequence, can fold into a particular secondary structure for Cas9 protein binding (Bortesi and Fischer, 2015). With the guidance of the first 20 nt, Cas9 endonuclease can accurately cut the target sequence (Harrison *et al*., 2014). Therefore, genome modification is achieved through standard cellular repair mechanisms, including error‐prone genome repair by nonhomologous end joining (NHEJ) and homology‐directed repair (HDR) (Belhaj *et al*., 2015). In higher plants, NHEJ frequently occurs, causing random insertions or deletions (indels) at target sites, and this results in the loss of gene function through frameshift mutations (Belhaj *et al*., 2015). Because of the simplicity, low cost and high efficiency of the CRISPR/Cas9 system, it has become a powerful tool to study gene function and the improvement of productivity traits in several plant species, including *Arabidopsis thaliana* (Zhang *et al*., 2016), *Glycine max* (Jacobs *et al*., 2015)*, Nicotiana tabacum* (Baltes *et al*., 2014)*, Oryza sativa* (Endo *et al*., 2015)*, Triticum aestivum* (Shan *et al*., 2014)*, Solanum lycopersicum* (Brooks *et al*., 2014) and *Solanum tuberosum* (Wang *et al*., 2015).

**Figure 1:**
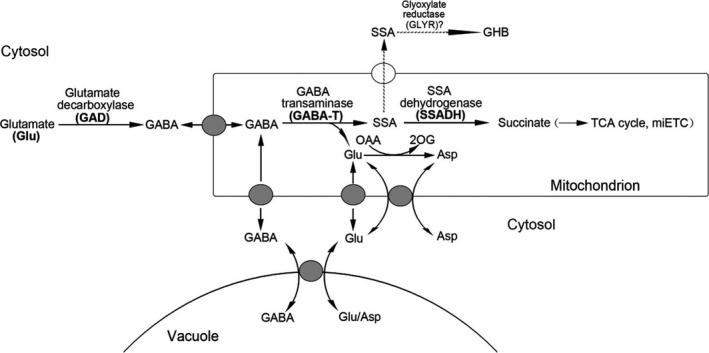
GABA shunt metabolic pathway. GABA is first synthesized from its precursor glutamate via GAD catalysation, and it is then reversibly converted to SSA via GABA‐T catalysation. SSADH further catalyses the oxidation of SSA to succinate, which is an important component in the tricarboxylic acid (TCA) cycle. The mitochondrial transporters shown are GABA permease, a glutamate carrier and an aspartate–glutamate exchanger. SSA, succinic semialdehyde; GHB, γ‐hydroxybutyric acid; OAA, oxaloacetate; 2OG, 2‐oxoglutarate; TCA, tricarboxylic acid; and miETC, mitochondrial electron transport chain.

Tomato is a vitally important food resource for humans (Bergougnoux, 2014). Despite being rich in nutrients such as lycopene, vitamin C, vitamin E and alkaloids, tomatoes produce large amounts of γ‐aminobutyric acid (GABA) during fruit development (Takayama and Ezura, 2015). GABA, a four‐carbon nonprotein amino acid, has recently received considerable attention as a health‐promoting functional compound (Yoshimura *et al*., 2010). In humans, GABA functions as an inhibitory neurotransmitter (Bachtiar *et al*., 2015). People often experience nervousness, depression and insomnia when GABA content greatly decreases (Bachtiar *et al*., 2015; Hall *et al*., 2011). In plants, GABA homeostasis is important for plant growth, and it is regulated by a GABA shunt (Figure 1) (Takayama and Ezura, 2015). In this shunt, GABA is first synthesized from its precursor glutamate via glutamate decarboxylase (GAD) catalysation, and it is then reversibly converted to succinic semialdehyde (SSA) via GABA transaminase (GABA‐T) catalysation. Succinate semialdehyde dehydrogenase (SSADH) further catalyses the oxidation of SSA to succinate, which is an important component in the tricarboxylic acid (TCA) cycle. Koike *et al*. (2013) successfully increased the GABA contents in red ripe fruits by a magnitude of 6.8‐ to 9.2‐fold higher than that in wild‐type (WT) specimens by suppressing *GABA‐T* expression using RNAi. Bao *et al*. (2015) found that silencing *SSADH* led to a 3.6‐fold increase in GABA compared to WT specimens. CAT9 is a transport protein that transports GABA in vacuoles to mitochondria for catabolism and it exhibits increased gene expression levels during the ripening of tomato fruits (Snowden *et al*., 2015).

Plants containing abundant nutrients and metabolites are always consumed by humans or processed by industries (Schaart *et al*., 2016), and several new metabolic engineering technologies have recently been used to increase both the production and quality of plants (Alagoz *et al*., 2016; Schaart *et al*., 2016). For instance, researchers produced opium poppy with high benzylisoquinoline alkaloids using the CRISPR/Cas9 system to manipulate one gene in the metabolic pathway (Alagoz *et al*., 2016). However, metabolic pathways typically contain more than one key gene, and therefore, knocking out a single gene would lead to less accumulation of interesting metabolites. The application of a multiplex CRISPR/Cas9 genome‐editing system to manipulate metabolic engineering in higher plants still requires validation.

In this study, a robust CRISPR/Cas9 system was chosen for its convenience and high‐efficiency genome‐editing capabilities in both monocot and dicot plants (Ma *et al*., 2015b). To test the genome‐editing efficiency of the pYLCRISPR/Cas9 system in tomatoes, we edited the tomato phytoene desaturase gene (*slyPDS*). The severity of the bleached phenotype of most transgenic tomato seedlings suggested that the pYLCRISPR/Cas9 system could efficiently work in tomatoes. To set up a multiplex CRISPR/Cas9 genome‐editing system for metabolic engineering manipulation in tomatoes, we chose the GABA shunt as a metabolic pathway of interest. Five key genes in the GABA shunt were selected, and a pYLCRISPR/Cas9 plasmid with six sgRNA cassettes for editing of these five genes was constructed. Genome editing was applied to 53 plants that covered six different GABA mutants, and the GABA contents were significantly higher in the leaves and fruits of CRISPR mutants than in the WT plants. Specifically, the GABA contents in the leaves of quadruple mutants were greater than 19‐fold higher compared to WT plants. Additionally, GABA overaccumulation significantly affected plant vegetative and reproductive growth. Together, these findings provide new insights into the application of multiplex CRISPR/Cas9 genome editing to plant metabolic engineering and reveal the functional role of GABA in tomato growth.

## Results

### pYLCRISPR/Cas9 system‐mediated mutagenesis of *PDS* in tomatoes

**Figure 2:**
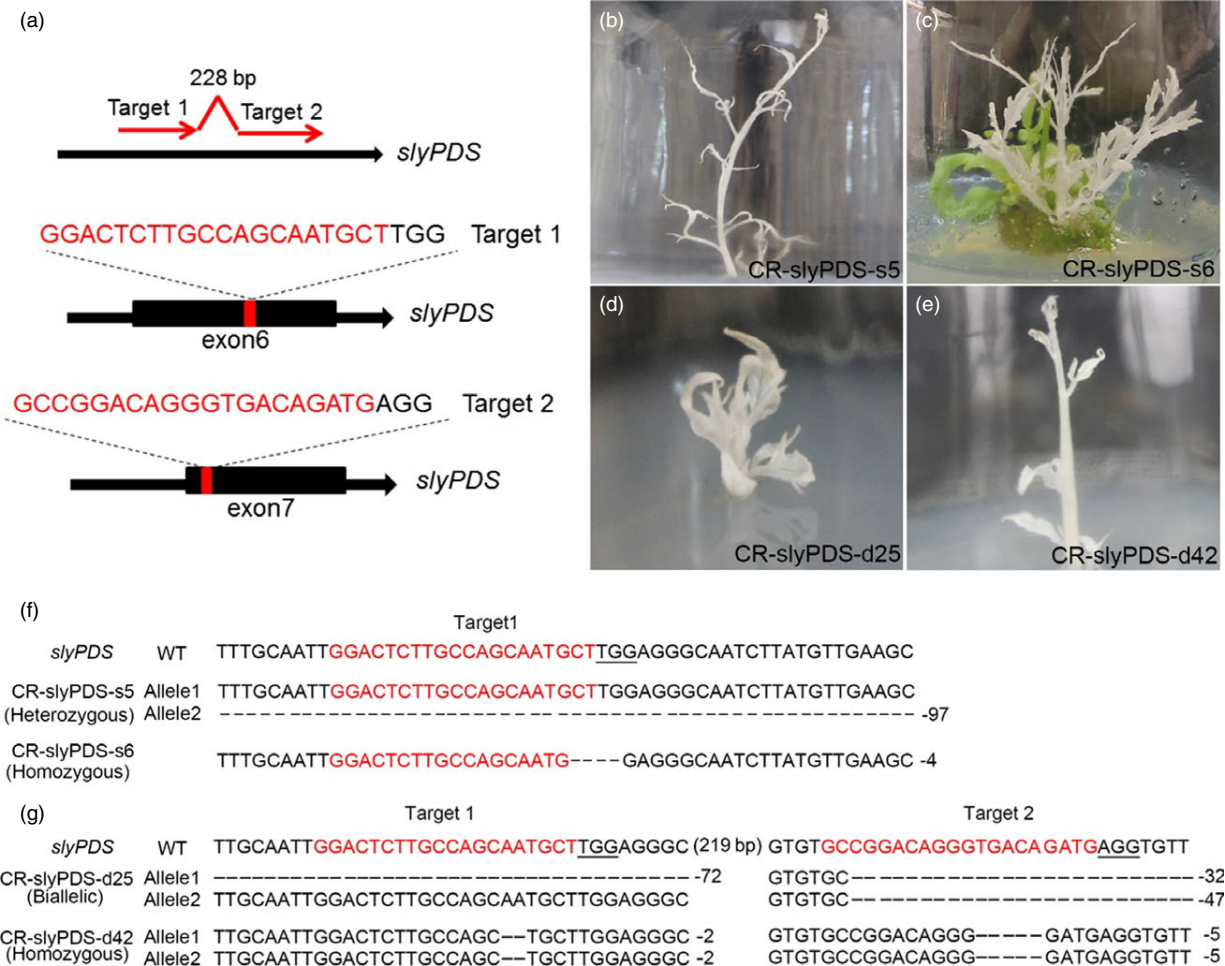
pYLCRISPR/Cas9 system‐mediated genome editing in tomatoes. (a) Schematic illustration of the two target sites in the *slyPDS* genomic sequence. Black boxes indicate exons. Typical albino phenotypes of transgenic AC lines (b and c) and MT lines (d and e) regenerated from the callus. Mutant alleles identified in regenerated AC lines (f) and MT lines (g). Red letters indicate target sites, and minus symbols represent deletions.

Two target sites were designed for *slyPDS* based on the selection (Figure 2a). We selected one or two sgRNAs to target the *slyPDS* gene in tomato cultivars *Solanum lycopersicum* ‘Ailsa Craig’ (AC) and ‘Micro‐Tom’ (MT), respectively (Figures S1 and S2). Most transgenic tomatoes developed a strong photobleached phenotype (Figures 2, S1 and S2). To identify *slyPDS* editing types, 34 independent transgenic AC plants and 38 independent transgenic MT plants were selected for analysis. Heterozygous, biallelic and homozygous *slyPDS* mutations were present in the T0 generation, and the majority (57.1%) was composed of heterozygous lines for AC plants (Figures 2f and S1g). The editing rate of transgenic AC plants was 61.8% (Table S1). Regarding MT plants, the editing rate was 68.4%, including homozygous, heterozygous and biallelic mutations (Table S2, Figures 2g and S2g). Notably, we found two lines of interest (CR‐slyPDS‐d42 and CR‐slyPDS‐d47) that had concurrent homozygous modifications at both sgRNA target sites of sly*PDS* in the T0 generation (Figures 2g and S2g). In addition, we selected four putative off‐targets for each target site and we checked three homozygous mutation tomato plants (CR‐slyPDS‐s6, CR‐slyPDS‐d42 and CR‐slyPDS‐d47). No mutations were found in any putative off‐target sites (Table S3), and these results suggested that the pYLCRISPR/Cas9 system is an efficient genome‐editing tool for tomatoes.

### Selection of target genes in GABA shunt and sgRNA design

**Figure 3:**
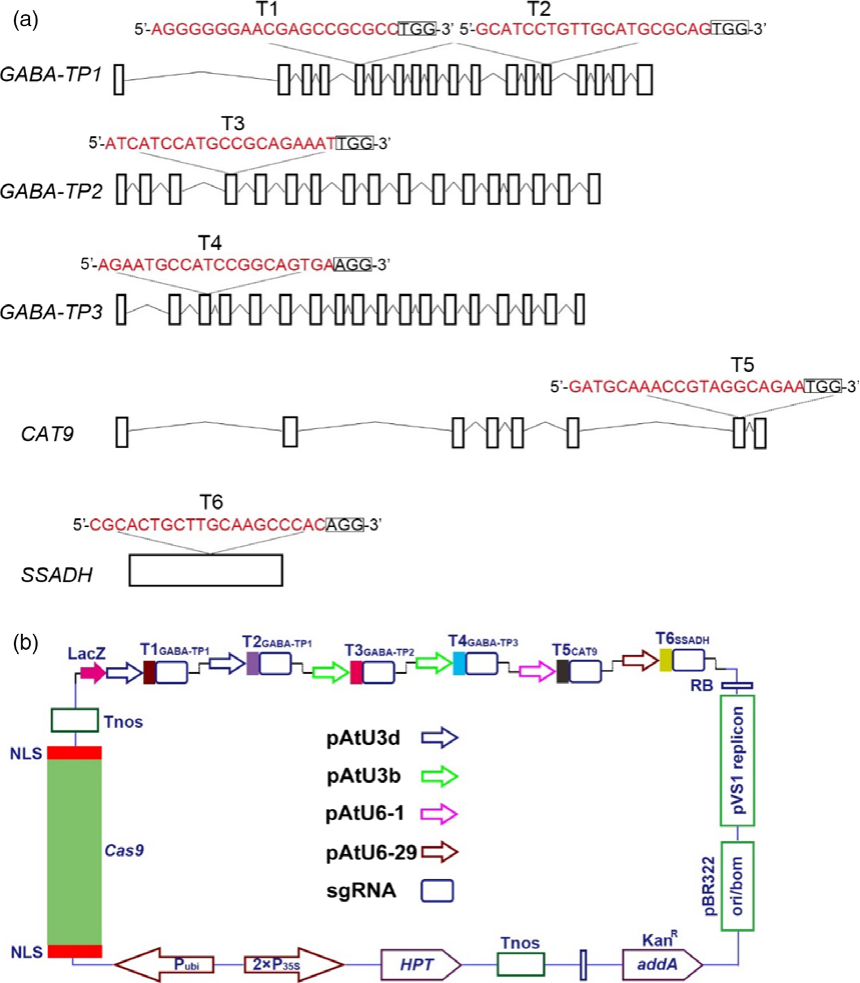
sgRNA design and pYLCRISPR/Cas9‐GABA vector for multigene targeting. (a) Five target genes were selected based on the GABA shunt metabolic pathway, and six target sites were designed. The 20‐bp target sequences were marked in red, and small rectangle frames indicate the PAM. (b) Schematic illustration of the pYLCRISPR/Cas9‐GABA vector for multigene editing.

To develop and apply our highly efficient CRISPR/Cas9 system to metabolic engineering, GABA was chosen as a metabolite of interest, and target genes were selected in the GABA shunt (Figure 1). Previous studies showed that *GABA‐T*,* SSADH* and *CAT9* played critical roles in GABA metabolism (Bao *et al*., 2015; Koike *et al*., 2013; Snowden *et al*., 2015), so the silencing of these genes could result in increased GABA levels. In tomatoes, the GABA‐T enzyme has two isoforms: pyruvate‐dependent GABA‐T (GABA‐TP) and α‐ketoglutarate‐dependent GABA‐T (GABA‐TK). Although Akihiro *et al*. (2008) found a strong correlation between GABA contents and the enzymatic activity of GABA‐TK in tomatoes, only the *GABA‐TP* gene has been identified in tomato plants. Therefore, five genes, including *GABA‐TP1*,* GABA‐TP2*,* GABA‐TP3*,* CAT9* and *SSADH*, which are involved in GABA metabolism, were selected as target genes (Figures 1 and 3a). Regarding these five genes, we designed six sgRNA target sequences that were driven by LacZ‐AtU3d, AtU3d, AtU3b, AtU3b, AtU6‐1 or AtU6‐29 promoters (Figure 3a and b).

### Characterization of targeted editing in six genes of the GABA shunt

**Figure 4:**
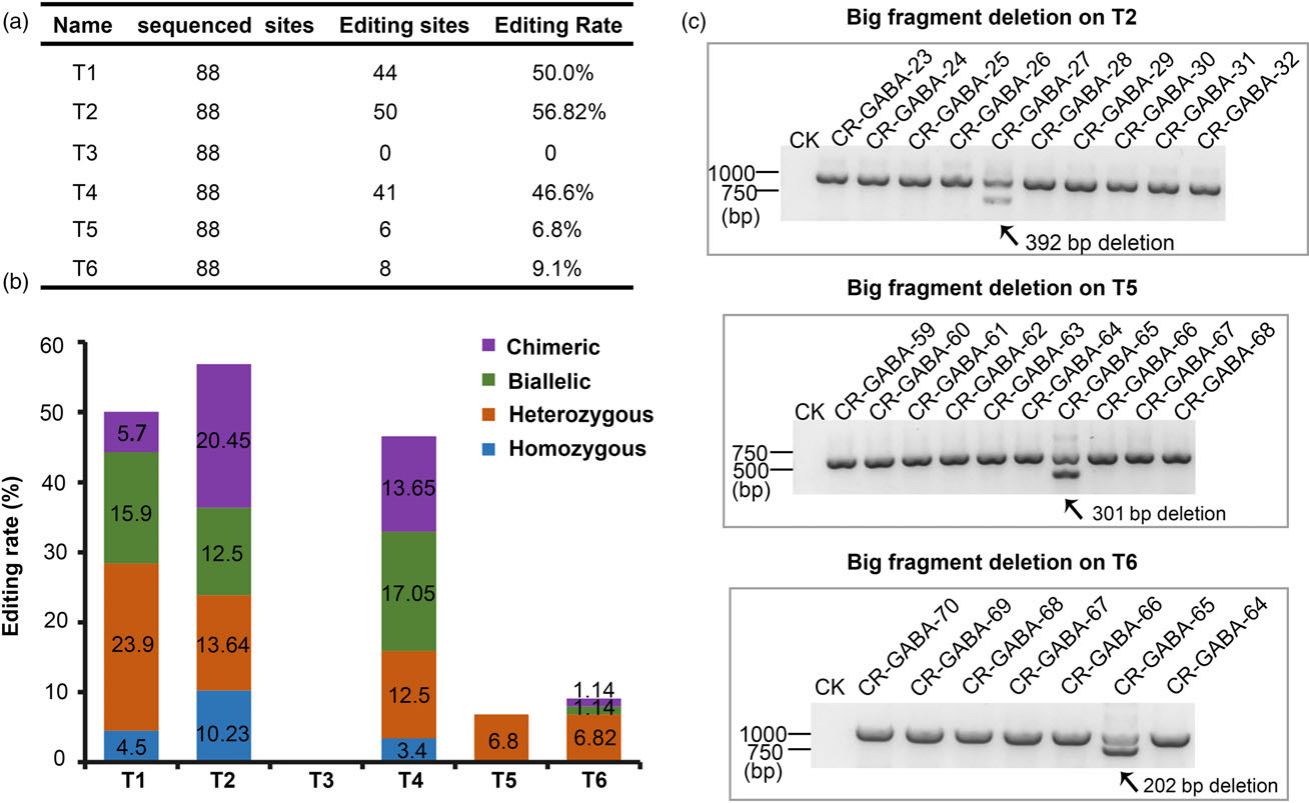
Efficient editing of five target sites in T0 generation plants using the multiplex CRISPR/Cas9 system. (a) Editing rate of the six target sites. (b) Variant genotypes of the six target sites. (c) Editing analysis of T2, T5 and T6 target sites using PCR. The two bands indicate a large deletion in one allele.

Through *Agrobacterium*‐mediated transformation, 88 T0 independent transgenic AC lines were obtained. To analyse the efficiency of our multigene knockout CRISPR/Cas9 system, we analysed the targeted editing of all T0 plants derived from one construct that targeted six sites in five genes. All of the target sites, with the exception of T3, were successfully edited (Figure 4a and b). The editing efficiencies of T1, T2, T3, T4, T5 and T6 were 50.0%, 56.82%, 0.0%, 46.6%, 6.8% and 9.1%, respectively (Figure 4a). We assumed that the failed genome modification of T3 was likely resulted from low GC content (45%). Among the variant editing genotypes of the five target sites, heterozygous mutations were the most common, and homozygous mutations were least abundant (Figures 4b and S3). Moreover, large DNA deletions were found at target site T2, T5 and T6 in our transgenic lines, which would cause target gene dysfunctions (Figure 4c). Moreover, we conducted an off‐target analysis to determine the accuracy of our multigene knockout CRISPR/Cas9 system. Five transgenic plants were randomly chosen to examine off‐target editing and the two most probable off‐target sites were selected for each target site, with the exception of T3. The results indicated that no mutations were found at any of the putative off‐target loci (Table S4), thus demonstrating the accuracy and applicability of this highly efficient multiplex CRISPR/Cas9 system.

### Dramatically enhanced GABA levels detected in the leaves of different CRISPR/Cas9‐mediated GABA mutants

**Figure 5:**
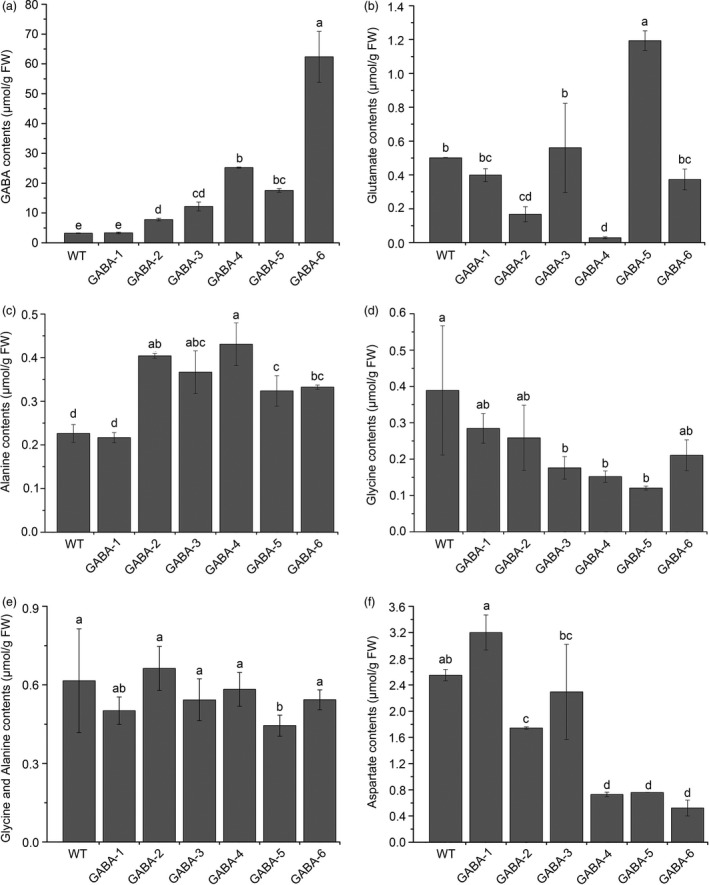
GABA and related amino acid contents in the leaves of WT and variant GABA mutants. Contents of GABA (a), glutamate (b), alanine (c), glycine (d), alanine and glycine (e), and aspartate (f) in the leaves of WT and variant GABA mutants. Error bars representing the standard deviation. Different lower‐case letters indicate statistically significant differences based on an ANOVA followed by Duncan's test (*P *<* *0.05).

Of the 88 transgenic tomato plants, 53 genome‐edited plants were divided into six mutant groups based on different mutated genes, including single, double, triple and quadruple mutants (Table 1 and Figure S3). We renamed these transgenic plant groups GABA‐1 through GABA‐6. We selected a representative transgenic line for each mutant group, and high‐performance liquid chromatography–mass spectrometry (HPLC‐MS) was applied to measure GABA and related amino acid contents in the leaves of these selected lines. Chromatograms of each amino acid are shown in Figure S4. All mutant groups, except GABA‐1, showed significant increases in GABA accumulation in the leaves compared to WT plants (Figure 5a). Among these mutant groups, GABA‐6 (four knocked‐out genes) exhibited the highest GABA levels, which were greater than 19‐fold higher than those observed in WT plants. However, the glutamate contents in the leaves of different GABA mutants varied significantly. For instance, in GABA‐1 and GABA‐3, the glutamate content was comparable to that observed in WT plants. However, glutamate content was significantly lower in GABA‐2, GABA‐4 and GABA‐6 plants, and GABA‐4 exhibited the lowest glutamate content. However, glutamate content in GABA‐5 was higher than that of WT plants (Figure 5b). Although GABA is converted to alanine and glycine by the GABA‐TP reaction, only the glycine content in *GABA‐TP* gene knockout mutants decreased (Figure 5c). Furthermore, all mutant groups, with the exception of GABA‐1, exhibited significantly increased alanine accumulation in the leaves compared to the WT (Figure 5d). Although the contents of these two GABA metabolism‐related amino acids in GABA mutants showed different change tendencies, their total content was comparable to that of WT plants (Figure 5e). Furthermore, we found that all mutants with increased GABA, with the exception of GABA‐3, had significantly lower aspartate content compared to WT plants, thus suggesting a negative correlation between GABA and aspartate (Figure 5f).

**Table 1:** Six GABA mutant types resulting from the CRISPR/Cas9 editing system

Name	Mutants	Edited genes	Numbers
GABA‐1	Single	*GABA‐TP3*	1
GABA‐2	Single	*GABA‐TP1*	12
GABA‐3	Double	*GABA‐TP1, GABA‐TP3*	27
GABA‐4	Triple	*GABA‐TP1, GABA‐TP3, CAT9*	5
GABA‐5	Triple	*GABA‐TP1, GABA‐TP3, SSADH*	7
GABA‐6	Quadruple	*GABA‐TP1, GABA‐TP3, CAT9, SSADH*	1

**Figure 6:**
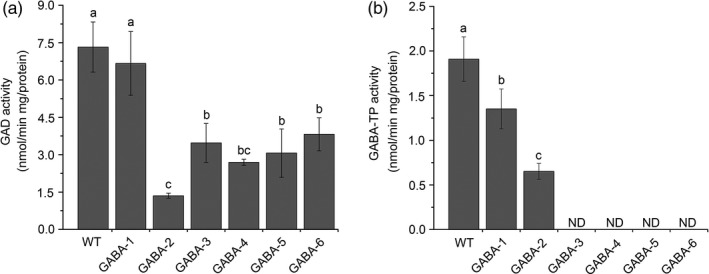
GAD (a) and GABA‐TP (b) enzyme activities in the leaves of WT and variant GABA mutants. Error bars representing standard deviation. Different lower‐case letters indicate statistically significant differences based on an ANOVA followed by Duncan’s test (*P *<* *0.05)

We also determined the GAD and GABA‐TP enzyme activity in GABA mutants and WT plants. The results suggested that although we did not target any GAD genes, GAD enzyme activity decreased as GABA levels increased (Figure 6a), thus implying negative feedback regulation between GAD enzyme activity and GABA content. GABA‐TP activity was extremely low in GABA‐TP gene knockout mutants, especially GABA‐3, GABA‐4 and GABA‐5 mutants in which GABA‐TP enzyme activity was not detected, suggesting a complete knockout of gene functions (Figure 6b). qRT‐PCR analyses of GAD and GABA‐TP transcripts indicated decreased gene expression levels in GABA‐increased mutants, thus illustrating the correlation between enzyme activity and GABA contents (Figure S5). All results showed that our multigene knockout CRISPR/Cas9 system was highly efficient when used to manipulate GABA metabolic pathways in tomatoes.

### Excessive accumulation of GABA inhibited the development of leaf and flower/fruit settings

**Figure 7:**
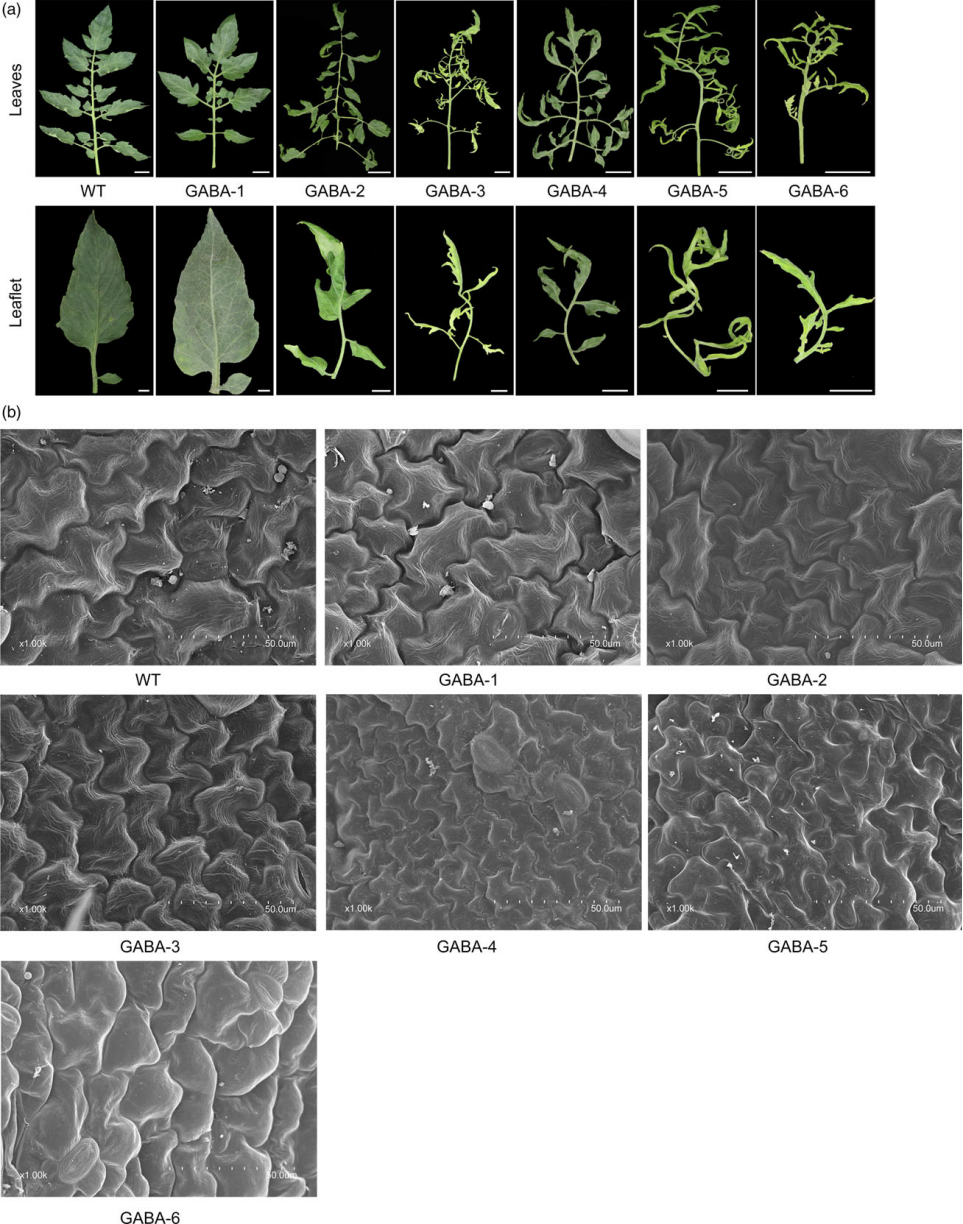
Excessive accumulation of GABA affects compound leaf development. (a) Typical leaf phenotype of WT and GABA mutants. Full leaf: scale bar = 2 cm. Leaflet: scale bar = 1 cm. (b) SEM images of leaflets from WT and GABA mutants. Scale bar = 50 lm. All leaves depicted are leaf no. 6.

As shown in Figure S6, GABA mutants exhibited severe dwarfism at the late stage of growth. The heights of GABA mutants were 95.6% (GABA‐1), 73.2% (GABA‐2), 56.8% (GABA‐3), 48.1% (GABA‐4), 46.7% (GABA‐5) and 32.5% (GABA‐6) lower compared to WT plants. In addition, we found that transgenic lines with significantly increasing GABA concentrations had pale green, curled compound leaves, while transgenic lines that had GABA amounts comparable to those detected in WT plants had normal phenotypes (Figure 7a). Interestingly, we observed that GABA mutants with significant GABA enrichment (e.g. GABA‐5 and GABA‐6) tended to develop a secondary axis and more leaflets (Figure 7a). Scanning electron microscope (SEM) analyses of tomato leaf tissues showed that the cells of GABA mutants were distinct in their size compared to WT and GABA‐1 plants (Figure 7b). The leaf cells of WT and GABA‐1 plants were large and stretched and were small and compressed in GABA mutants with increased GABA concentrations (Figures 5a and 7b). Moreover, we found that tissue necrosis, which could lead to plant death if necrosis accumulation continued, was more likely to appear in the leaves of GABA‐5 and GABA‐6 mutants (Figure S7a). Moreover, DAB (3,3‐diaminobenzidine) staining analyses suggested different H_2_O_2_ accumulation levels in the leaves of GABA mutants compared to WT plants (Figure S7b). Specifically, GABA‐4, GABA‐5 and GABA‐6 plants exhibited severe H_2_O_2_ accumulation compared to WT and other mutant plants, and this accumulation might be responsible for the visible leaf necrosis (Figure S7b). Apart from the necrosis in leaves, we also observed necrosis in the buds of some GABA mutants, which could directly cause severe infertility (Figure S8a). There were fewer flowers in GABA mutants compared to WT plants (Figure S8b). Furthermore, compared to WT plants, the fruit setting ratios in GABA‐3, GABA‐4 and GABA‐5 were quite lower, and several teratogeny‐inactivated fruits were observed in these transgenic plants (Figure S8c).

### Characterization of GABA content and enzymatic activities of GABA shunt enzymes in fruits of GABA mutants

**Figure 8:**
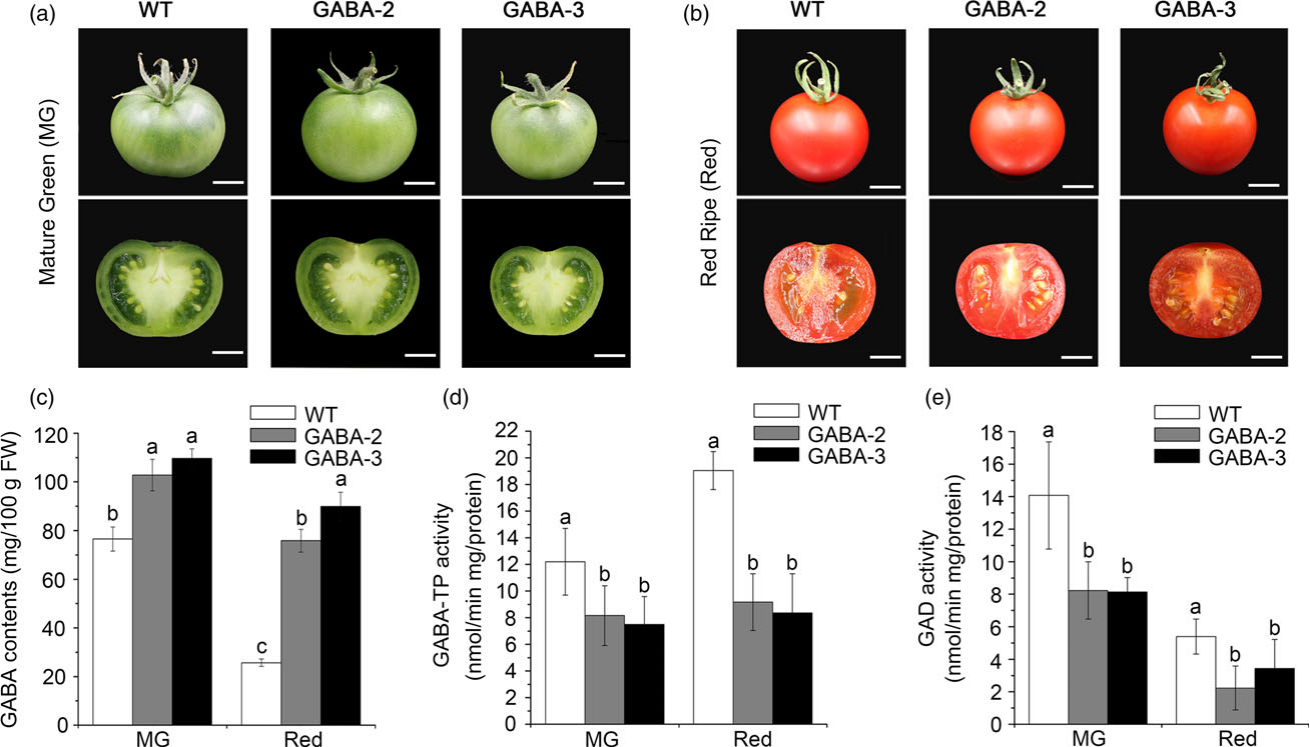
Comparisons of GAD and GABA‐TP enzyme activities and GABA contents in WT, GABA‐2 and GABA‐3 fruits. Photographs of WT, GABA‐2 and GABA‐3 fruits and associated cross sections at MG (a) and Red (b) stages**.** Scale bar = 1 cm. GABA contents (c), GABA‐TP enzyme activity (d) and GAD enzyme activity (e) in WT, GABA‐2 and GABA‐3 fruits. Different lower‐case letters indicate statistically significant differences based on an ANOVA followed by Duncan's test (*P *<* *0.05).

Regarding severe infertility in most GABA mutants, we only obtained tomato fruits from GABA‐2 and GABA‐3 plants (Figure 8a and b). At the mature green (MG) stage, the GABA contents in GABA‐2 and GABA‐3 fruits reached 102.80 mg/100 g FW and 109.72 mg/100 g FW, respectively, equating to 1.34‐ and 1.43‐fold higher than values observed in WT plants, respectively (Figure 8c). In red fruits, the GABA levels of GABA‐2 and GABA‐3 plants were 75.83 mg/100 g FW and 89.88 mg/100 g FW, respectively, equating to 2.95‐ and 3.50‐fold higher compared to WT plants, respectively (Figure 8c). We also determined the GABA‐TP and GAD enzyme activities in the MG and red fruits of GABA‐2 and GABA‐3 mutants. GABA‐TP enzyme activities were detected in both GABA‐2 and GABA‐3 chimeric mutants and were significantly lower at both MG and Red stages compared to WT plants (Figure 8d). Furthermore, compared to significant increases in GABA‐TP enzyme activities in WT fruits from the MG to Red stages, GABA‐TP enzyme activities in both GABA‐2 and GABA‐3 plants remained stable during stage conversion (Figure 8d). Consistent with GAD enzyme activity changes in the leaves of GABA‐increased mutants, GAD enzyme activities in GABA‐2 and GABA‐3 plants were significantly lower at both MG and Red stages (Figure 8e), which suggests that GABA accumulation exhibits negative feedback regulation with regard to its biosynthesis.

## Discussion

### Efficient genome editing in tomatoes using the pYLCRISPR/Cas9 system

The pYLCRISPR/Cas9 multiplex vector system is designed to introduce multiple sgRNA expression cassettes into a single vector (Ma *et al*., 2015b). Here, we used the pYLCRISPR/Cas9 system to successfully edit either one or two specific sites of *slyPDS* gene in tomatoes (Figure 2). The efficiency of genome editing using CRISPR/Cas9 at the target sites was consistent and high enough to elicit a phenotypic response in several genome‐edited lines (Figure 2).

The editing of three or more genomic sites in tomatoes has not been studied yet. In our study, we successfully edited five sites in the tomato genome, and the multisite gene knockout CRISPR/Cas9 system was successfully built and applied, resulting in manipulated GABA metabolic pathways in tomatoes. All target sequences were designed well, but no mutations were observed at T3 in any of the transgenic lines (Figure 4a and b). Therefore, the absence mutations at T3 might be correlated with the lower GC content (45%) of the target site. Intriguingly, the editing efficiency of T5 and T6 was slightly lower compared to other targets, although both T5 and T6 sgRNAs have high GC content (50% and 65%, respectively) and stable secondary structures. We assumed that this discrepancy might have resulted from the different sgRNA expression‐driving capabilities among the promoters (Figure 3b).

Interestingly, we only obtained one large fragment deletion following the editing of a single sgRNA (T2, T5 and T6 sites) even though the efficiency was low (Figures 4c and S3), and this represents a good way to cause dysfunction in a gene. To increase the efficiency of large fragment deletions at target sites, characteristics of the flanking sequence where large fragment deletions occurred are required for future investigations.

The genotypes of CRISPR/Cas9‐generated transgenic lines were mainly determined based on when mutagenesis occurred (Liu *et al*., 2016). For instance, if mutagenesis occurred before the first embryogenic cell divided, the genotype of a diploid plant might be heterozygous, homozygous or biallelic (Liu *et al*., 2016). If mutagenesis occurred after the first embryogenic cell divided and if it occurred in different tissues, the genotype was referred to as chimeric (Liu *et al*., 2016). In our study, most of the genotypes were heterozygous or biallelic (Figure 4b), indicating that our multiplex gene knockout CRISPR/Cas9 system worked early during plant regeneration.

### Multiplex CRISPR/Cas9 system is a powerful tool for plant metabolic engineering

Metabolic engineering is aimed at changing the metabolic composition of the cell, and when applied to plants, food and feed quality improvements are the main goals. Previously, cross or backcross breeding technologies were commonly used to generate multiple mutants for plant metabolic engineering research (Lu *et al*., 2016). However, these simple and conventional methods are relatively time‐consuming. Therefore, metabolic engineering is now more likely to be carried out at the molecular level. Target gene suppression, including RNAi and antisense gene silencing, are commonly used methods that enhance metabolite production, often resulting in the incomplete loss of gene function (Schaart *et al*., 2016). In addition, silencing efficiency gradually decreases during the inheritance process. Most important engineering metabolic pathways in plants often require the concerted regulation of more than one gene, so it is challenging to study metabolic engineering in plants when only a single gene is mutated.

Compared to the aforementioned technologies, the CRISPR/Cas9 system is much more robust and convenient. First, it led to the complete loss of gene function at a high efficiency with approximately no off‐target activities (Pan *et al*., 2016). Second, all induced mutations were at the DNA level, thus rendering them permanent (Cai *et al*., 2015). Third, compared to a simple CRISPR/Cas9 system that targets one gene, the multigene knockout CRISPR/Cas9 system was more efficient. Various mutant groups with different gene knockout combinations could be obtained in a single transformation experiment (Table 1), and this significantly reduced the time needed to obtain desired mutants. Recently, a multiplex CRISPR/Cas9 genome‐editing system was used to obtain a large number of yeast mutant strains with mevalonate levels over 41‐fold greater compared to the WT strain (Jakociunas *et al*., 2015).

In this study, we attempted to set up a multiplexed CRISPR/Cas9 system in tomatoes that would be used for metabolic engineering, and six completely different mutant groups that covered single to quadruple mutants were obtained (Table 1 and Figure S3). The accumulation of GABA in the leaves and fruits of GABA mutant tomatoes was significantly enhanced, with the exception of the GABA‐1 group (Figures 5a and 8c). Comparisons of the GABA content in GABA‐1, GABA‐2 and GABA‐3 indicated *GABA‐TP1* was more important than *GABA‐TP3* in GABA metabolism regulation (Table 1 and Figure 5a), and this result was consistent with previous findings (Koike *et al*., 2013). The comparison of GABA content in GABA‐3, GABA‐4 and GABA‐5 revealed that *CAT9* and *SSADH* were also two important factors in GABA regulation (Table 1 and Figure 5a). The different GABA increases in GABA‐4 and GABA‐5 suggested that compared to *CAT9*,* SSADH* played a more important role in GABA metabolism regulation.

In Koike *et al*. (2013), the glutamate contents were lower in the leaves of GABA‐increased RNAi lines than in the WT. However, the glutamate contents in the leaves of our GABA‐increased mutants varied significantly, and only glutamate in GABA‐2, GABA‐4 and GABA‐6 were lower than that in WT plants. We assumed that glutamate metabolism in plants was complex and regulated by different pathways, but the trends associated with some GABA metabolism‐related amino acids (e.g. alanine, glycine and aspartate) were essentially consistent with the results of a previous study (Koike *et al*., 2013). Furthermore, in Koike *et al*. (2013), the GABA amounts of *GABA‐TP* RNAi lines were 1.3–2.0 times and 6.8–9.2 times higher in mature green and red ripe fruits, respectively, than the contents of WT fruits. In our study, the GABA contents in GABA‐2 and GABA‐3 fruits were 1.34‐ to 1.43‐fold and 2.95‐ to 3.50‐fold higher in green and red fruits, respectively, than the contents in WT fruits. This relatively lower increase in GABA levels might have resulted from the genotypes of GABA mutants. Both GABA‐2 (CR‐GABA‐20) and GABA‐3 (CR‐GABA‐56) mutants were chimeric (Figure S3), and chimeric mutations might not result in the complete loss of gene function, and the GABA‐TP enzyme activity in the fruits of these two GABA mutants remained although the level was significantly lower than that of WT plants (Figure 8). Li *et al*. (2017) used CRISPR/Cas9 techniques that targeted the committed diterpene synthase gene (*SmCPS1*) involved in tanshinone biosynthesis in *Salvia miltiorrhiza*, and different tanshinone accumulation levels were detected in chimeric and homozygous mutants. Therefore, these results suggested that *SmCPS1* in chimeric mutants was knocked down instead of being knocked‐out. Although it would be better to use homozygous mutants to illustrate our results, the edited genes and reduced enzyme activities in fruits of chimeric mutants could also facilitate the increase in GABA and thus support the conclusion of our research. Currently, we are trying to produce homozygous mutants in T1 generation, which we hope could be used to investigate the functional role of GABA in plants.

### GABA affects plant vegetative and reproductive growth

In plants, GABA is not just a metabolite but rather a signal molecule, which was shown to be involved in various physiological processes such as pollen tube growth (Yu *et al*., 2014), regulation of intracellular Ca^2+^ levels (Fait *et al*., 2008) and ethylene production (Shi *et al*., 2010; Takayama *et al*., 2017); however, its functional mechanisms remain largely elusive (Bown and Shelp, 2016; Ramesh *et al*., 2015). In our study, the vegetative growth and flower/fruit setting were severely affected in GABA‐increased mutants (Figure 6 and Figures S6, S7 and S8), and a large number of GABA mutants exhibited dwarfism compared to WT plants (Figure S6). Similar phenotypes were also observed when *GABA‐TP1* was silenced in tomatoes (Koike *et al*., 2013). Studies have shown that GABA controls plant cell elongation in vegetative tissues by regulating the expression of genes encoding secreted and cell elongation‐associated proteins (Renault *et al*., 2011). Therefore, we suppose that in our study, genes related to cell elongation in these tissues were affected by GABA overaccumulation.

Furthermore, the leaf development and morphology of GABA‐increased mutants differed greatly from plants with normal GABA levels. Mutants with significantly high GABA levels had pale green, curled compound leaves, while GABA‐1 mutants and WT plants had normal leaves (Figures 5a and 7). The cells of leaf tissues that exhibited abnormal phenotypes were smaller and more compressed than normal ones (Figure 6b), and these results were consistent with previous study (Akama and Takaiwa, 2007). The leaves of rice and tobacco plants with extremely high GABA levels were also curled and smaller than WT plants (Akama and Takaiwa, 2007). Our results suggested that GABA is involved in the regulation of leaf development. As a by‐product of normal oxygen metabolism, the accumulation of reactive oxygen species (ROS) could lead to damages in plant tissue (Bao *et al*., 2015). Tissue necrosis has been observed in *SSADH*‐deficient *Arabidopsis* mutants (Bouche *et al*., 2003), and in our study, tissue necrosis was also observed in the leaves of *SSADH* knockout lines (GABA‐5 and GABA‐6) (Figure S7a). H_2_O_2_ levels sharply increased when either *SSADH* or *CAT9* were knocked out (GABA‐4, GABA‐5 and GABA‐6) (Figure S7b). Our results were consistent with a previous study in which the silencing of *SSADH* led to GHB accumulation (Figure 1) and the induction of high H_2_O_2_ levels (Ludewig *et al*., 2008).

As the accumulation of large amounts of GABA resulted in abnormal growth, fewer flowers and impaired fertilization (Renault *et al*., 2011), only fruits from GABA‐2 and GABA‐3 mutants were obtained in our study. To exclude the reasons that increased GABA levels in fruits of GABA‐2 and GABA‐3 mutants were induced by small or slow‐growing fruits, we compared the fruit size, colour, shape and ripening behaviour of GABA‐2 and GABA‐3 mutants with those of WT plants, and the results showed that there were no distinct differences among fruits from GABA‐2, GABA‐3 mutants and WT plants at MG and Red stages (Figure 8a and b). Moreover, the edited genes (Figure S3) and reduced enzyme activities (Figure 8d) in GABA‐2 and GABA‐3 mutants further suggested that the increased GABA content in fruits of GABA‐2 and GABA‐3 mutants was induced by loss of gene function, not by small or slow‐growing fruits. Previous study indicated that removal of the autoinhibitory domain of *SlGAD3* could lead to the hyperaccumulation of GABA in tomato fruits as well as orange‐ripe phenotype (Takayama *et al*., 2017). Notably, the GABA levels were 11‐ to 12‐fold higher in *SlGAD3▵*C^OX^ transgenic lines than in WT plants at the BR+10 stage, and these levels were much higher than the GABA levels in our mutants, thus implying that tomato fruit ripening was regulated by GABA in a dosage‐dependent manner.

## Experimental procedures

### Plant materials and growth conditions

*Solanum lycopersicum* cv. Ailsa Craig (AC), *Solanum lycopersicum* cv. Micro‐Tom (MT) and CRISPR/Cas9‐edited transgenic tomato plants were grown in the greenhouse under standard greenhouse conditions (26 °C under 16 h of lighting, followed by 8 h of darkness at 20 °C). Leaf samples were collected 12 weeks after transplantation. Fruits were sampled at 38–42 days postanthesis (DPA) and 48‐52 DPA to obtain MG and Red stages, respectively. Samples were stored at −80 °C. In this study, only T0 generation plants were analysed because *SlGABA‐T* suppression caused severe infertility in transgenic plants.

### Selection of sgRNA target sequence and pYLCRISPR/Cas9‐slyPDS and ‐GABA vector construction

CRISPR‐P (http://cbi.hzau.edu.cn/crispr/) was used to select specific sgRNAs that targeted *SlyPDS*,* SlyGABA‐TP1*,* SlyGABA‐TP2*,* SlyGABA‐TP3*,* SlyCAT9* and *SlySSADH* (Table S5). To obtain high editing efficiency, the GC content at the target site should be greater than 40%, and four or more consecutive T nucleotides in the target sequence should be avoided as the sequence would be recognized as a transcriptional termination signal by RNA polymerase III. Furthermore, RNA Folding program (http://mfold.rna.albany.edu/?q=mfold/RNA‐ Folding‐Form2.3) was used to ensure there were no more than five base pairings between the target sequence and the sgRNA sequence, because the secondary structure of sgRNA greatly affects editing efficiency. To construct the pYLCRISPR/Cas9‐slyPDS and ‐GABA vector, each target sequence was first ligated to its corresponding sgRNA expression cassette during the first PCR. This was followed by a second PCR used to amplify the fragments and to induce *Bsa*I restriction sites in the target, which were then assembled in the pYLCRISPR/Cas9Pubi‐H during one round of cloning via the Golden Gate ligation method (Ma *et al*., 2015b). The oligonucleotide primers used are listed in Table S6.

### Plant transformation

Using the *Agrobacterium*‐mediated transformation method (Van Eck and Kirk, 2006), pYLCRISPR/Cas9‐slyPDS‐expressing plasmids were transformed into both AC and MT, while the pYLCRISPR/Cas9‐GABA plasmid was transformed into AC. The transgenic tomato lines were selected based on hygromycin resistance.

### DNA extraction and mutation detection

Fresh frozen leaves (1–5 mg) were used for DNA extraction by hi‐DNA secure plant kit (Tiangen, Beijing, China). The extracted genomic DNA was then used as a template to amplify the desired fragments in each of the target genes using primers flanking the target sites. The following standard PCR program was used: 94 °C for 3 min; 35 cycles of 94 °C for 30 s, 55 °C for 30 s, and 72 °C for 30 s; and 72 °C for 7 min. PCR products were directly sequenced or cloned into the pEasy‐T1 (TransGen Biotech, China) vector and then sequenced using the Sanger method to identify mutations. Superimposed sequence chromatograms produced by biallelic and heterozygous mutations were decoded by DSDecode (http://dsdecode.scgene.com/) and manual analyses. The oligonucleotide primers used are listed in Tables S7 and S8.

### RNA extraction and reverse transcription

Total RNA was isolated from leaf samples using DeTRNa reagent (EarthOx, CA). The RNA concentration and purity were measured with a NAS‐99 spectrophotometer (ATCGene, NJ). Genomic DNA was removed from extracted total RNA samples by DNase treatment, and the RNA integrity was assessed by agarose gel electrophoresis. Total RNA aliquots of 1 μg were used for cDNA synthesis using the TranScript One‐Step gDNA Removal and cDNA Synthesis SuperMix kit (Trans, Beijing, China) with random primers.

### Quantitative reverse transcription–PCR (qRT‐PCR)

Quantitative reverse transcription–PCR (qRT‐PCR) was performed using SYBR Green PCR Master Mix with a real‐time PCR System CFX96 (Bio‐Rad, CA). The following PCR program was used: 95 °C for 10 min, followed by 40 cycles of 95 °C for 15 s and 60 °C for 30 s. Fluorescence changes were monitored in each cycle, and *Actin* was used as the internal control. The 2^−▵▵Ct^ analysis method was used to calculate the relative expression levels of each sample, and three biological replicates were performed. The oligonucleotide primers used are listed in Table S9.

### Scanning electron microscope observations

For scanning electron microscope (SEM) analyses, leaf samples were first obtained by hand‐cutting with a razor blade. Samples were then immediately fixed in 2.5% glutaraldehyde buffer with 0.1 mol/L sodium phosphate (pH 7.2) for 2 h at room temperature. The samples were subsequently dehydrated three times (for 15 min) using the following ethanol series: 25%, 50%, 75%, 95% and 100%. After being dried with a critical point dryer, the samples were placed on metal and examined with a SEM (HITACHI S‐3400N, Tokyo, Japan).

### Detection of H_2_O_2_

H_2_O_2_ was detected using the 3,3′‐diaminobenzidine (DAB) uptake method. A fresh leaflet was cut and soaked in 1 mg/mL of DAB‐HCl (pH 3.8) for 8 h in the dark, and it was then cleared by boiling in 95% ethanol for 15 min.

### Extraction and measurement of GABA and associated amino acid contents in leaves

Frozen leaf tissues were first dehydrated using a vacuum freeze drier for 2 days to let the sample completely dry. A 1 mL volume of water was then added to 20 mg of powder, which was treated with an ultrasonic wave for 30 min before being centrifuged for 10 min at 15 000 ***g***. Derivatization of GABA was performed using an AccQTag™ Ultra Derivatization Kit (Waters, Milford), and the derived samples were analysed using HPLC‐MS (Thermo Fisher Scientific, Waltham, MD). An HPLC Column BEH C18 (2.1 × 100 mm, 1.7 μm) was used to separate 1‐μL extracts. Chromatographic separation was performed over a 15.5‐min analysis time using an organic mobile phase (ACN:H_2_O = 80:20) as solvent B and an aqueous mobile phase (20 mM NH_4_Ac) as solvent A, following a linear gradient. The elution program is listed in Table S10. The flow rate of the gradient mobile phase was 0.3 mL/min, and the column temperature was adjusted to 40 °C. The mass spectrometry conditions were as follows: positive ion mode, sheath gas flow rate 30 L/h, aux gas flow rate 10 L/h, sweep gas flow rate 5 L/h, spray voltage 3.5 kV, capillary temperature 320 °C, gas heater temperature 350 °C and s‐lens RF level 55%. Three biological replicates were used for each WT and CR‐GABA transgenic line. A standard solution of an amino acid mixture was used in HPLC‐MS analyses, and sample amino acid contents were quantified based on standard curves.

### Extraction and measurement of GABA contents in fruits

Regarding fruits, GABA extraction and measurement were performed according to GABase protocol (Koike *et al*., 2013). First, 50–70 mg of frozen tomato fruit powder and 500 μL of 8% TCA solution were vortexed for 30 s, and 300 μL of supernatant was then added to 400 μL of diethyl ether after centrifugation at 16 060 ***g*** for 20 min at 4 °C. After vortexing for 10 min and centrifugation at 10 000 ***g*** for 10 min, the upper phase was removed, and the lower phase was again mixed vigorously with diethyl ether for 10 min. After centrifugation at 10 000 ***g*** for 10 min at 4 °C, the upper phase was removed, and the tubes were dried for 1 h to completely remove diethyl ether. ‘GABase’ assay for GABA measurement was performed using the method described by Jakoby (1962) with slight modification. In the ‘GABase’ assay, the reduction in NADP to NADPH was spectrophotometrically monitored as a function of time at 340 nm, 37 °C, and a pH level of 8.6 using GABA as a substrate. Three biological replicates were used for each WT and GABA mutant.

### GAD and GABA‐TP enzyme activity measurement

Protein extraction and enzyme assay were performed according to Renault *et al*. (2010) with slight modifications. Regarding the GAD assay, protein extractions were performed in an extraction buffer containing 100 mm Tris‐HCl (pH 7.5), 1 mm EDTA, 10% (v/v) glycerol and a 1% (v/v) protease inhibitor cocktail. For leaf and fruit samples, four and two volumes of extraction buffer (v/w) were, respectively, added before homogenization. The supernatant was used for the enzyme assay and for protein quantification after centrifugation at 20 000 ***g*** for 20 min at 4 °C. For leaves and fruits, ~30 and ~20 μg of protein were, respectively, added to a reaction buffer containing 150 mm potassium phosphate (pH 5.8), 0.1 mm pyridoxal‐5‐phosphate (PLP) and 20 mm L‐glutamate in a final volume of 150 μL. Control assays were concurrently performed by replacing native enzyme extracts with boiled enzyme extracts in the assay. After incubation at 30 °C for 60 min, samples were heated at 97 °C for 7 min to stop the reaction. GAD enzyme activity was calculated by quantifying the amount of produced GABA through GABase assay described above; 20 μL of GAD enzyme assay was used to perform the GABase assay. For each sample, an increase in OD_340 nm_ was recorded, and its corresponding control was subtracted. The amount of GABA was calculated based on an externally calibrated GABA curve.

Regarding the GABA‐TP assay, protein was extracted using an extraction buffer containing 100 mm Tris‐HCl (pH 8.0), 5 mm EDTA, 1.5 mm dithiothreitol (DTT), 10% (v/v) glycerol and 1% (v/v) protease inhibitor cocktail. The enzyme assay for leaf and fruit samples was performed with ~30 and ~20 μg of protein, respectively, in a reaction buffer containing 50 mm Tris‐HCl (pH 8.0), 1.5 mm DTT, 0.1 mm PLP, 0.75 mm EDTA, 10% (v/v) glycerol, 16 mm GABA and 4 mm pyruvate in a final volume of 150 μL. Control assays were performed as described above, and enzyme reactions were incubated and stopped as previously described in the GAD assay. Enzyme activity was calculated by quantifying the amount of L‐alanine produced during the alanine dehydrogenase (AlaDH) assay. The AlaDH assay was performed with 40 μL of the GABA‐TP assay product in an mix containing 50 mm sodium carbonate buffer (pH 10.0), 1 mm β‐NAD^+^ and 0.02 units of *Bacillus cereus* AlaDH in a final volume of 200 μL. For each sample, a duplicate determination of L‐alanine content was conducted. The increase in OD_340 nm_ for each sample was recorded, and its corresponding control was subtracted. The amount of L‐alanine was calculated based on an externally calibrated L‐alanine curve.

Protein concentrations were determined by the Bradford method (Bradford, 1976).

## Funding

This work was supported by grants from Chinese Universities Scientific Fund (2017QC132) and Great Northern Agriculture Education Fund (1061‐2415003) and National Natural Science Foundation of China (91540118 and 31622050) to H.Z. No conflict of interest has been declared.

## Author Contributions

R.L., R.L. and H.Z. designed experiments. R.L. and R.L. performed most of the experiments. X.L. carried out genome editing on tomato MT lines. D.F., B.Z. and H.T. provided materials and intellectual input for the work. R.L., R.L., Y.L. and H.Z. wrote the manuscript.

## Supporting information


**Figure S1** Genome editing of slyPDS in tomato AC plants.
**Figure S2** Genome editing of slyPDS in tomato MT plants.
**Figure S3** Genome editing type of 53 CR‐GABA mutants.
**Figure S4** HPLC‐MS Chromatogram of GABA and related amino acid.
**Figure S5** qRT‐PCR analysis of GABA shunt related genes in the leaves of WT and GABA mutants.
**Figure S6** Excessive GABA drafted tomato plants.
**Figure S7** Tissue necrosis of GABA mutants.
**Figure S8** Over accumulation of GABA causes smaller fruits due to impaired fertilization.
**Table S1** Determination of PDS mutations that occurred in T0 transgenic AC plants.
**Table S2** Determination of PDS mutations that occurred in T0 transgenic MT plants.
**Table S3** Off‐target detection in three CR‐slyPDS mutants.
**Table S4** Detection of mutations on off‐target sites in CR‐GABA mutants.
**Table S5** Sequence of target sites.
**Table S6** Primers used for recombinant pYLCRISPR/Cas9 vector construction.
**Table S7** Primers used for target site mutation analysis.
**Table S8** Primers used for off‐target site mutation analysis.
**Table S9** Primers used for qRT‐PCR.
**Table S10** Elution program of HPLC‐MS.
