# Supplementary material for: Multiplexed CRISPR/Cas9‐mediated metabolic engineering of γ‐aminobutyric acid levels in Solanum lycopersicum
Source: Plant Biotechnol J. 2017 Aug 2;16(2):415–27. doi: 10.1111/pbi.12781 (PMC5787826; doi:10.1111/pbi.12781)
Supplement: Supplementary file 1 — Figure S1 Genome editing of slyPDS in tomato AC plants. Figure S2 Genome editing of slyPDS in tomato MT plants. Figure S3 Genome editing type of 53 CR‐GABA mutants. Figure S4 HPLC‐MS Chromatogram of GABA and related amino acid. Figure S5 qRT‐PCR analysis of GABA shunt related genes in the leaves of WT and GABA mutants. Figure S6 Excessive GABA drafted tomato plants. Figure S7 Tissue necrosis of GABA mutants. Figure S8 Over accumulation of GABA causes smaller fruits due to impaired fertilization. Table S1 Determination of PDS mutations that occurred in T0 transgenic AC plants. Table S2 Determination of PDS mutations that occurred in T0 transgenic MT plants. Table S3 Off‐target detection in three CR‐slyPDS mutants. Table S4 Detection of mutations on off‐target sites in CR‐GABA mutants. Table S5 Sequence of target sites. Table S6 Primers used for recombinant pYLCRISPR/Cas9 vector construction. Table S7 Primers used for target site mutation analysis. Table S8 Primers used for off‐target site mutation analysis. Table S9 Primers used for qRT‐PCR. Table S10 Elution program of HPLC‐MS. [file PBI-16-415-s001.docx]

**Supplemental Information**

**Figure S1.** Genome editing of *slyPDS* in tomato AC plants.

**Figure S2.** Genome editing of *slyPDS* in tomato MT plants.

**Figure S3.** Genome editing type of 53 CR-GABA mutants.

**Figure S4.** HPLC-MS Chromatogram of GABA and related amino acid.

**Figure S5.** qRT-PCR analysis of GABA shunt related genes in the leaves of WT and GABA mutants.

**Figure S6.** Excessive GABA drafted tomato plants.

**Figure S7.** Tissue necrosis of GABA mutants.

**Figure S8.** Over accumulation of GABA causes smaller fruits due to impaired fertilization.

**Table S1** Determination of *PDS* mutations that occurred in T0 transgenic AC plants.

**Table S2** Determination of *PDS* mutations that occurred in T0 transgenic MT plants.

**Table S3** Off-target detection in three CR-slyPDS mutants.

**Table S4** Detection of mutations on off-target sites in CR-GABA mutants.

**Table S5** Sequence of target sites.

**Table S6** Primers used for recombinant pYLCRISPR/Cas9 vector construction.

**Table S7** Primers used for target site mutation analysis.

**Table S8** Primers used for off-target site mutation analysis.

**Table S9** Primers used for qRT-PCR.

**Table S10** Elution program of HPLC-MS.

**
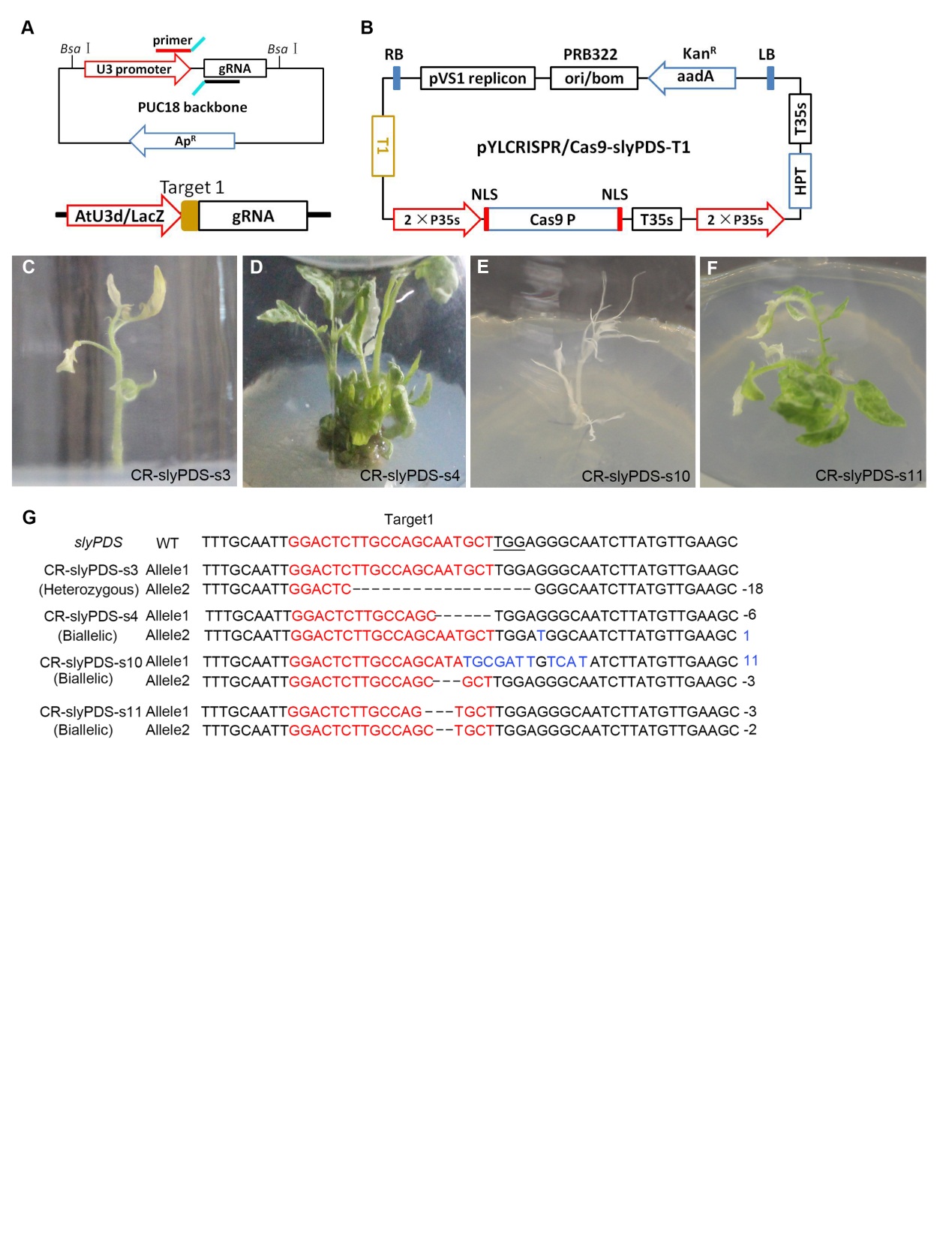
**

**Figure S1. Genome editing of *slyPDS* in tomato AC plants.**

(A) Schematic view of the method for constructing the expression cassettes for the sgRNAs. Specific target 1 sequence can be inserted into between the promoter and the unchanged guide-RNA using *Bsa*I digestion. Promoter *AtU3d/LacZ* were used to drive the single *slyPDS-*targeted sgRNA. (B) pYLCRISPR/Cas9-slyPDS-T1 plasmid prepared for *Agrobacterium*-mediated transformation. (C-F) Typical albino phenotypes of the transgenic AC lines regenerated from callus. (G) Mutant alleles identified in regenerated AC lines in (C-F). Red letters indicate the target site, minus symbols represent deletions, blue letters represent base substitutions.

**
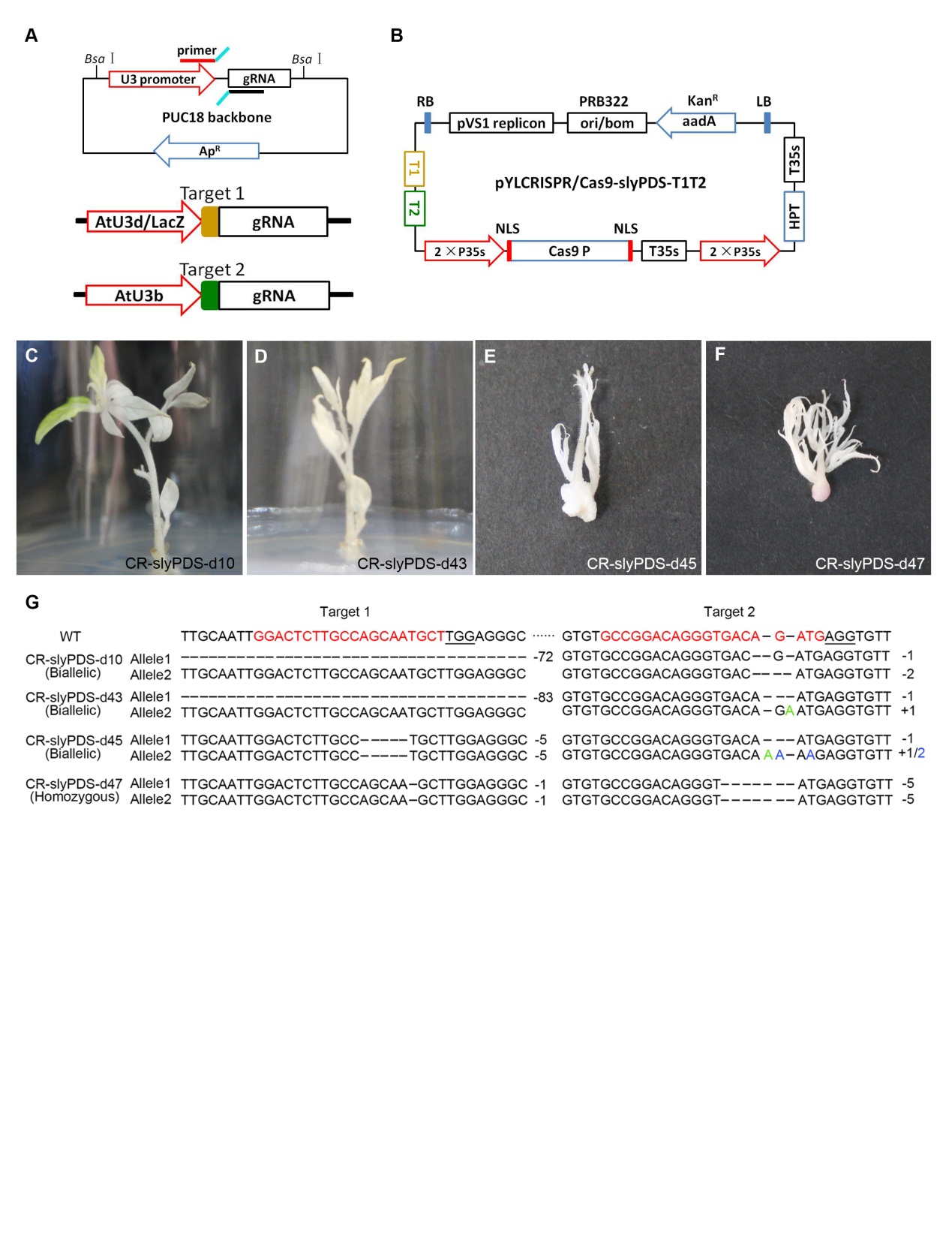
**

**Figure S2. Genome editing of *slyPDS* in tomato MT plants.**

(A) Schematic view of the method for constructing the expression cassettes for the sgRNAs. Specific target 1 and 2 sequence can be inserted into between the promoter and the unchanged guide-RNA using *Bsa*I digestion. Promoters *AtU3d/LacZ* and *AtU3b* were used to drive the different *slyPDS-*targeted sgRNAs, respectively. (B) pYLCRISPR/Cas9-slyPDS-T1T2 plasmid prepared for *Agrobacterium*-mediated transformation. (C-F) Typical albino phenotypes of the transgenic MT lines regenerated from callus. (G) Mutant alleles identified in regenerated MT lines in (C-F). Red letters indicate the target site, minus symbols represent deletions, blue letters represent base substitutions, green letters represent base insertion.

**
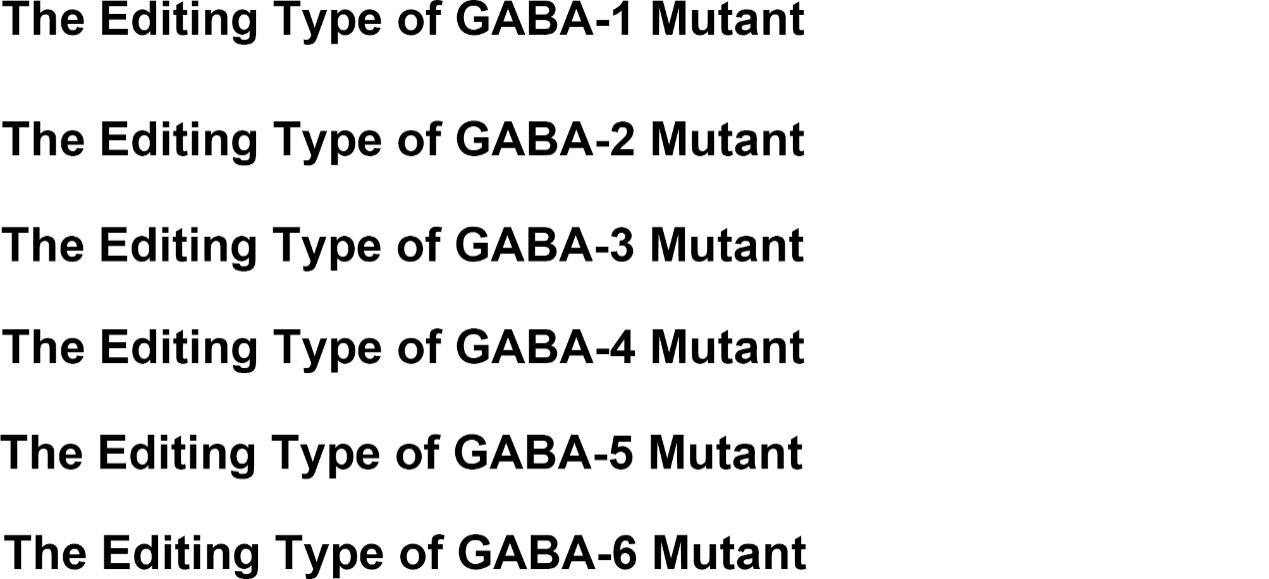
**

**
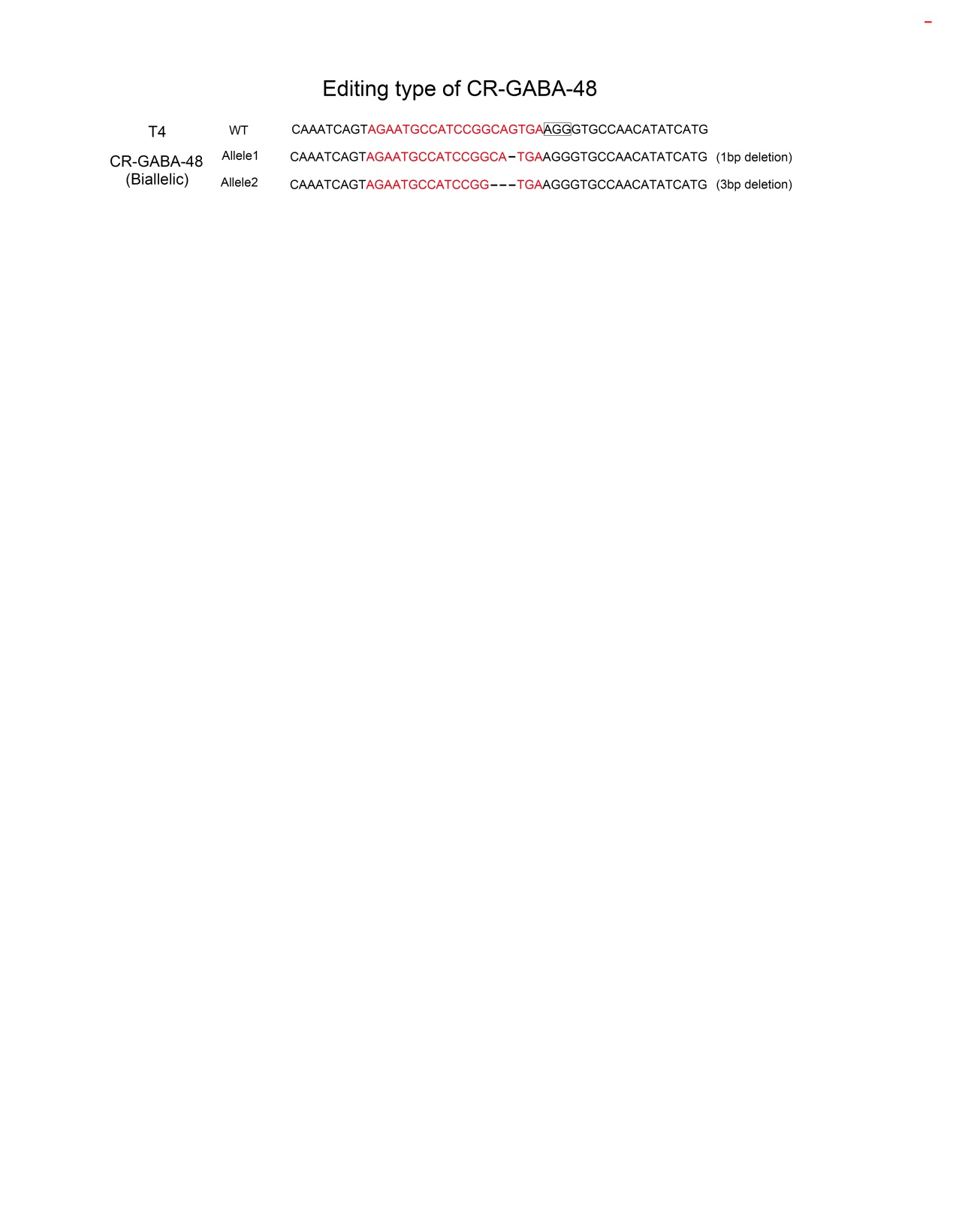
**

**
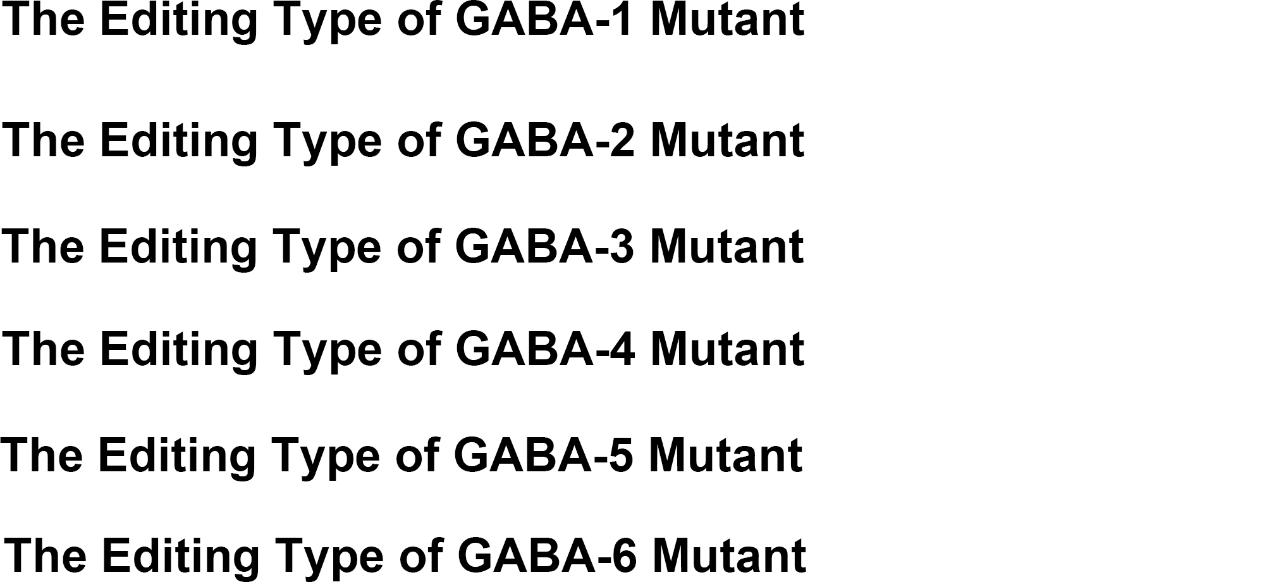

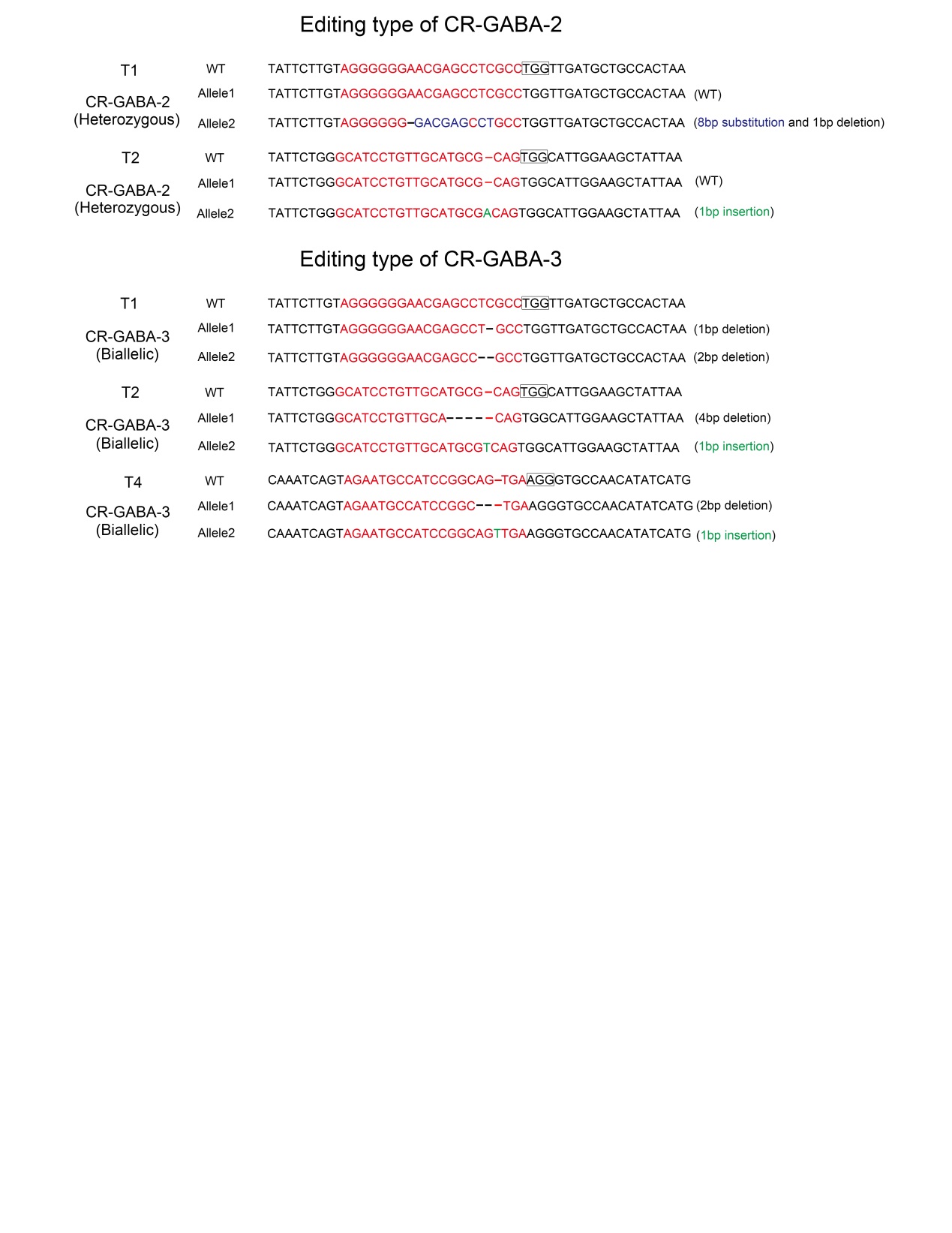

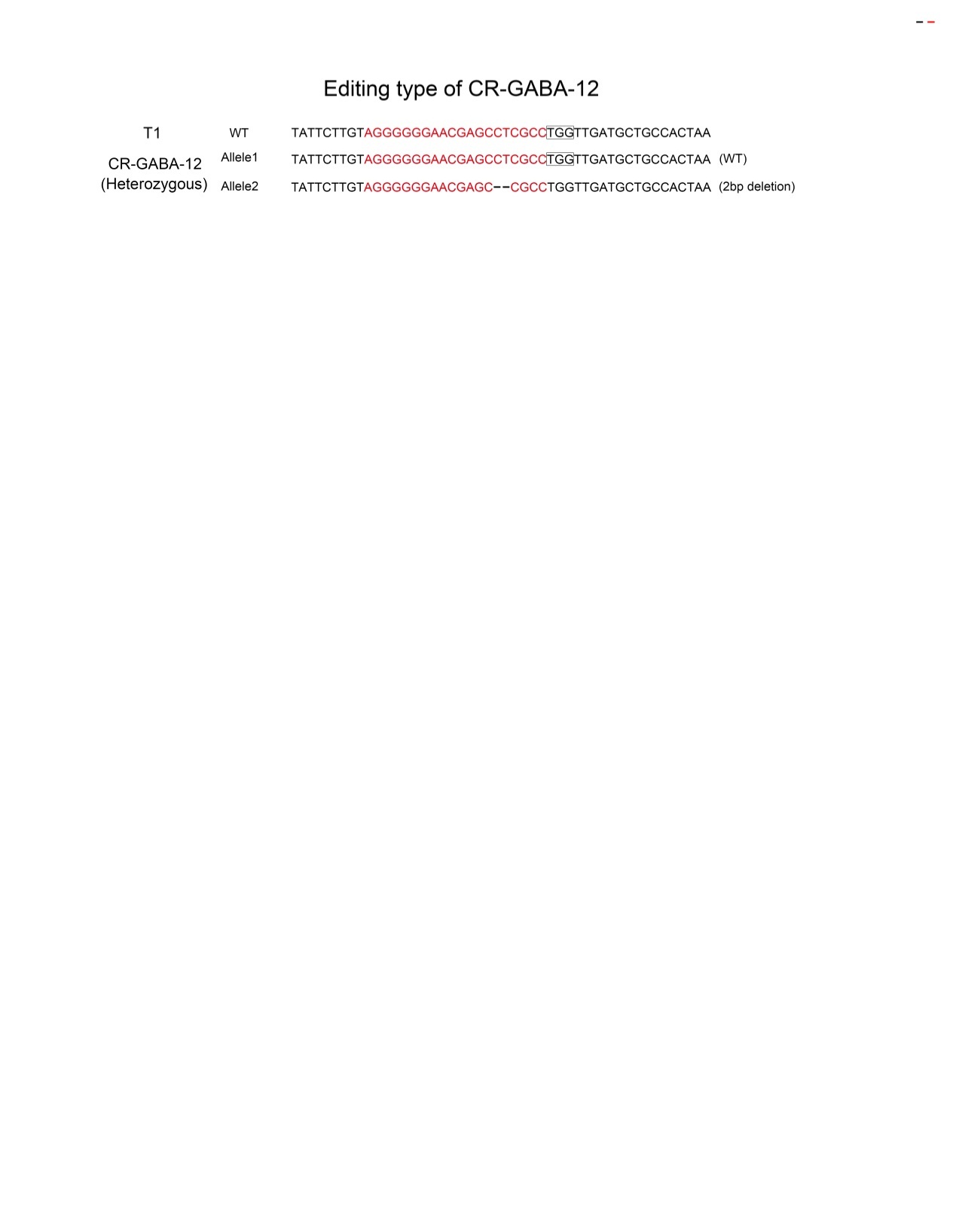

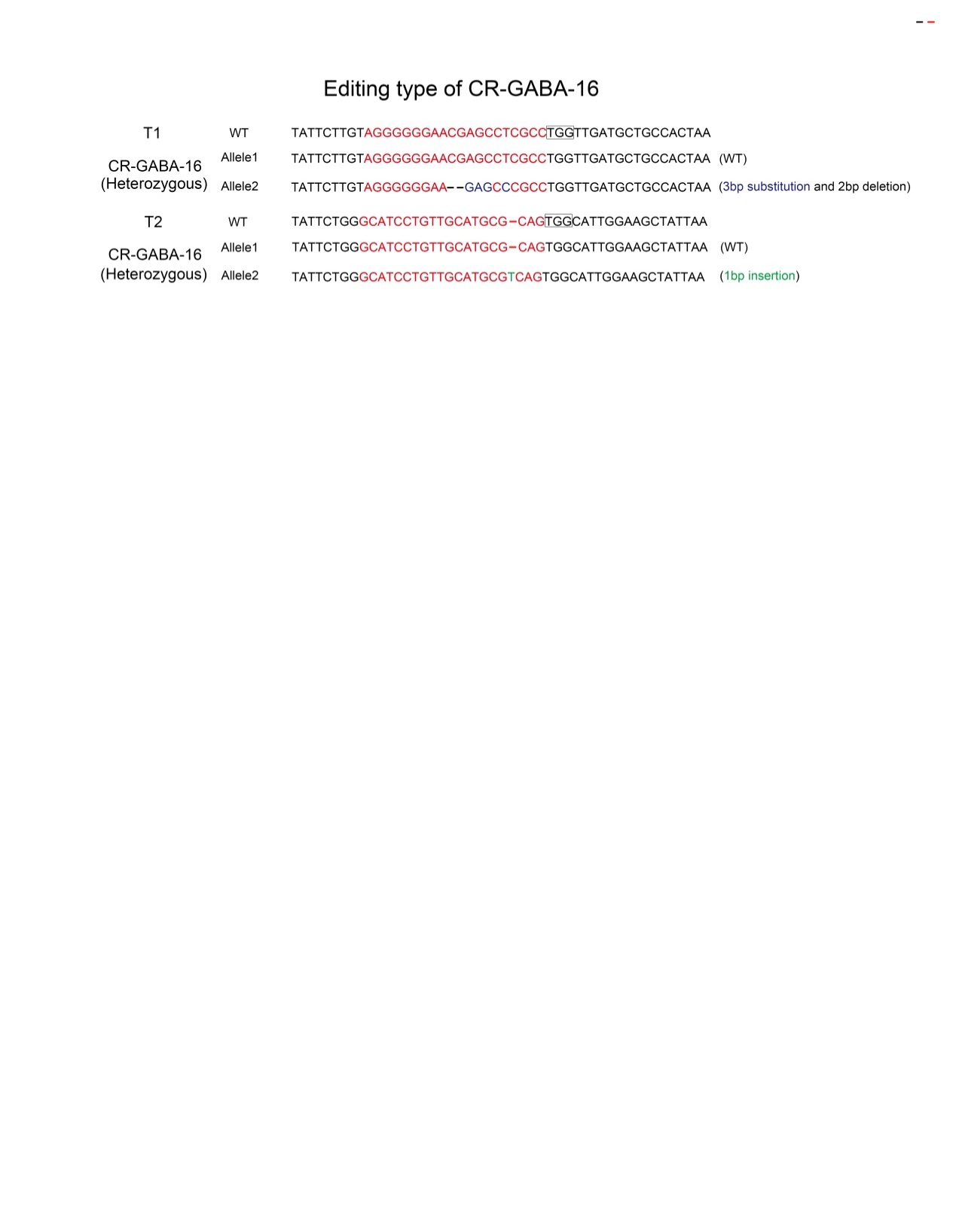
**
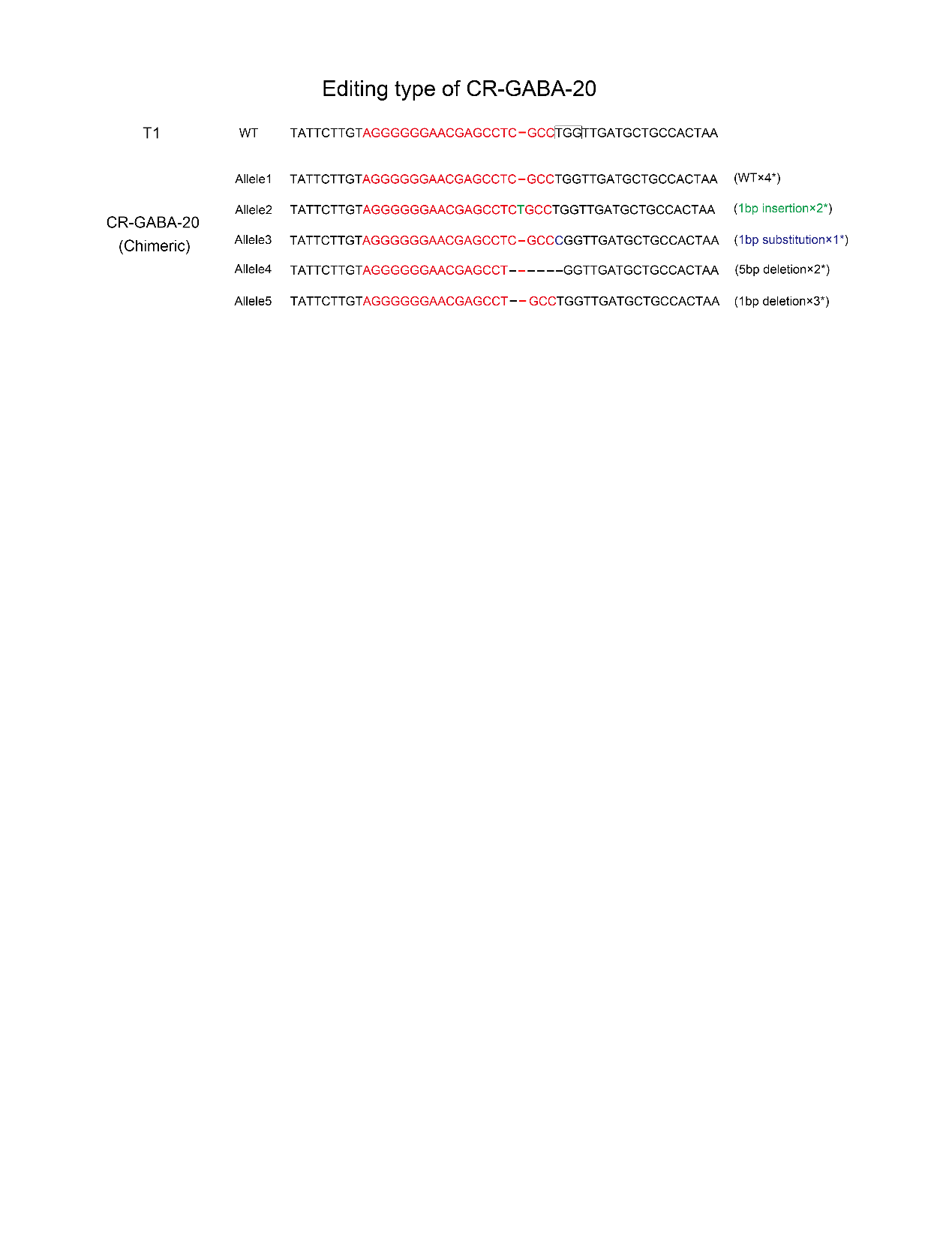
**
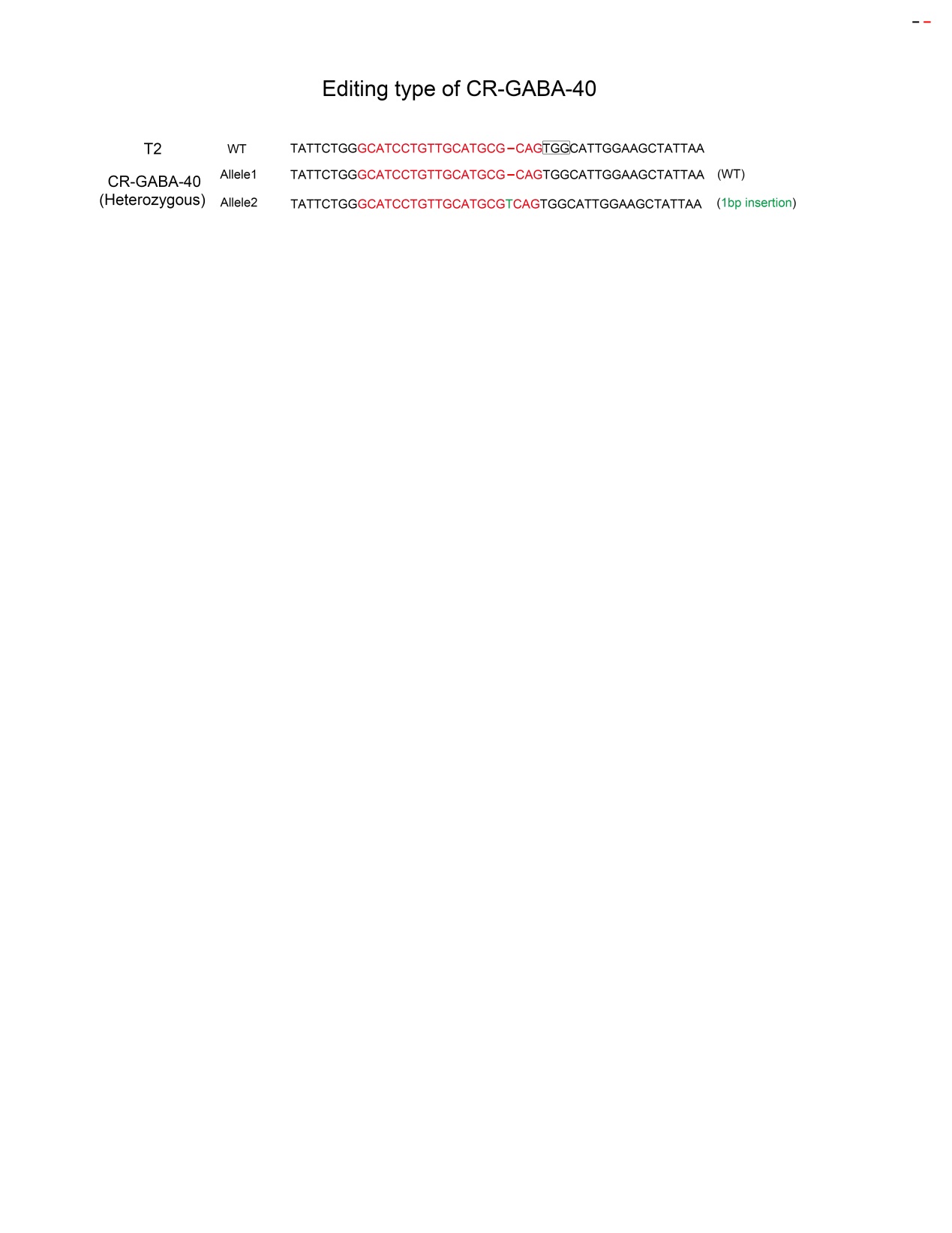

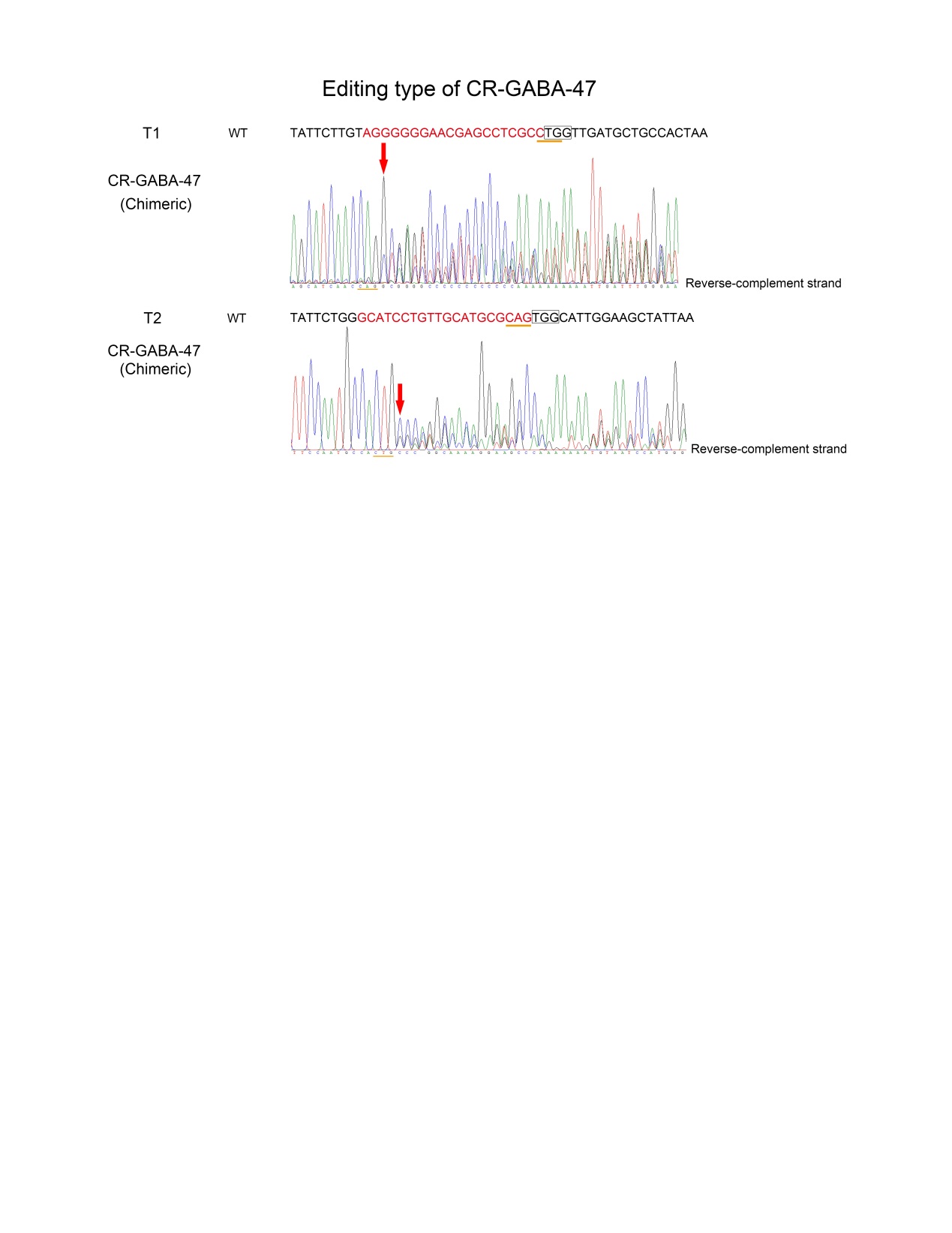

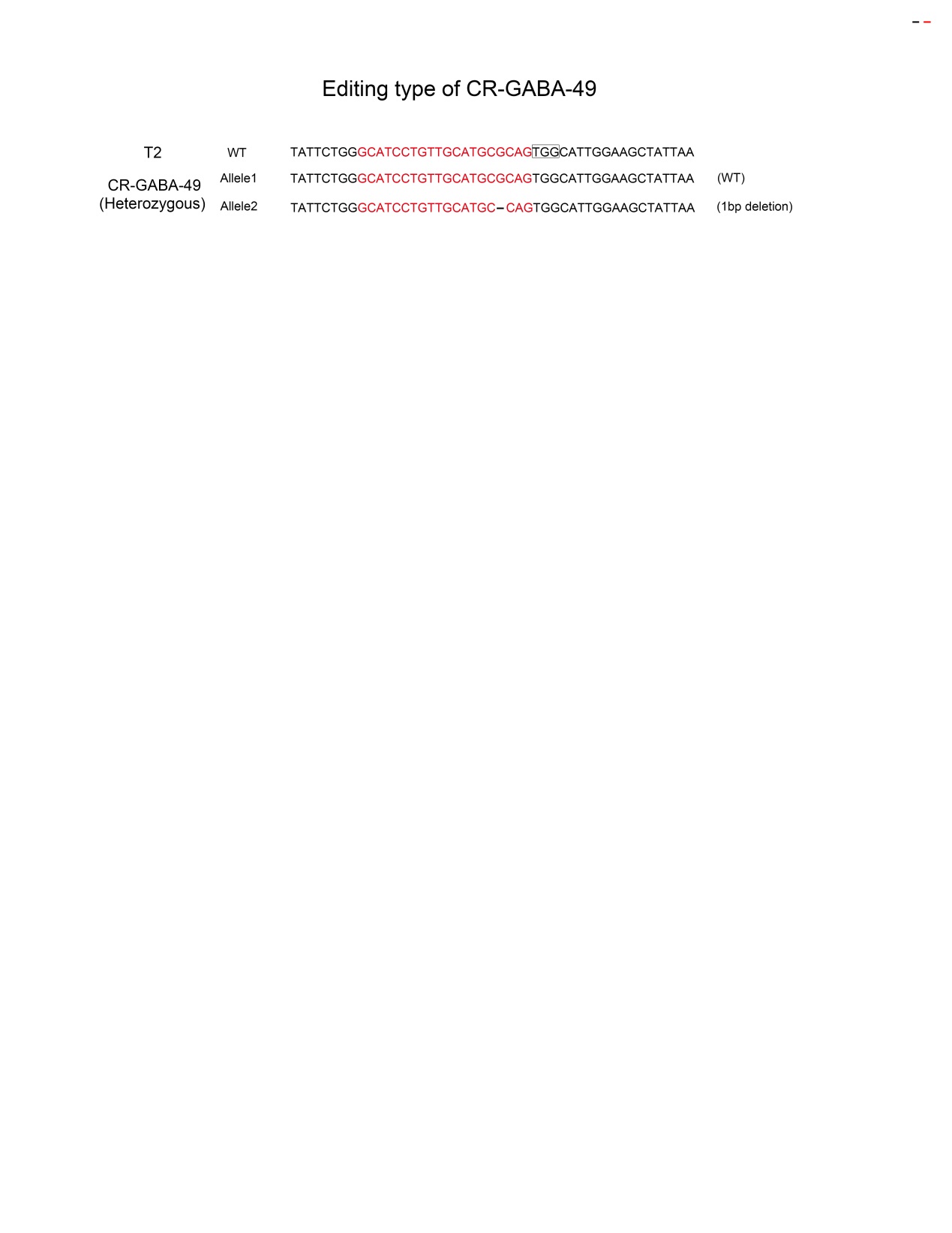

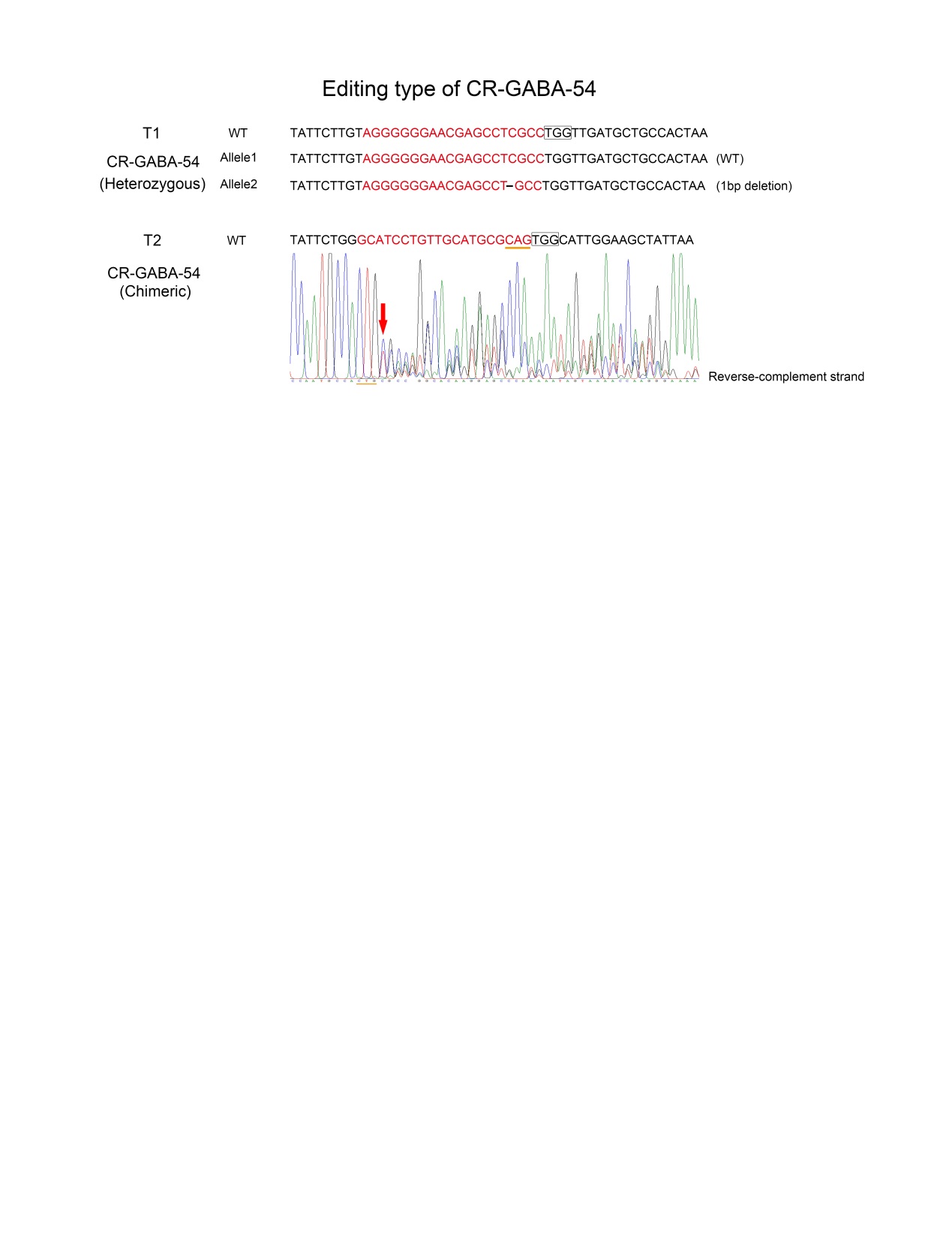

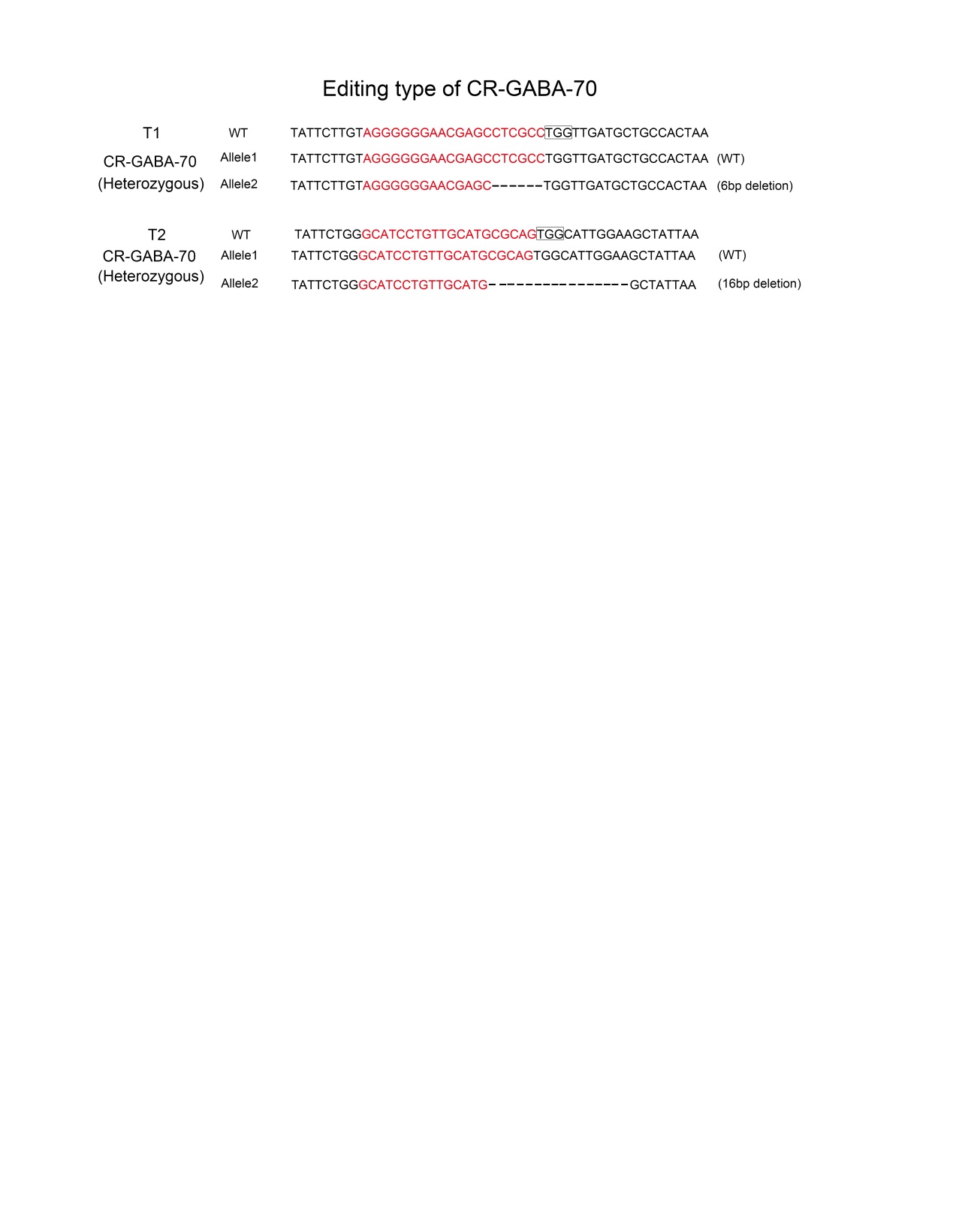

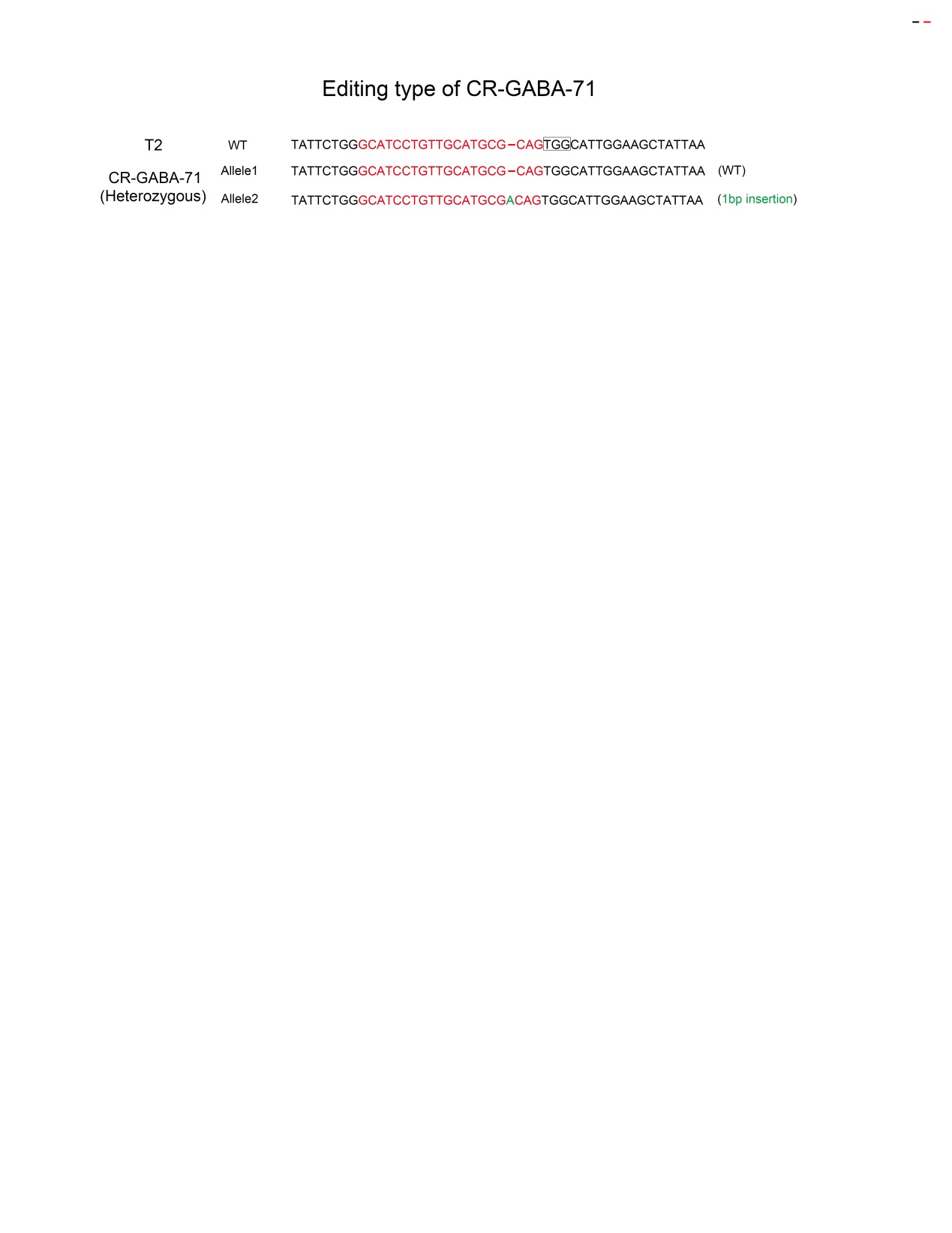

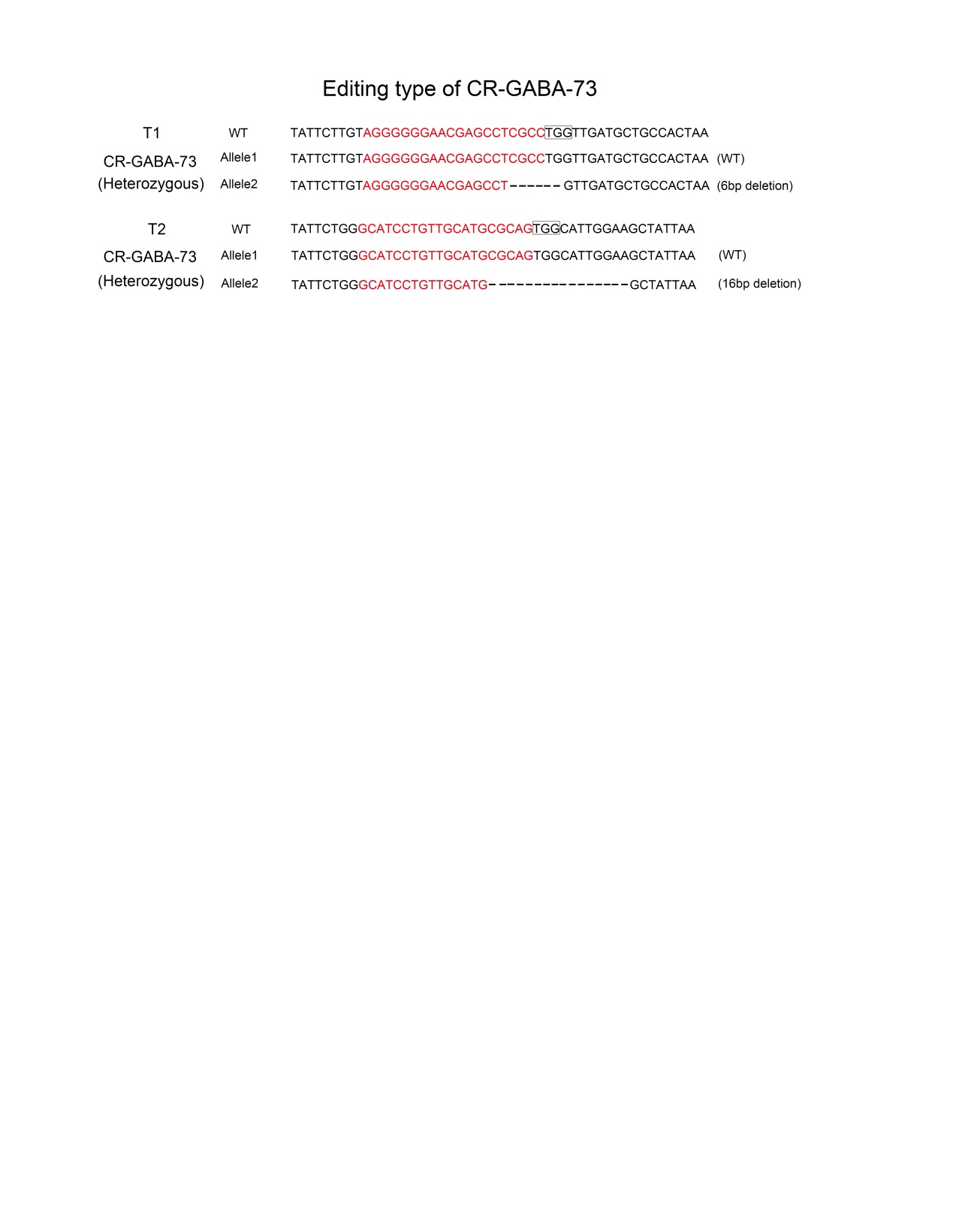

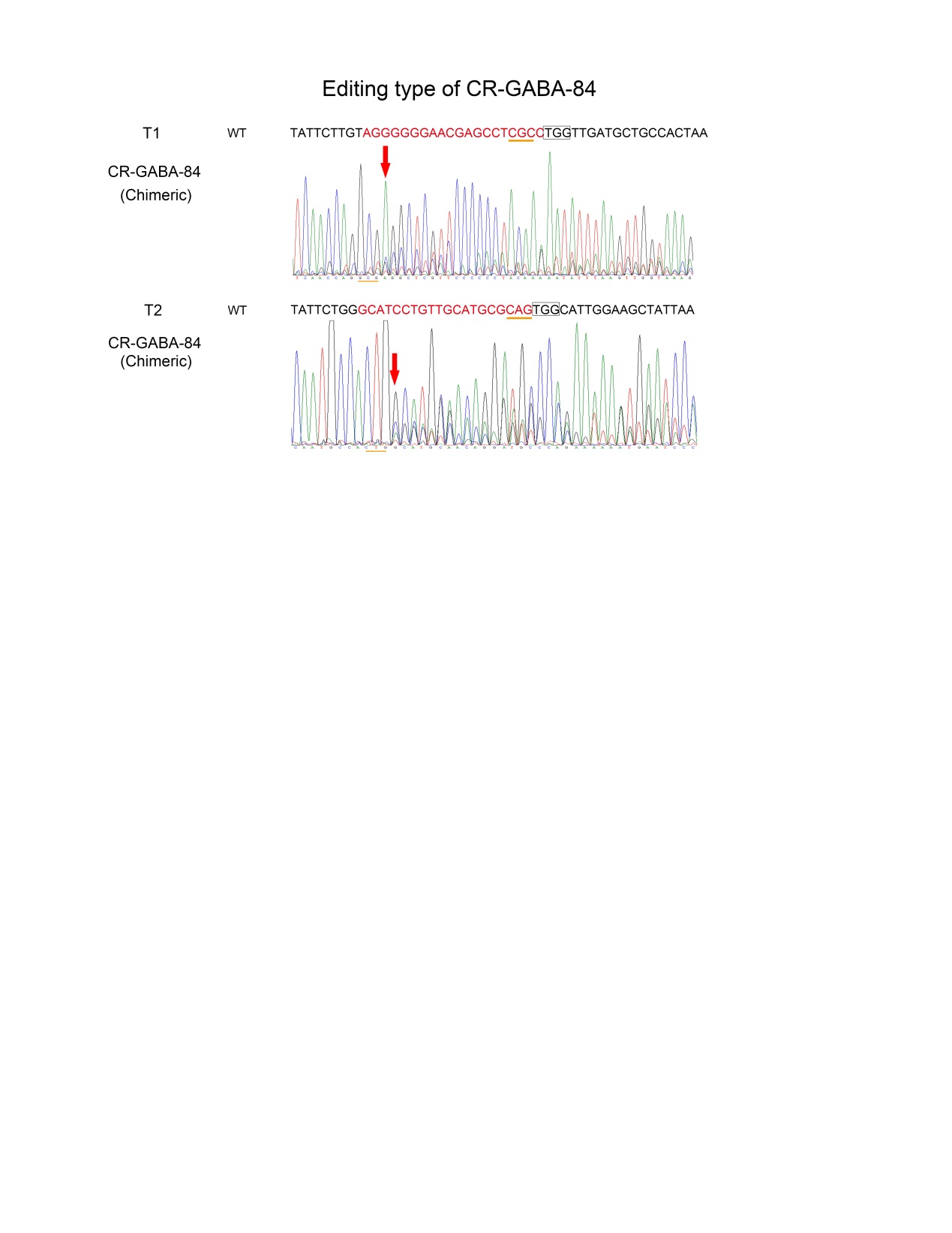
**

**
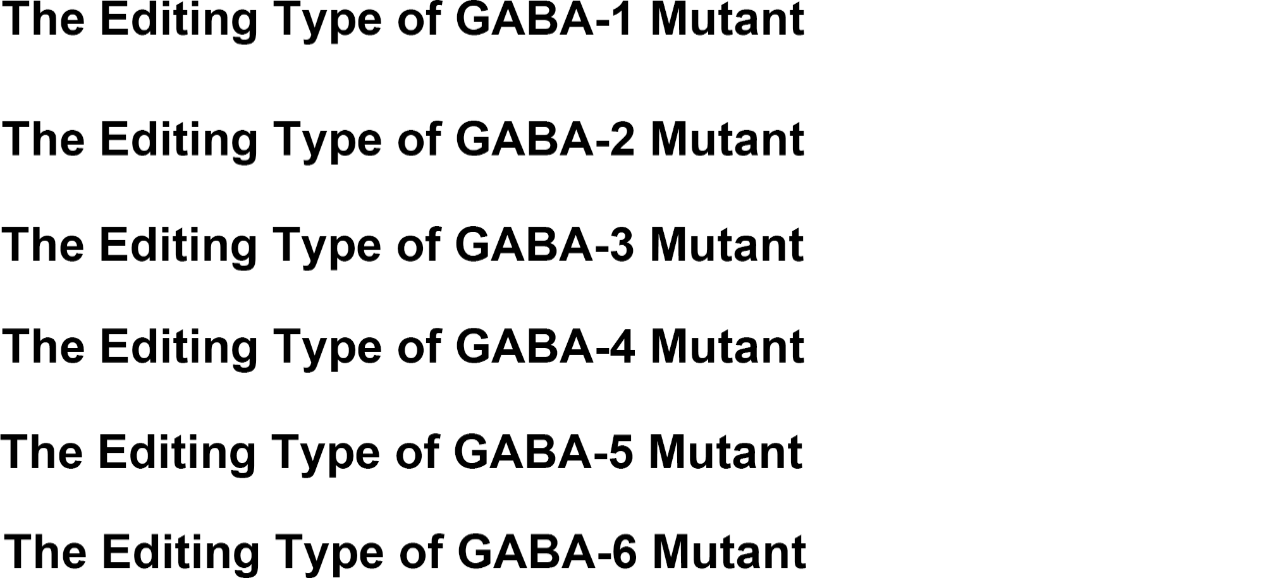

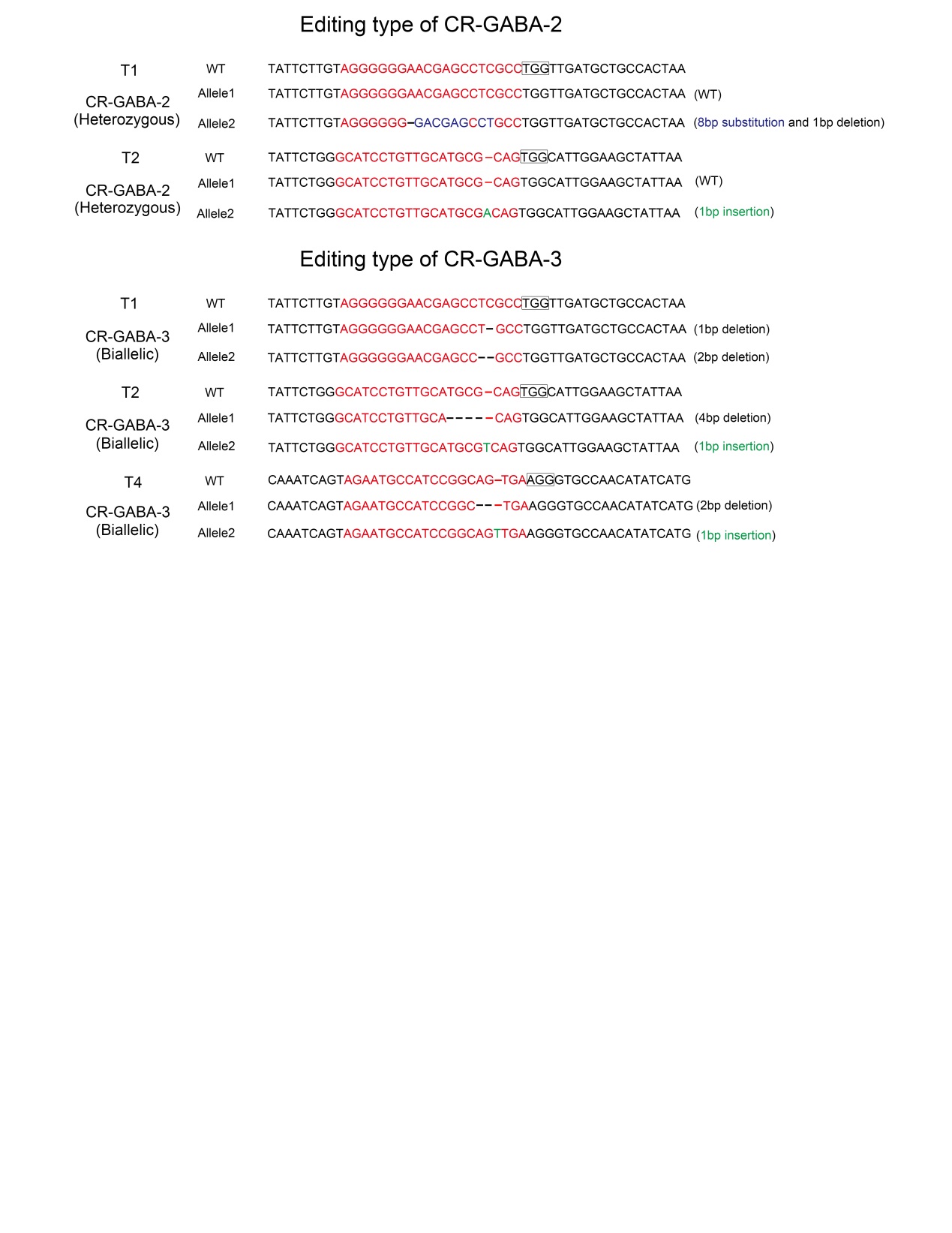

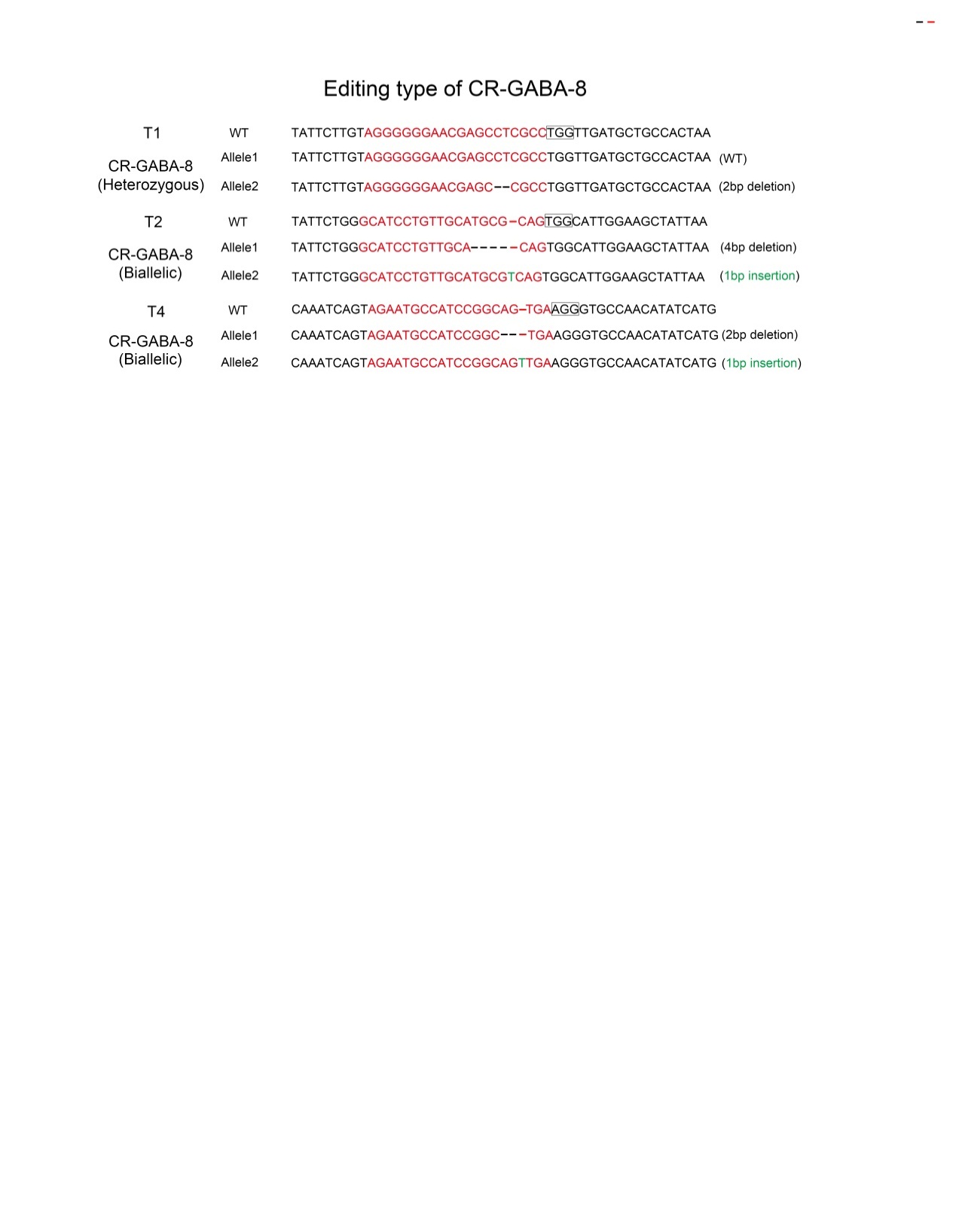
**

**
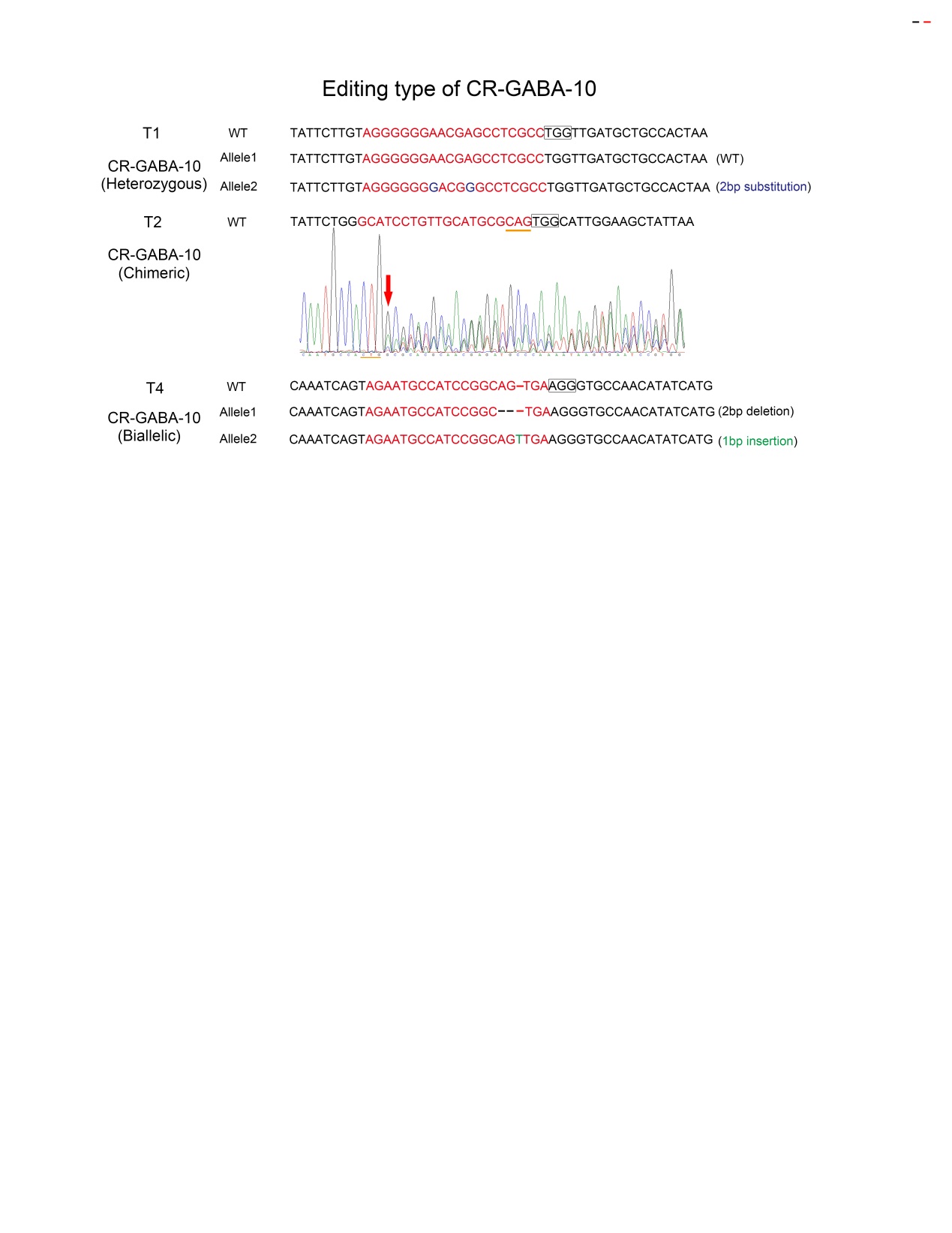

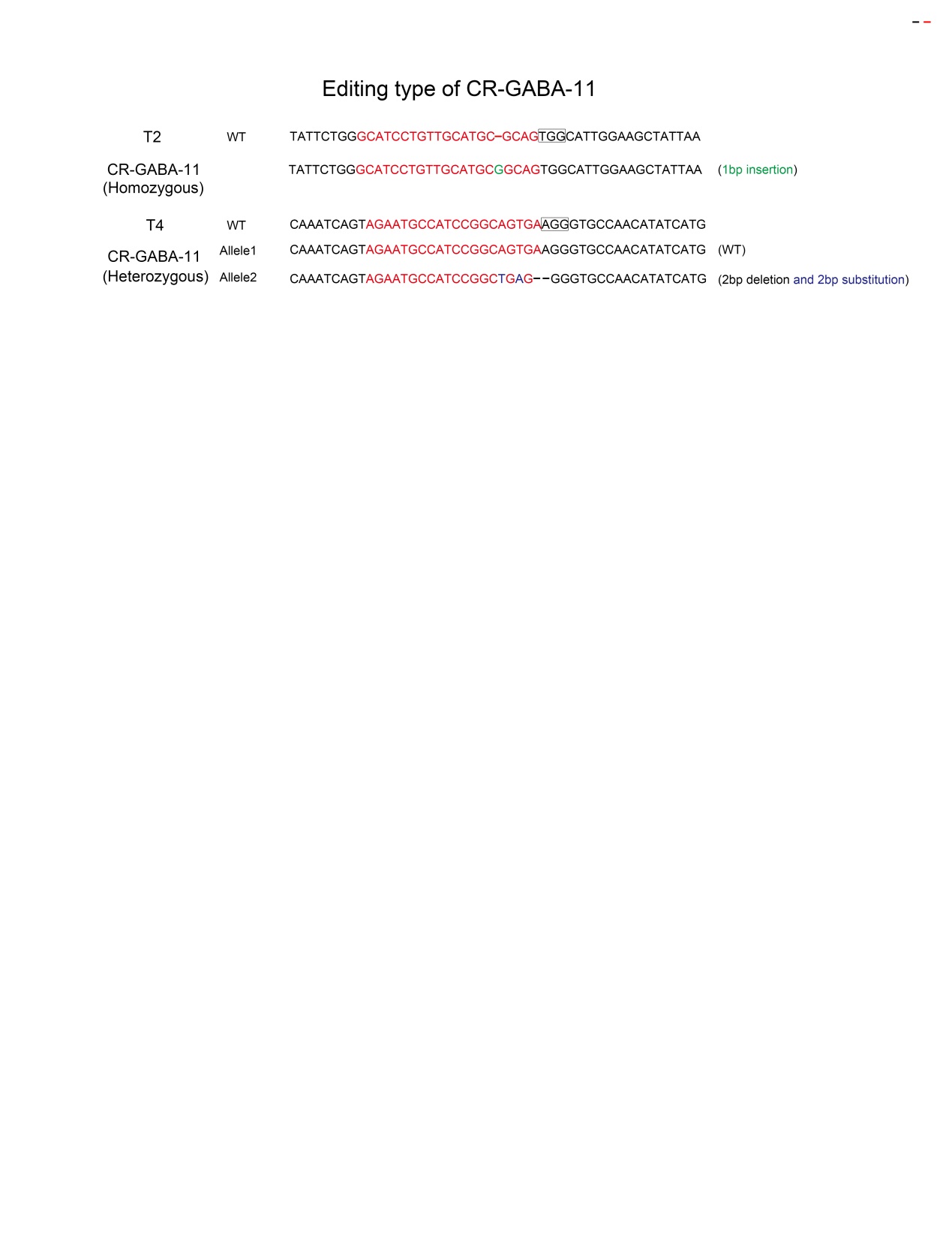

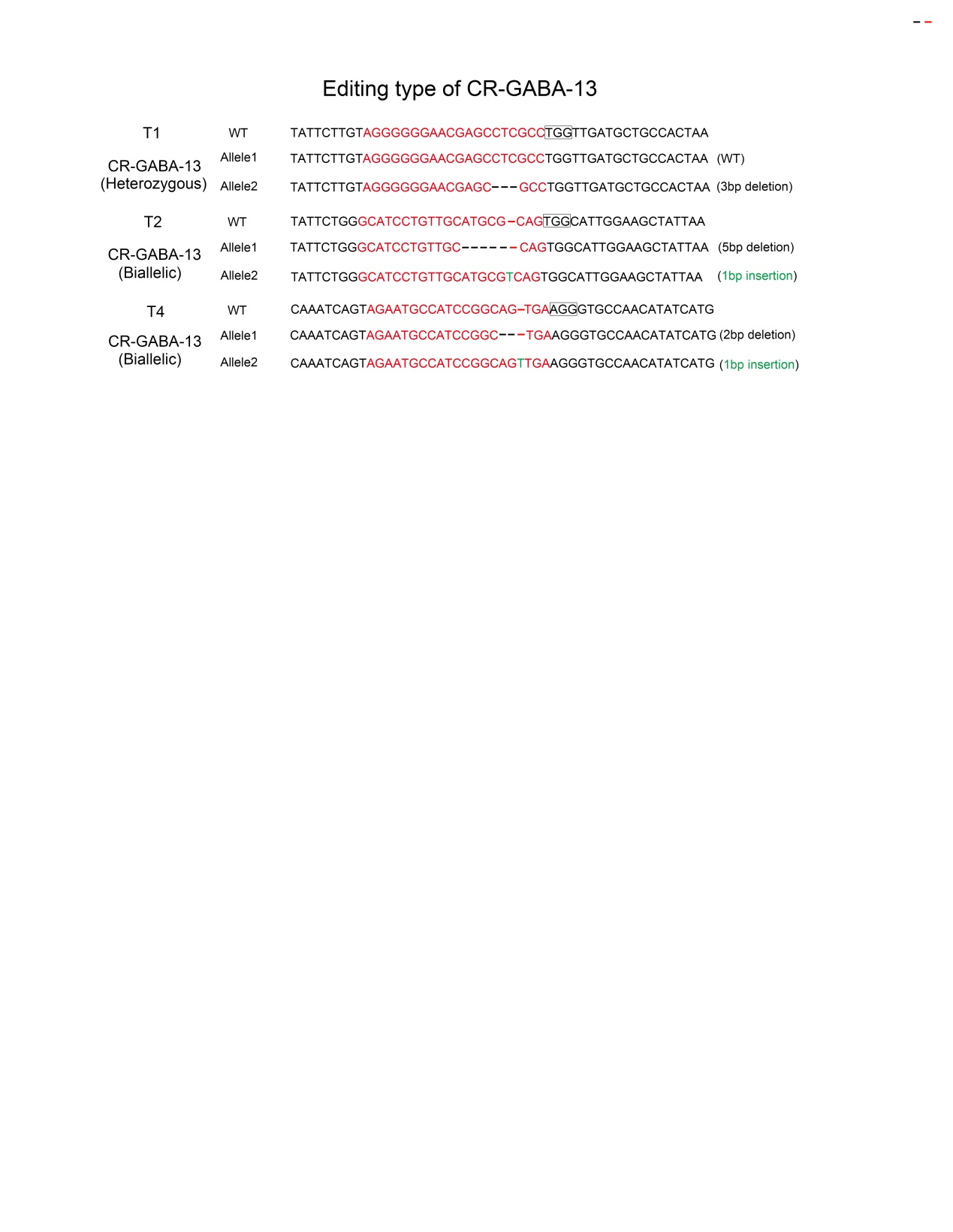

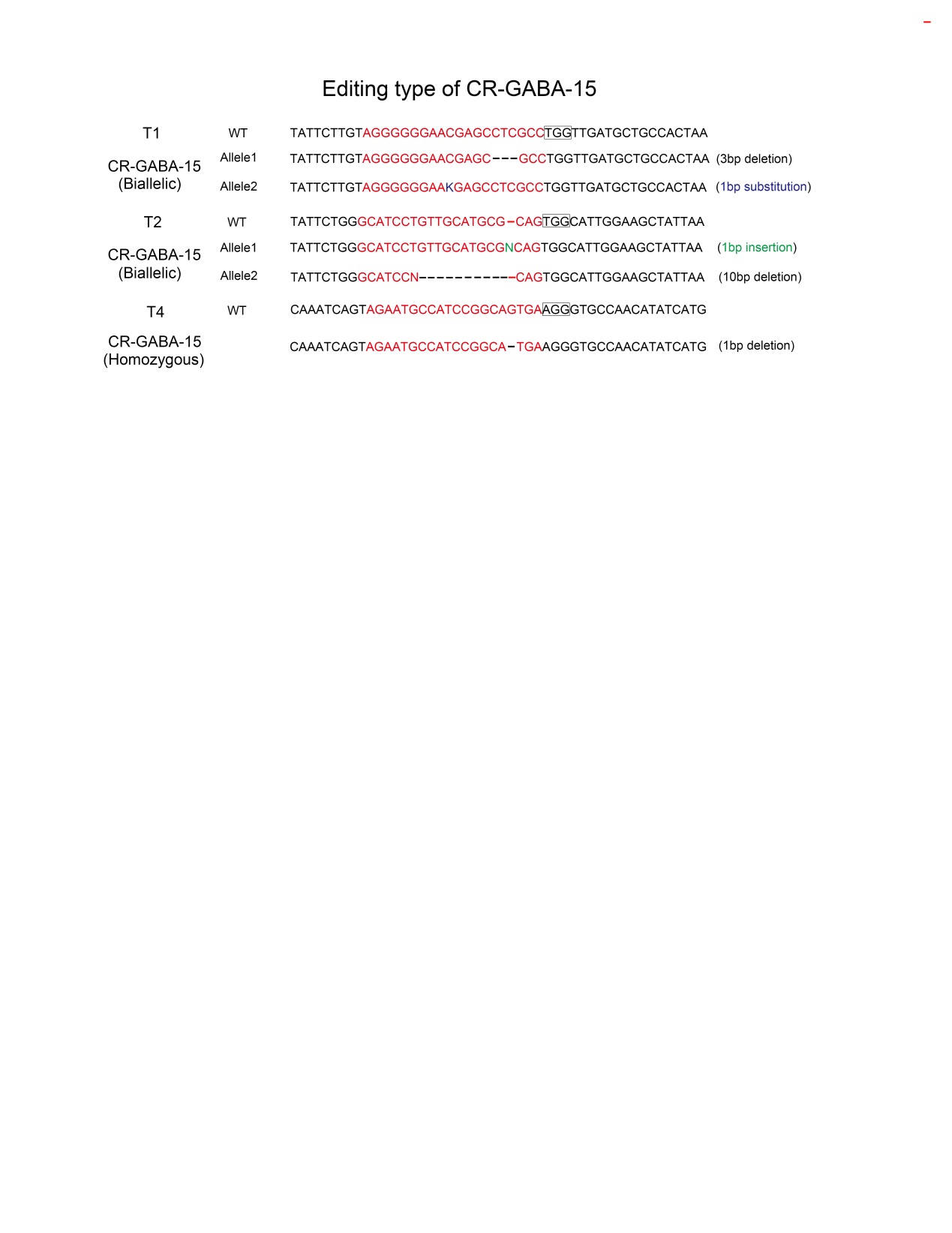

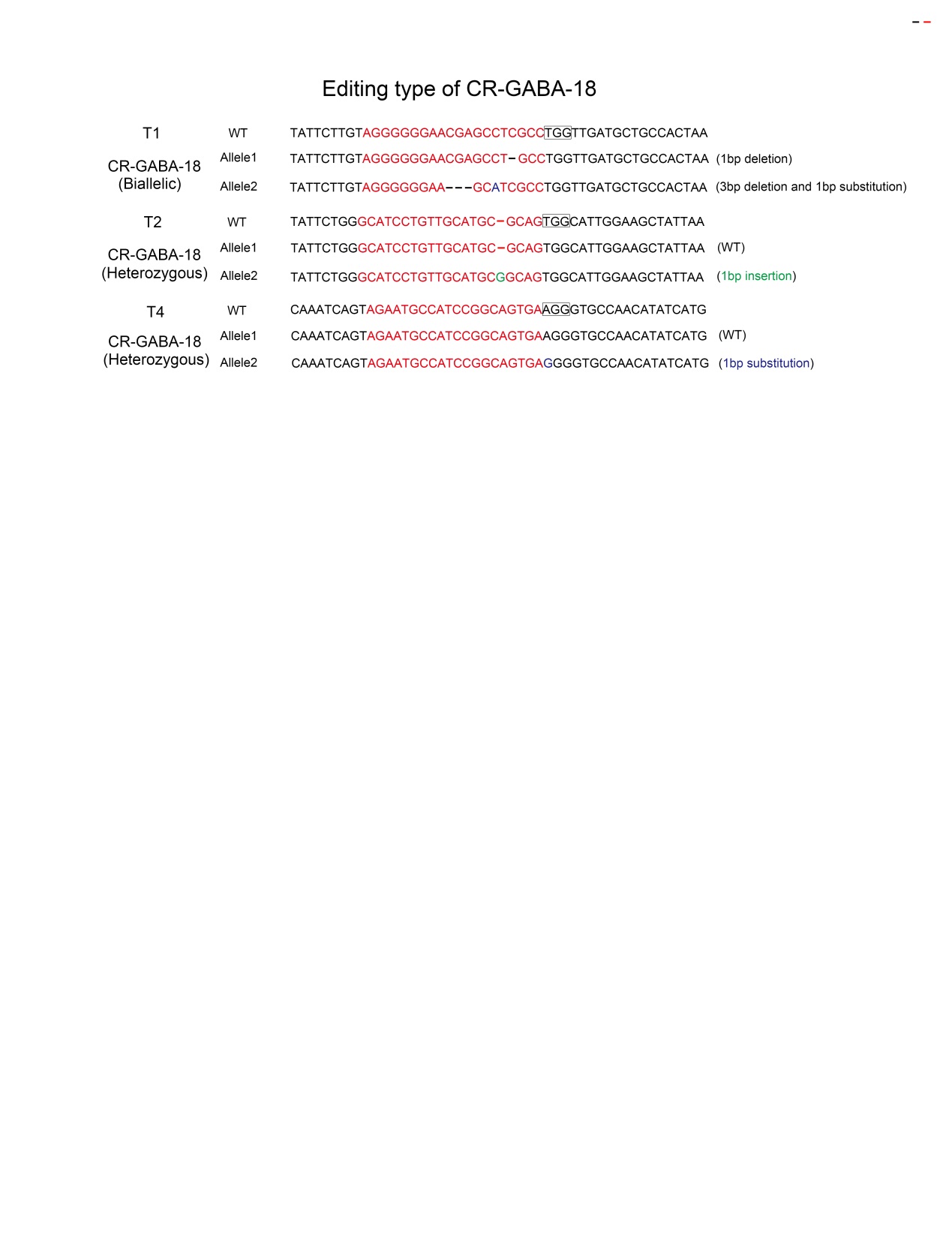

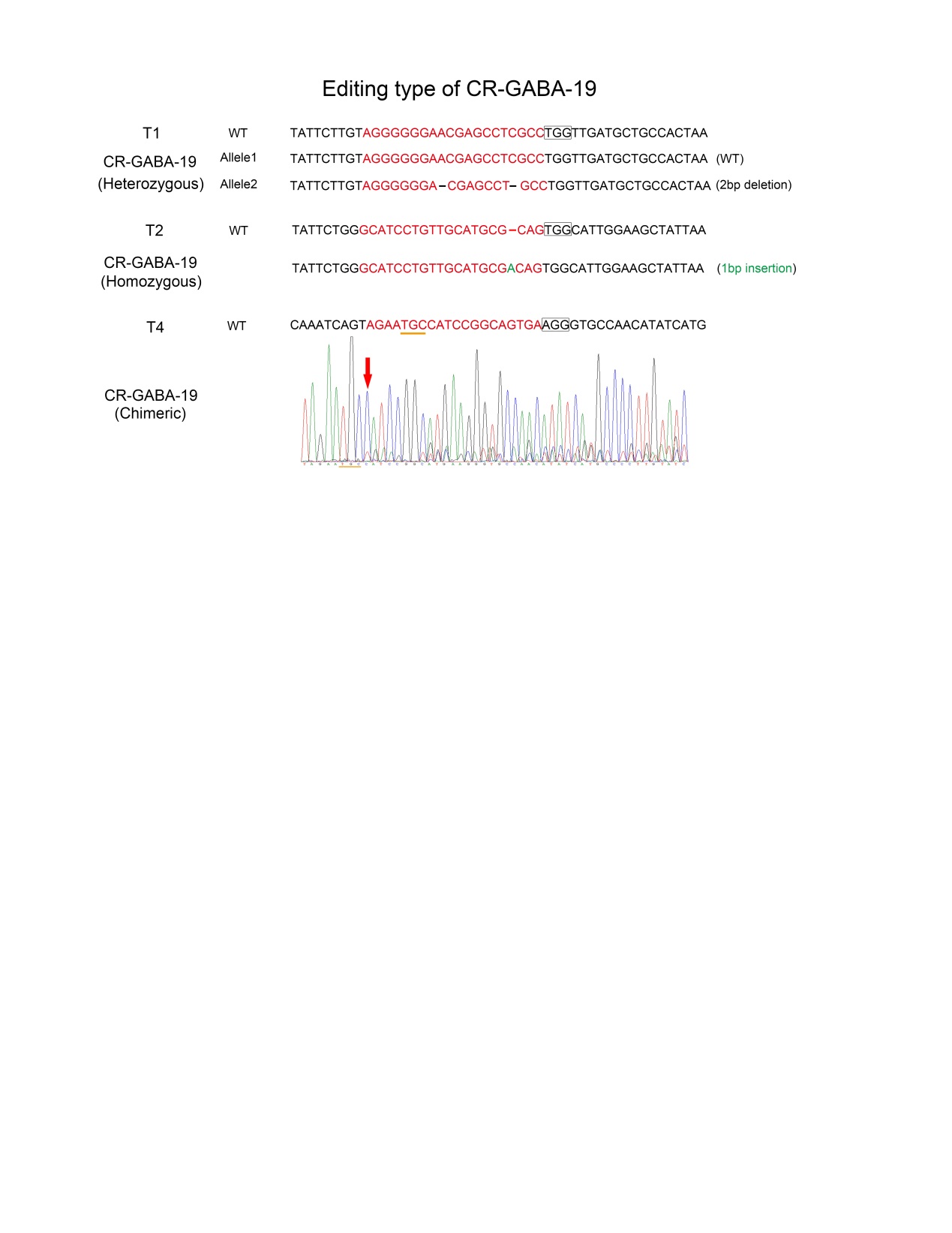

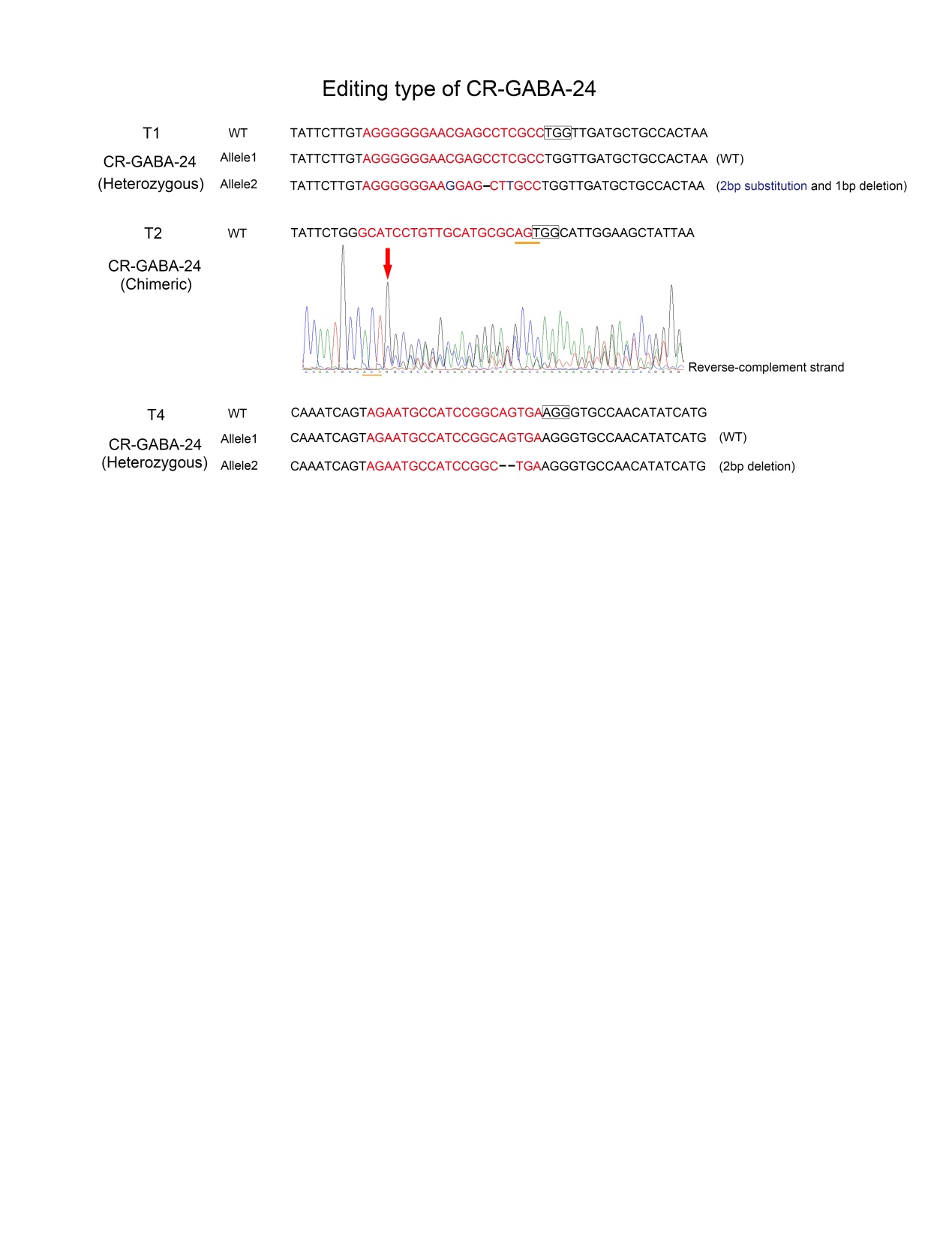

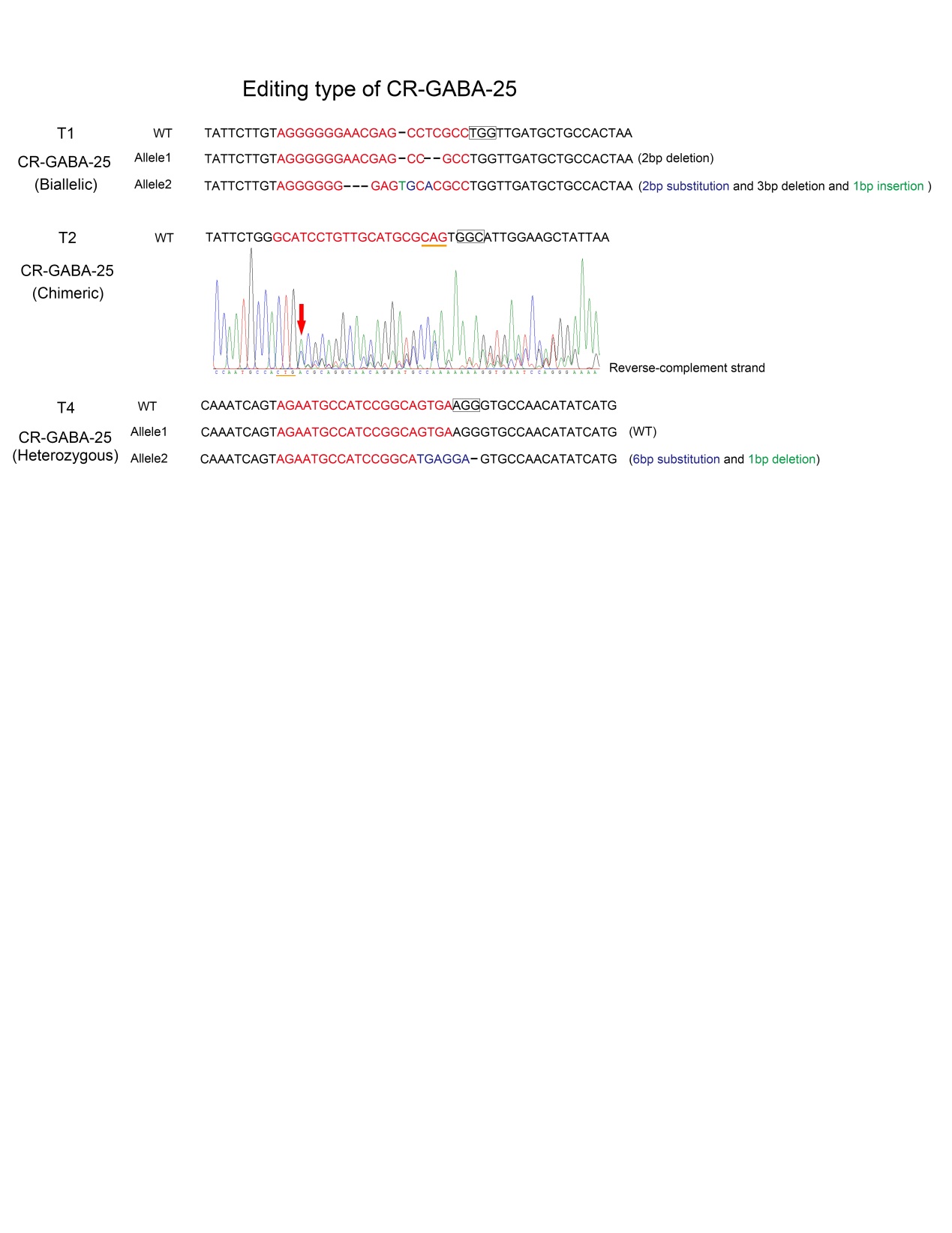

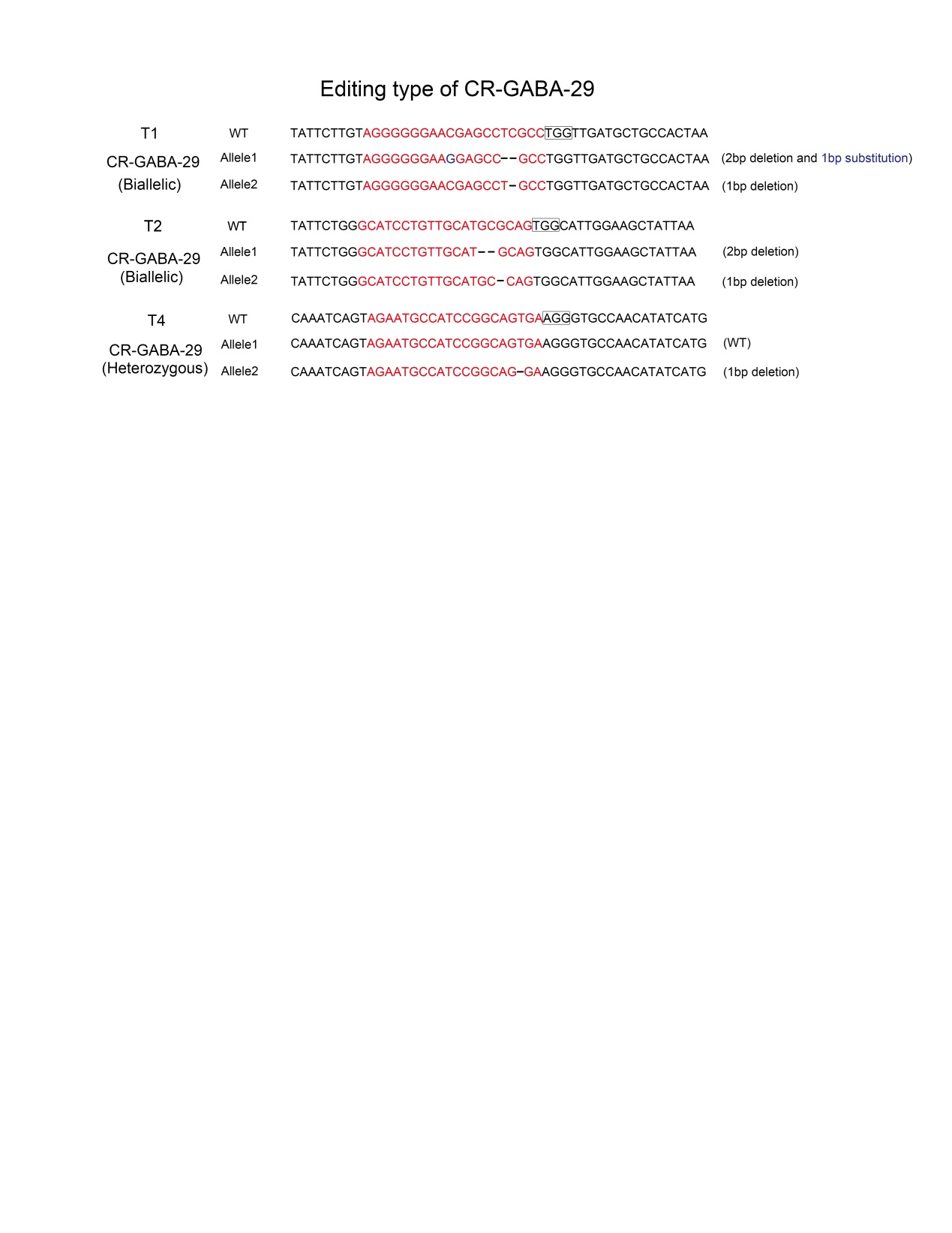

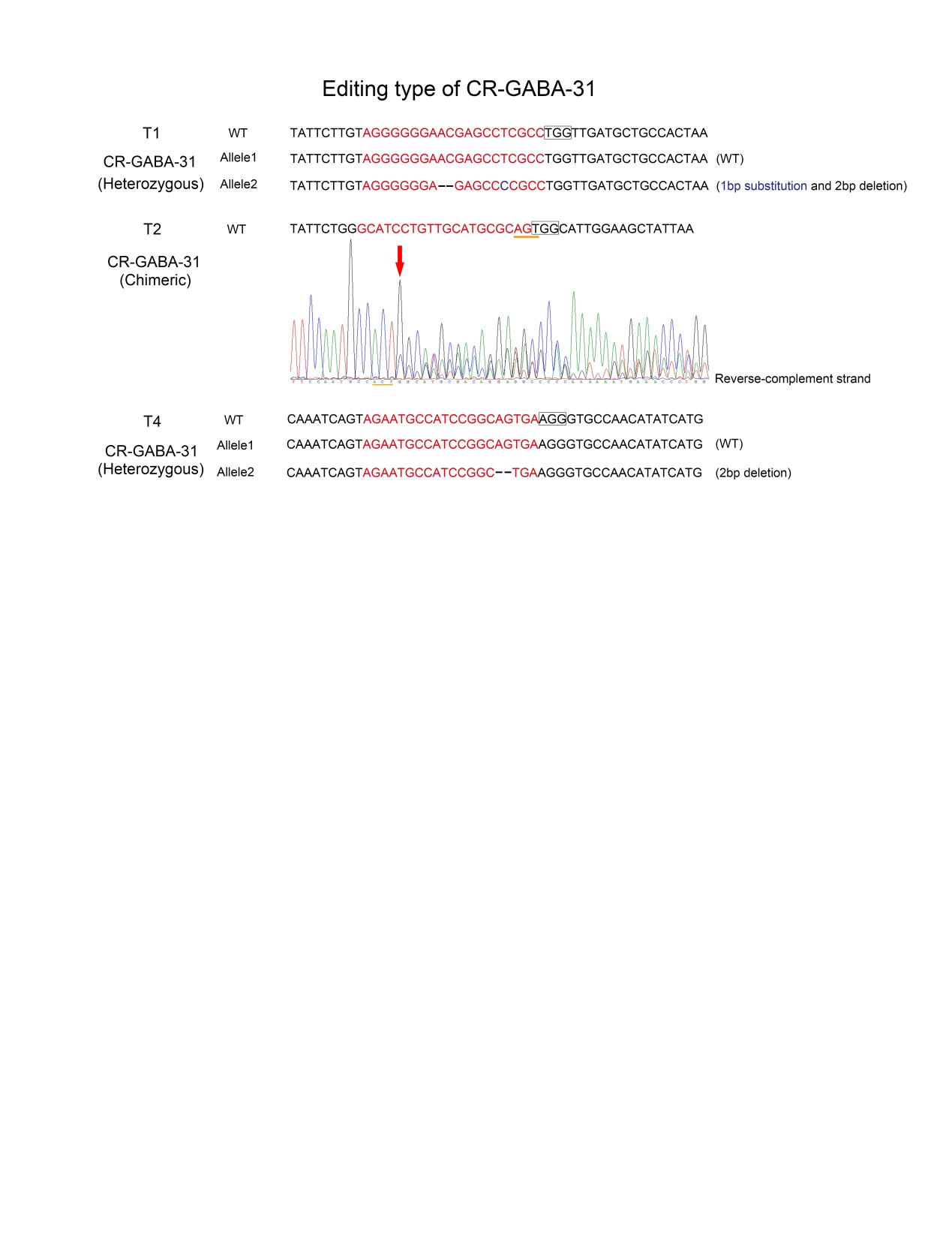

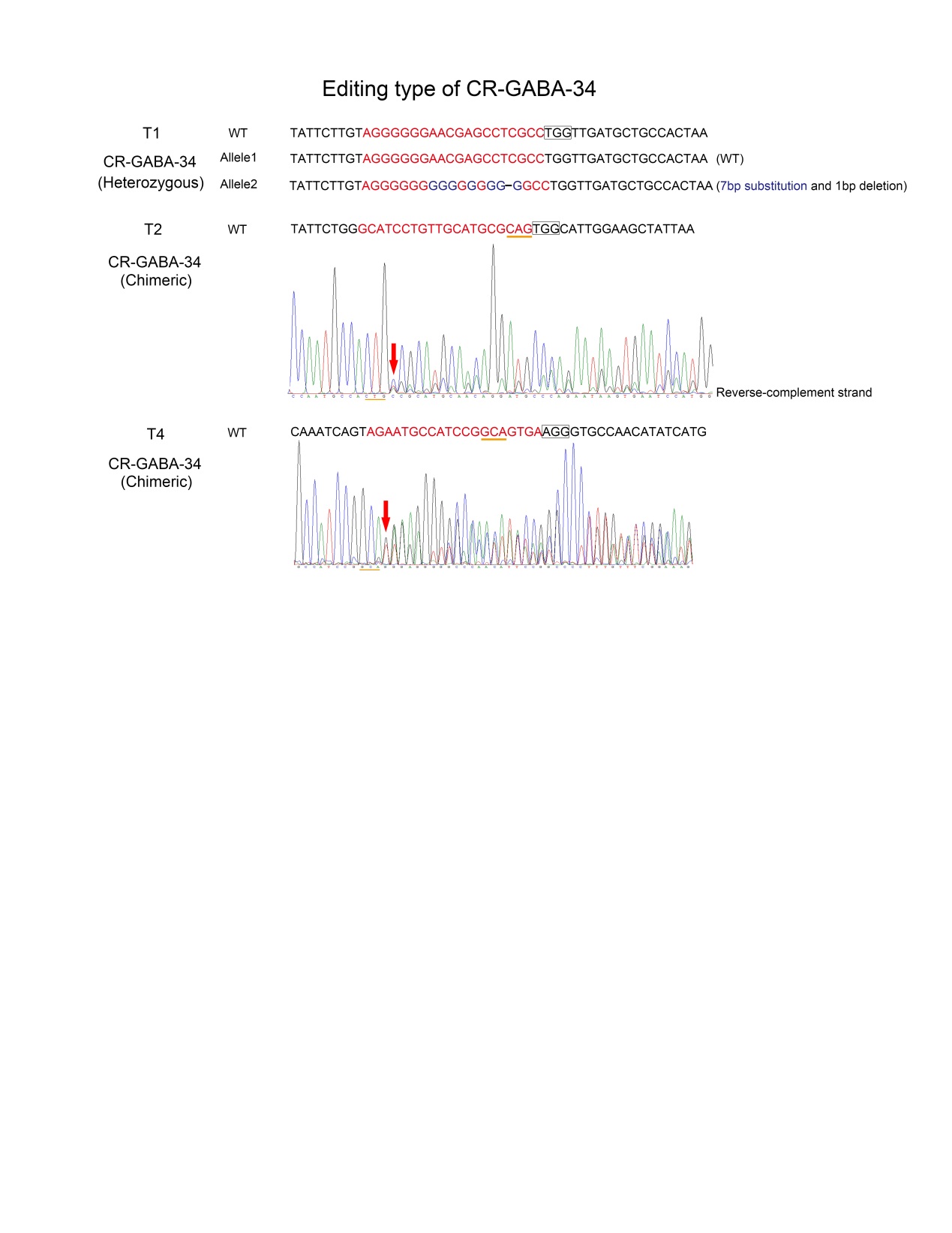

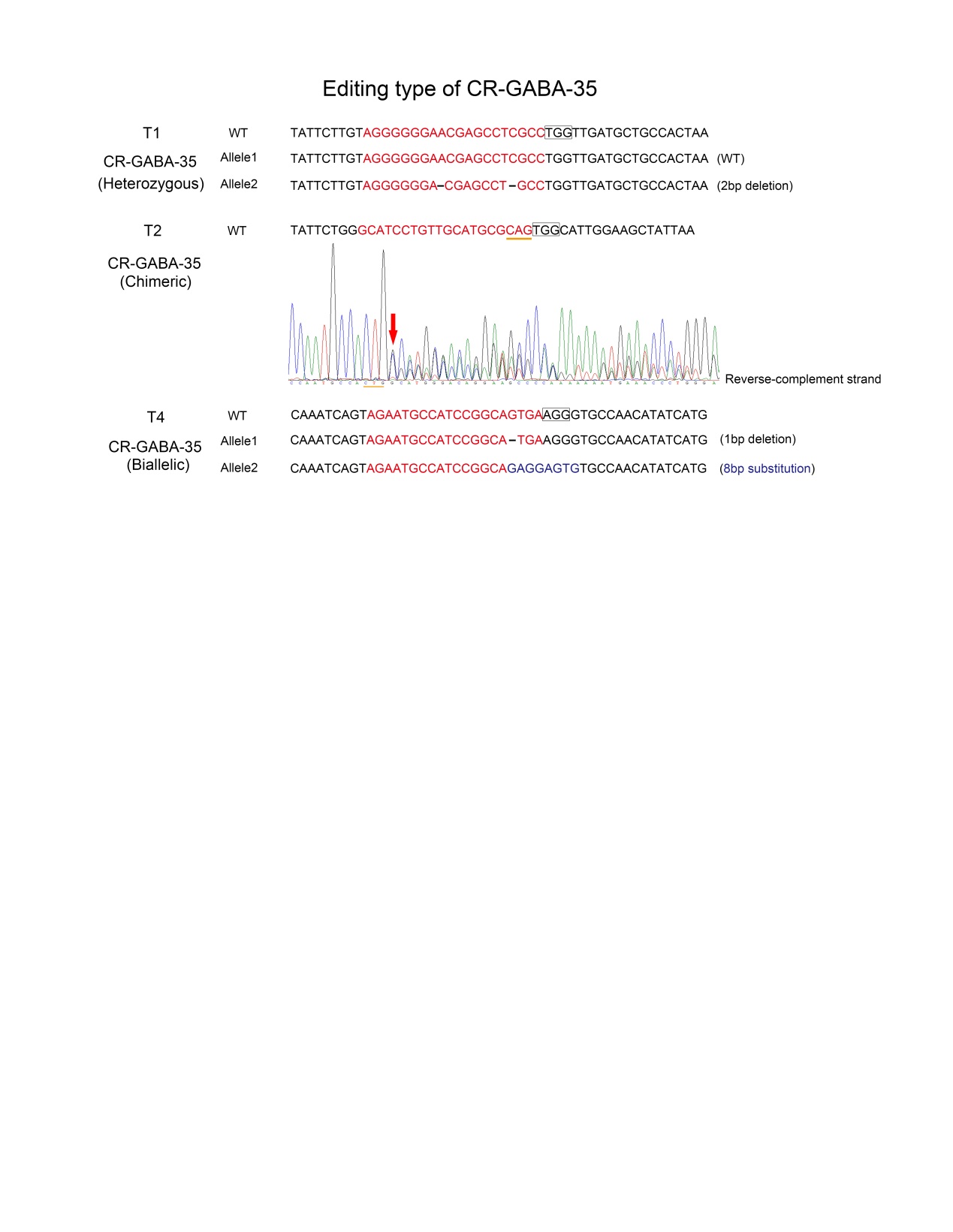

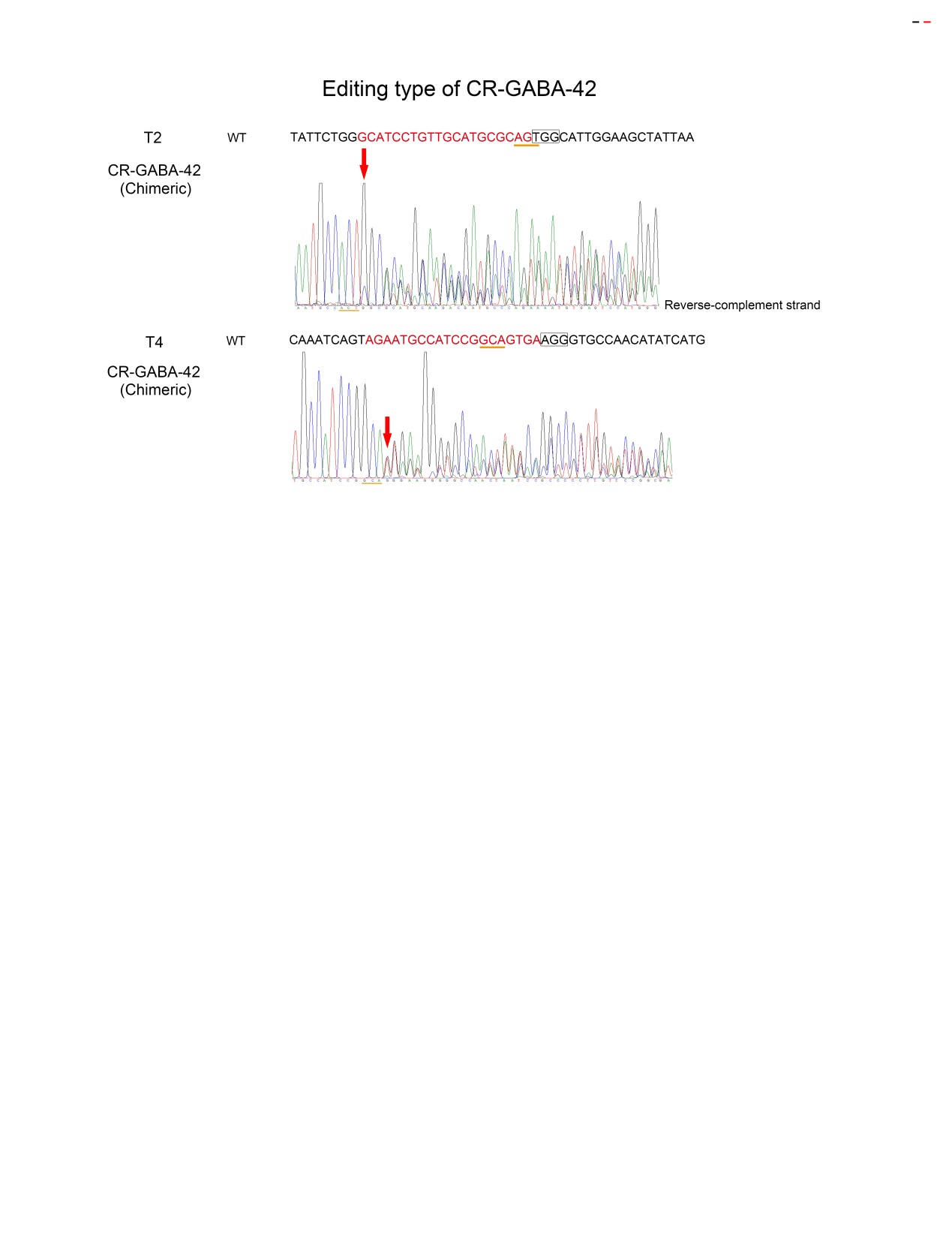

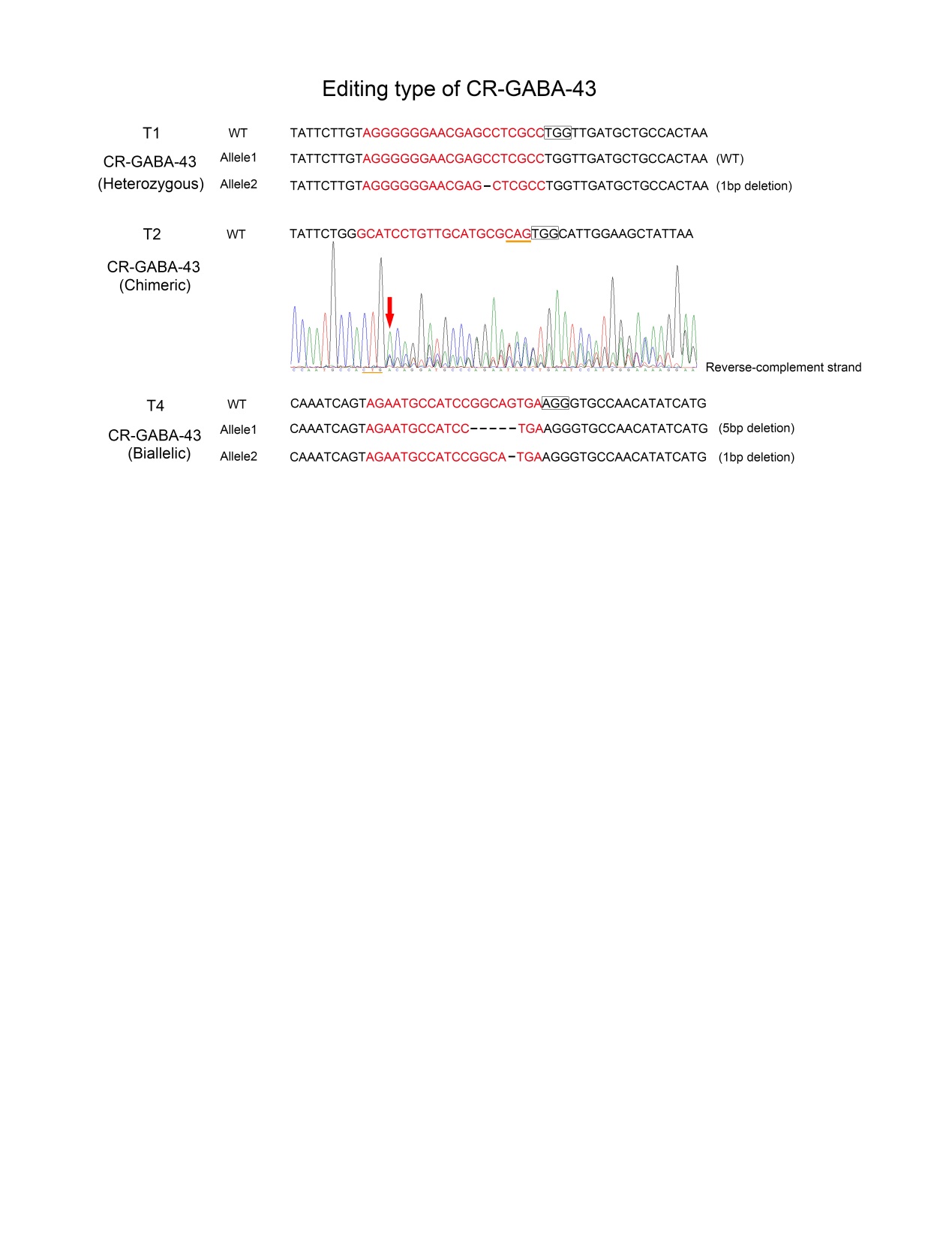
**
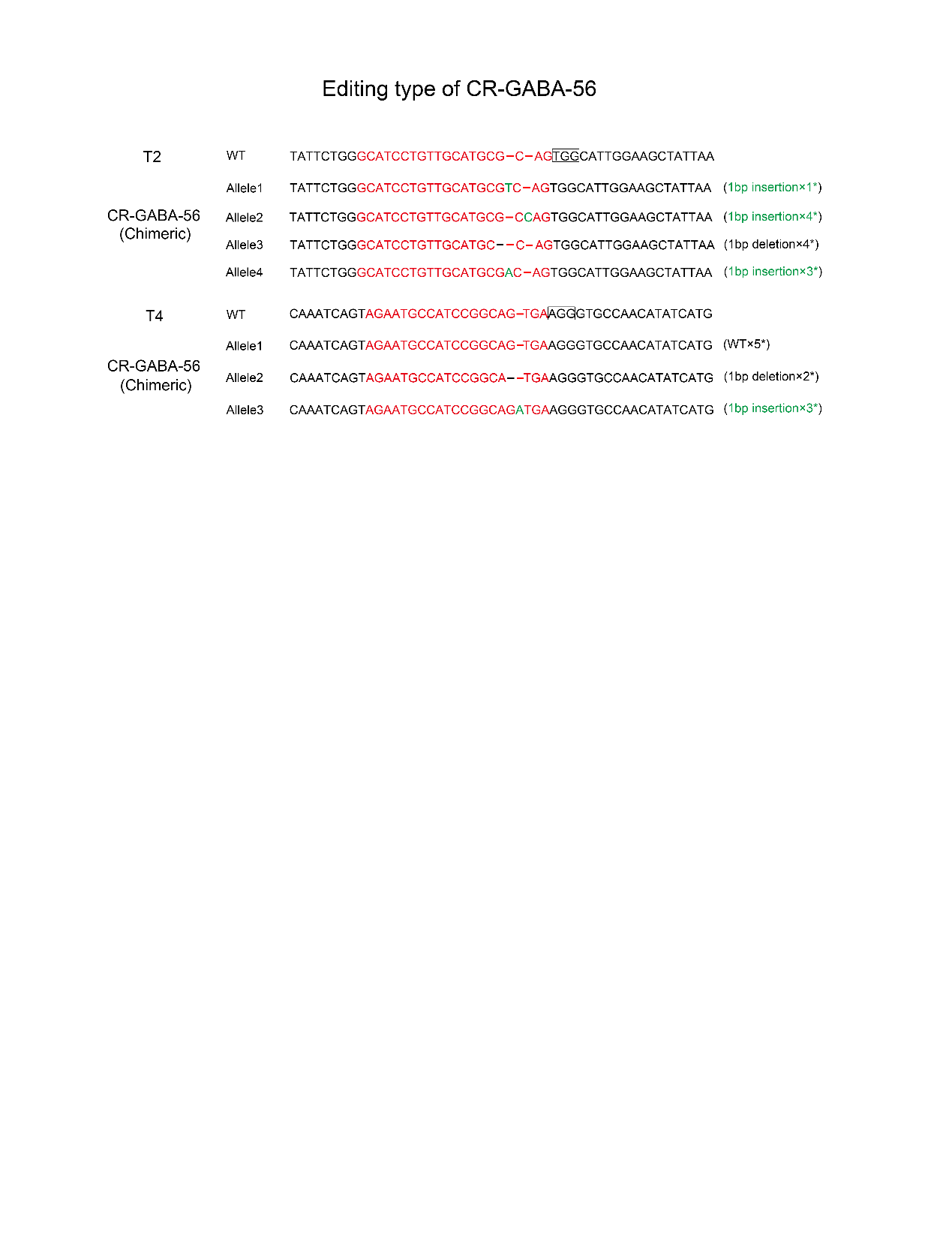
**
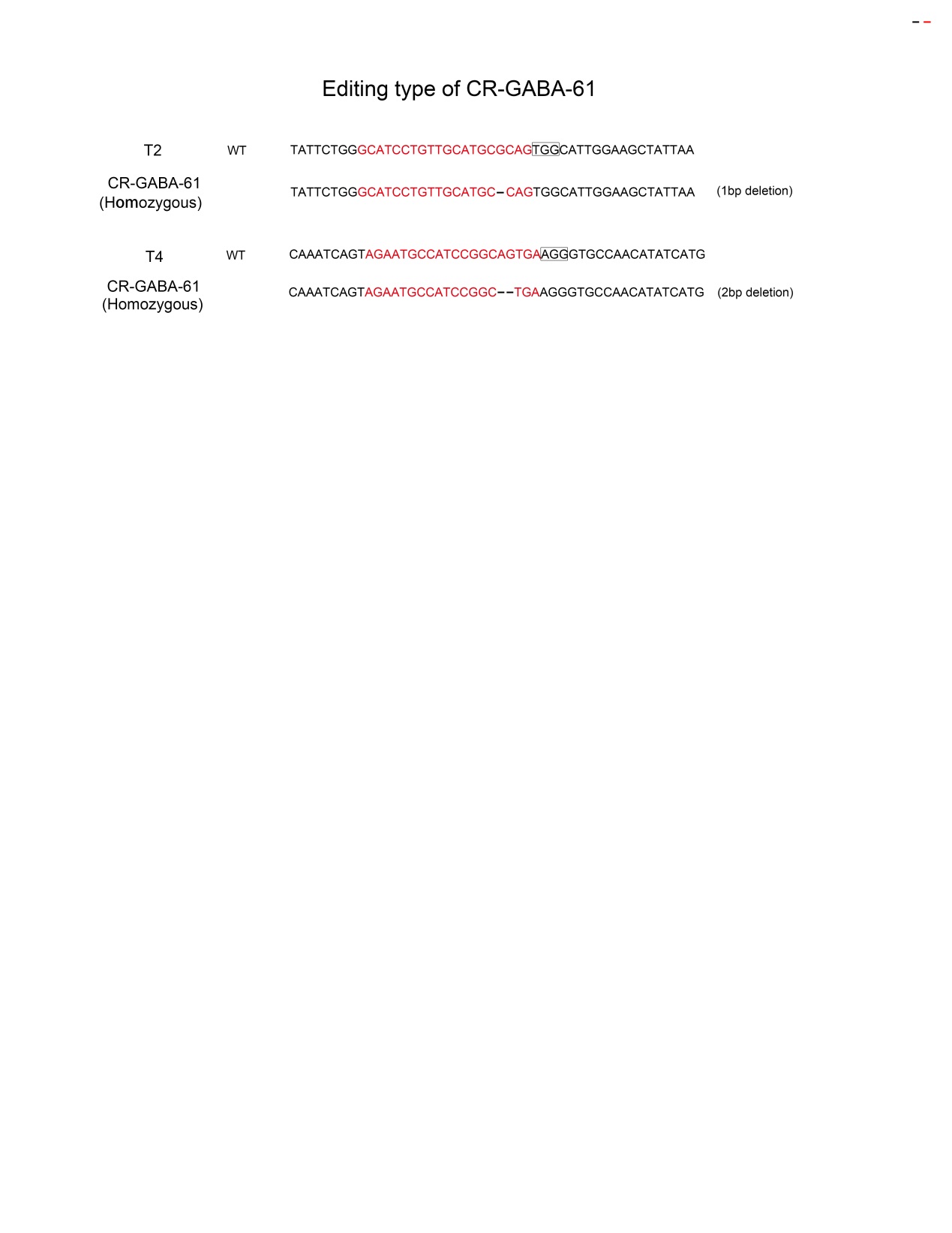

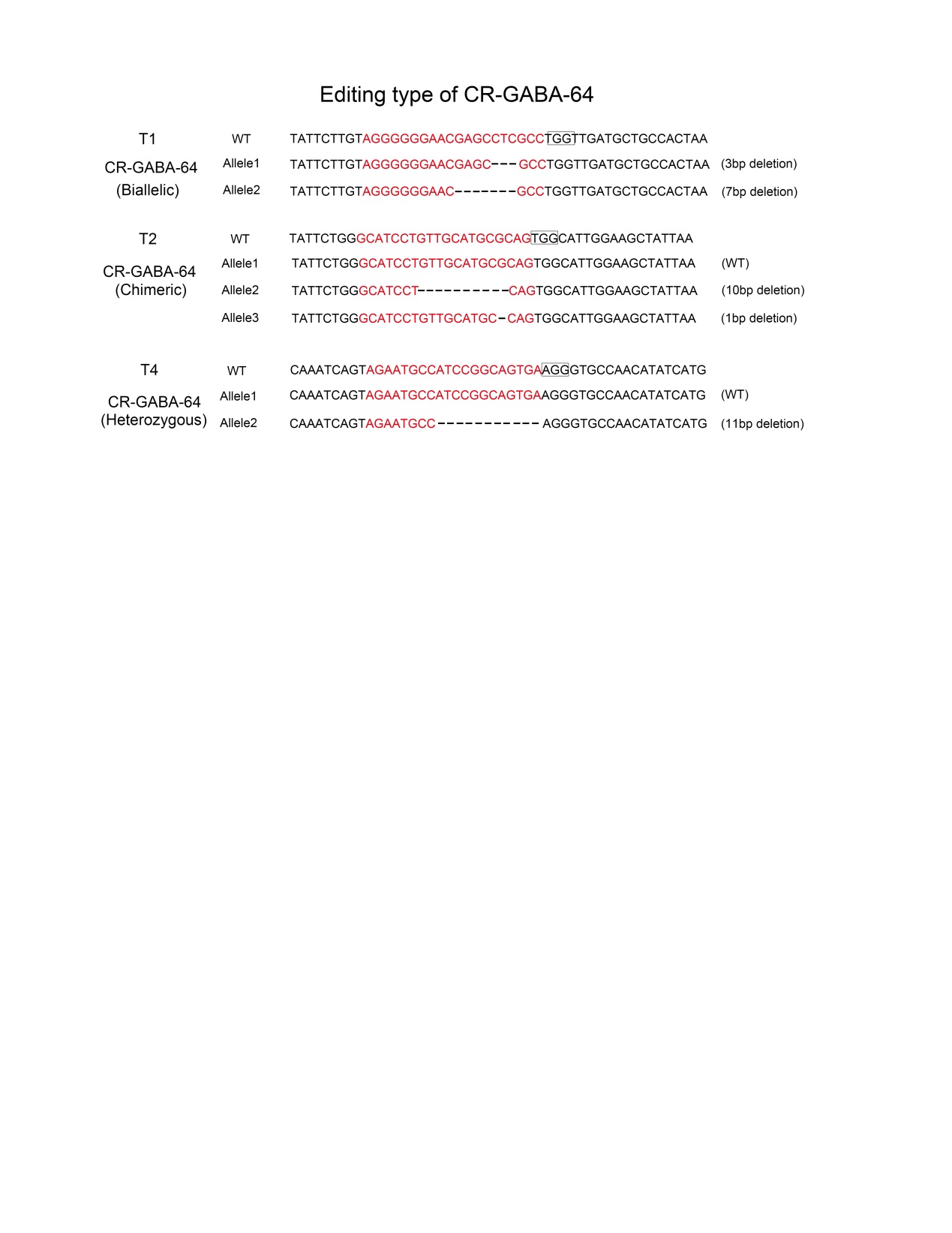

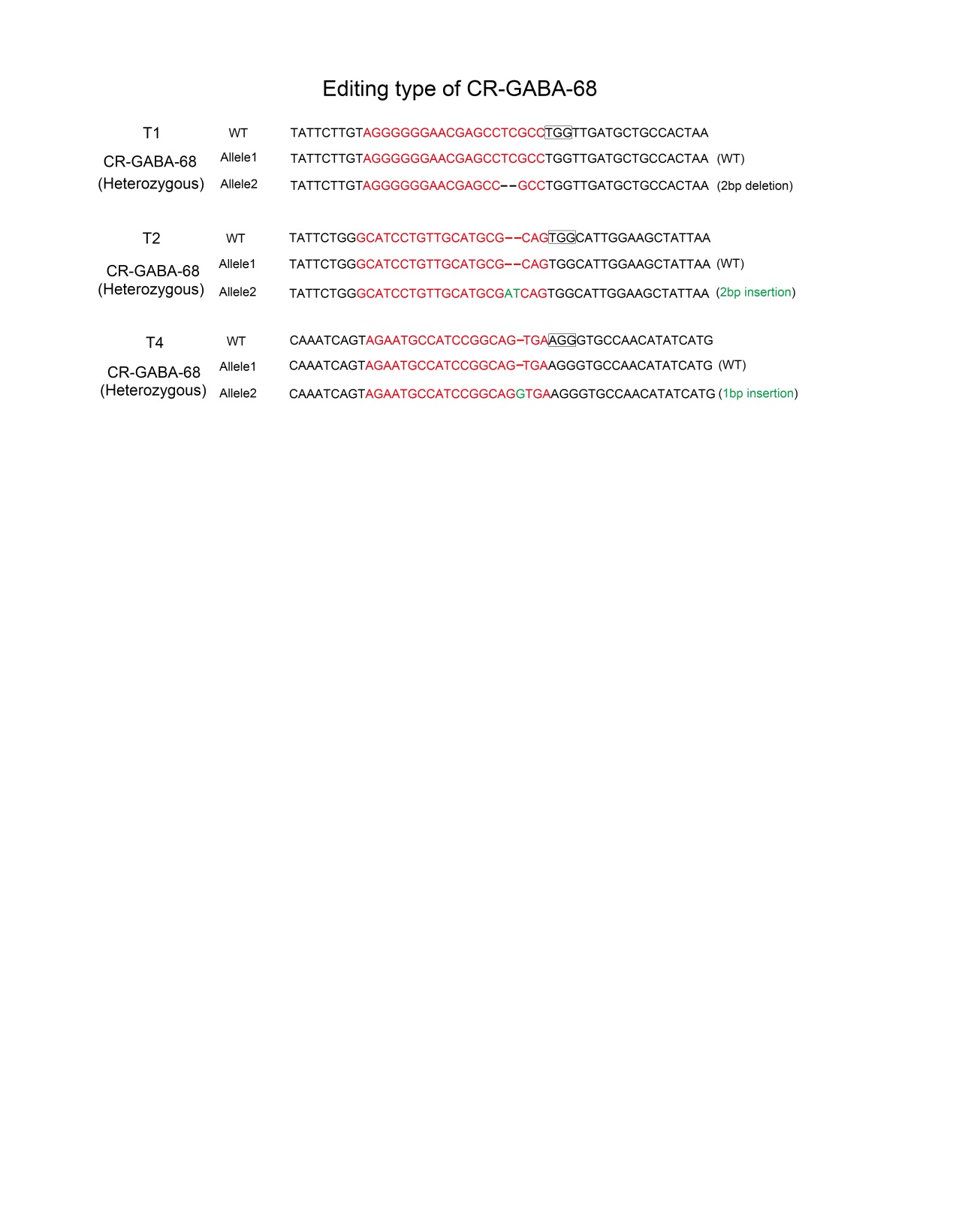

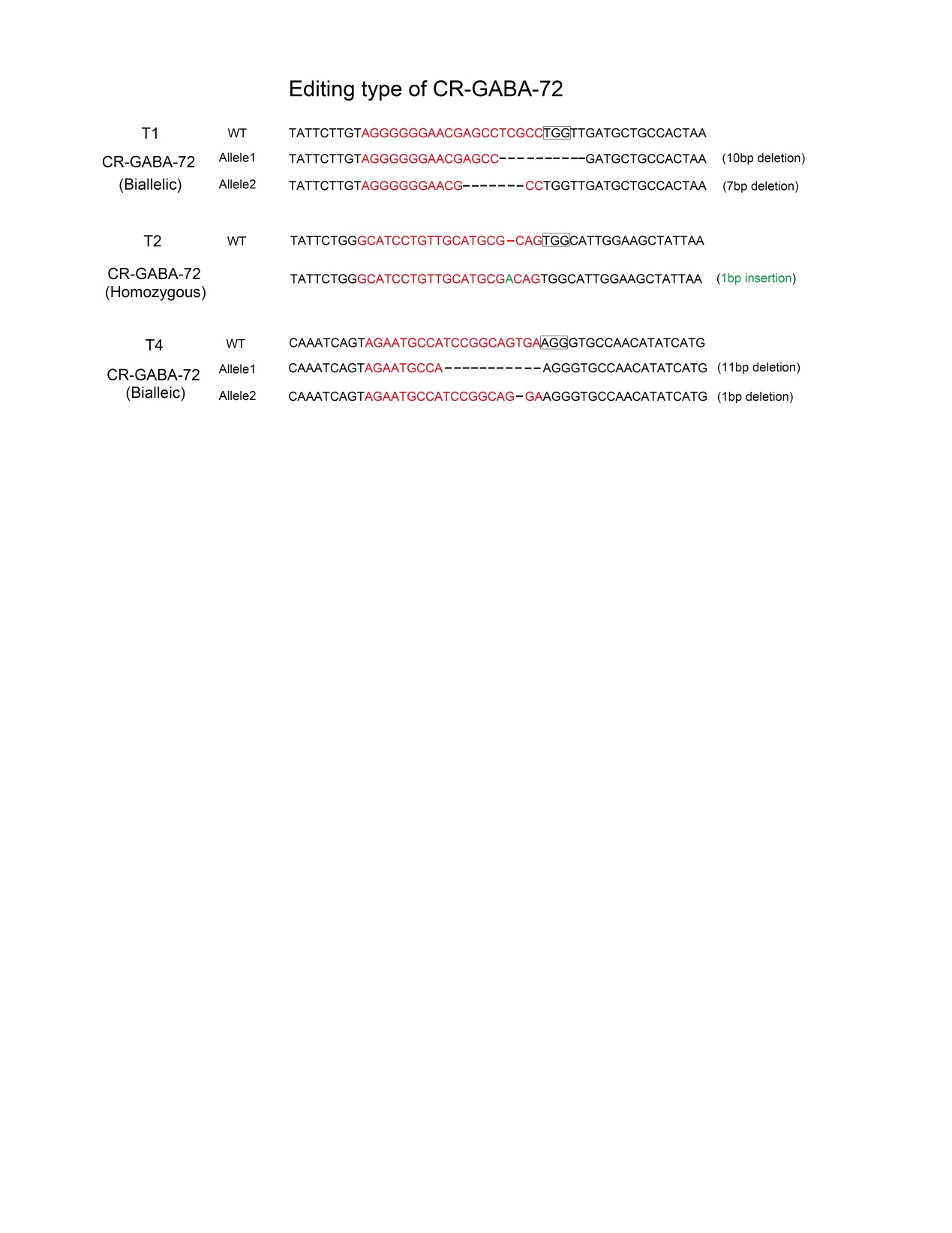

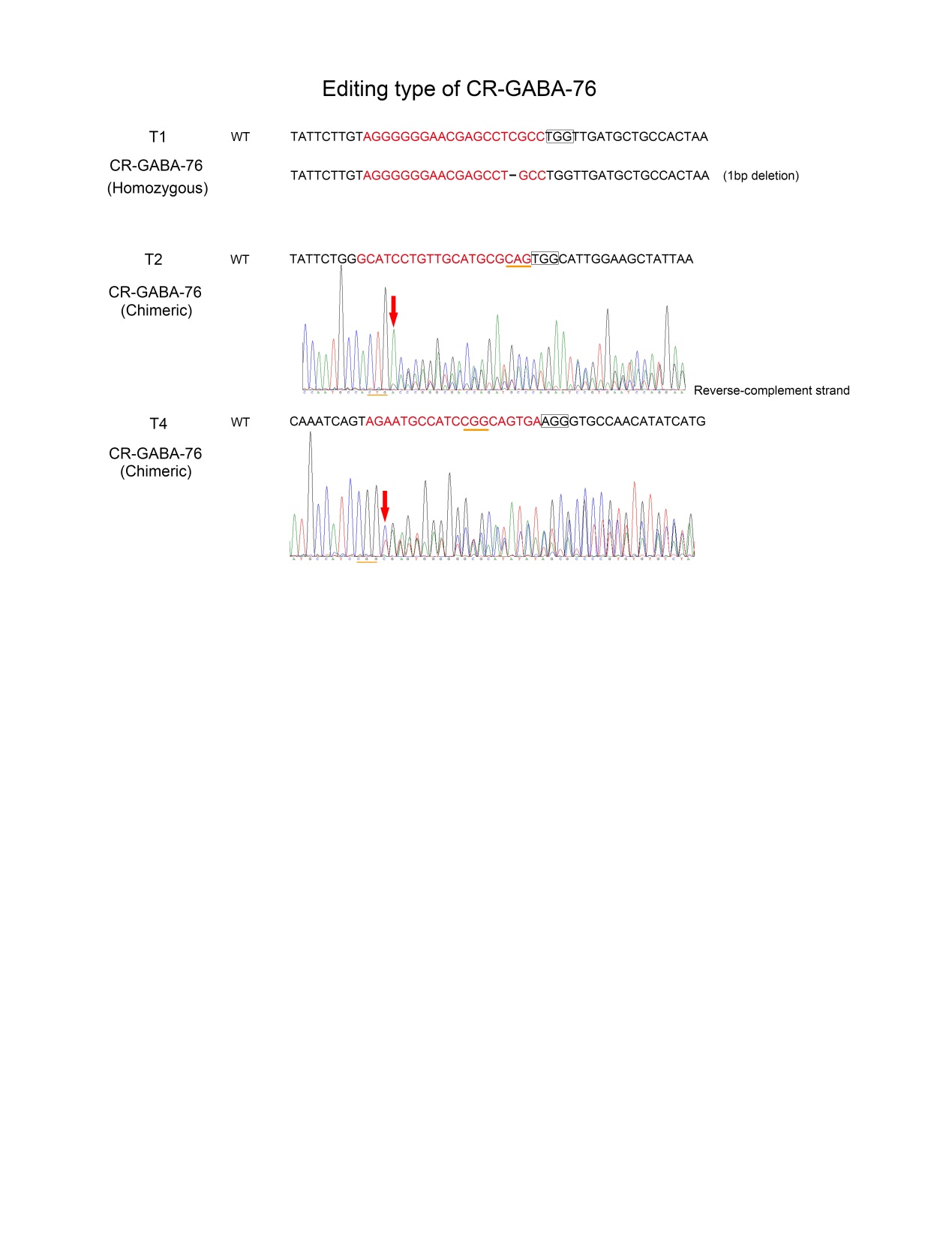

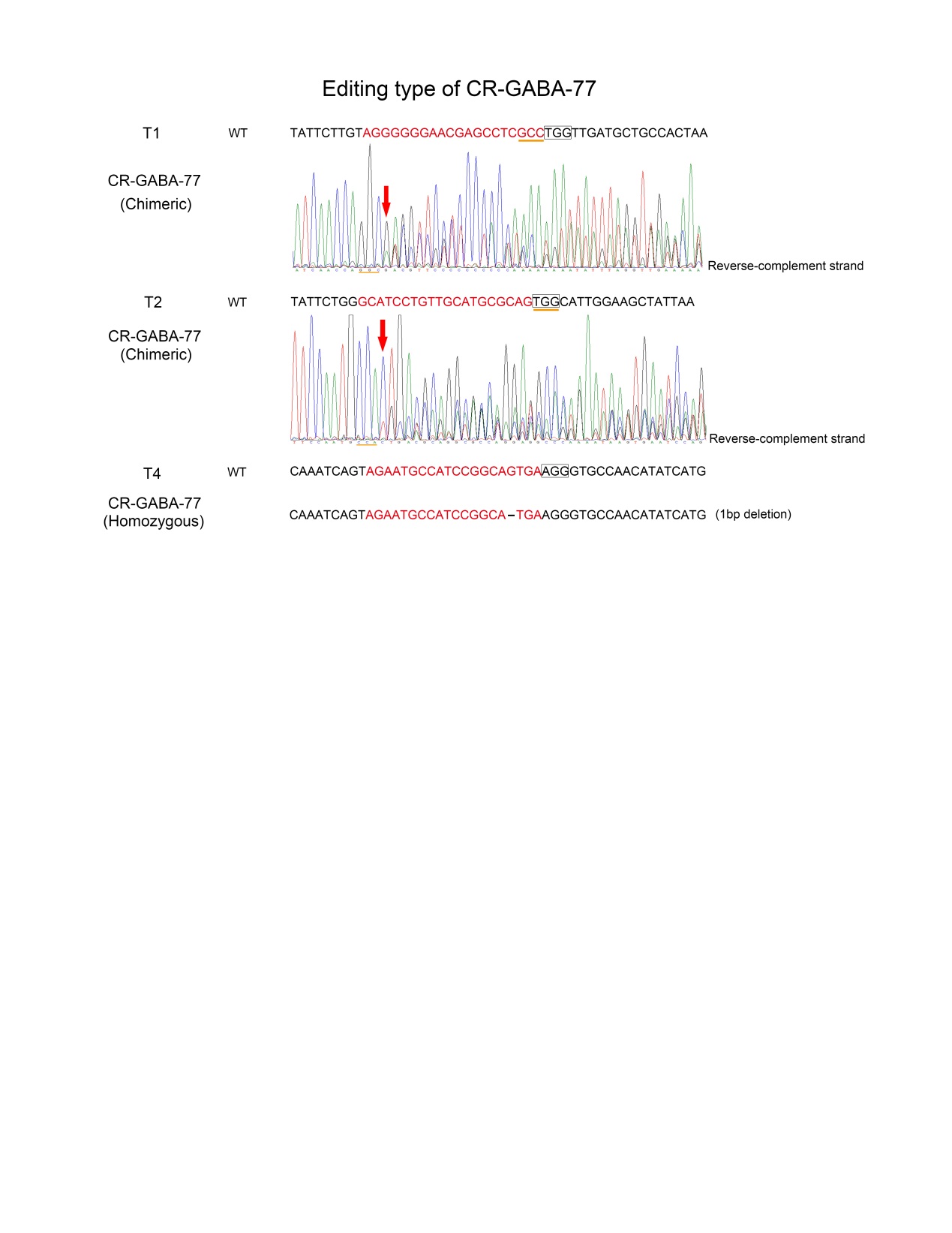

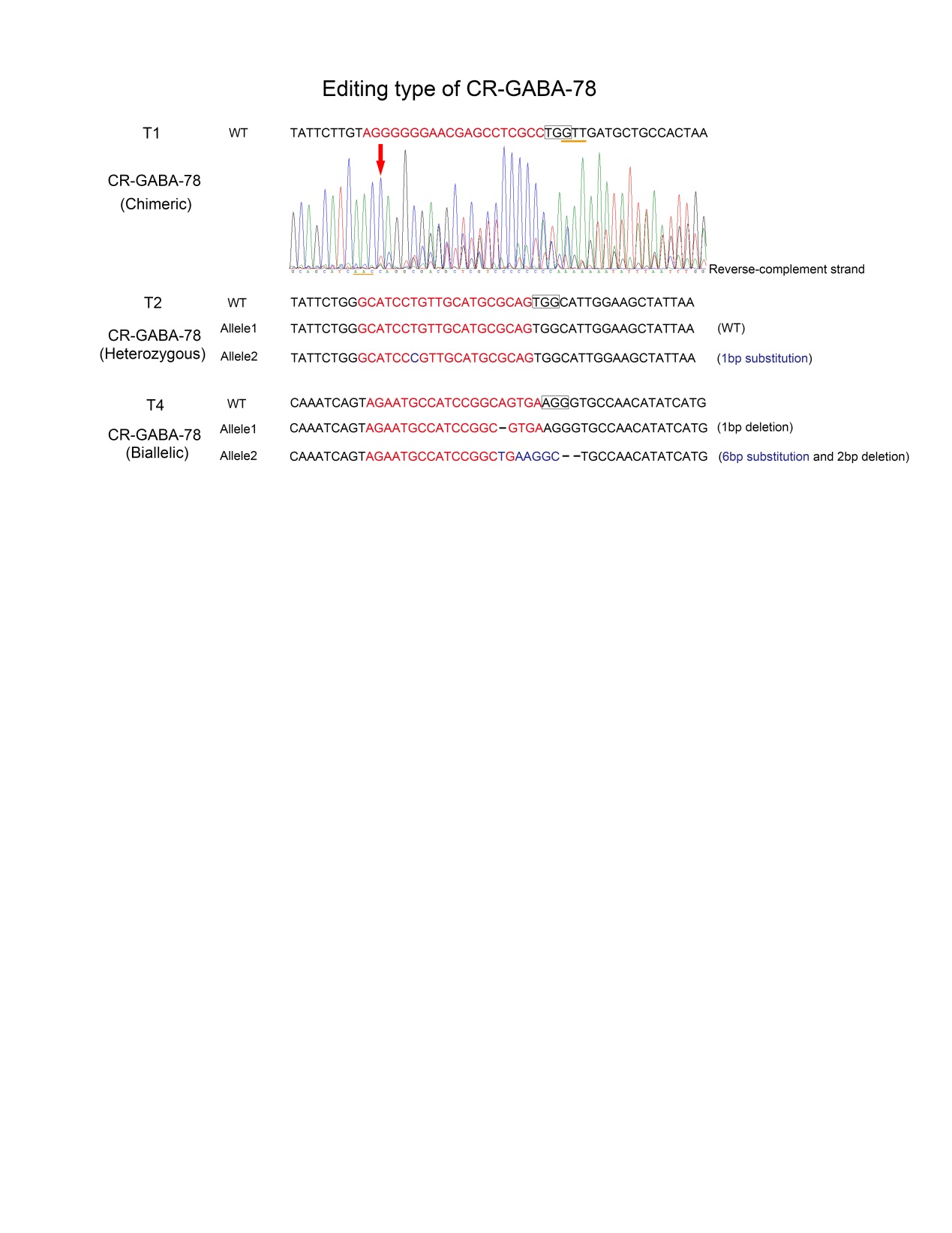

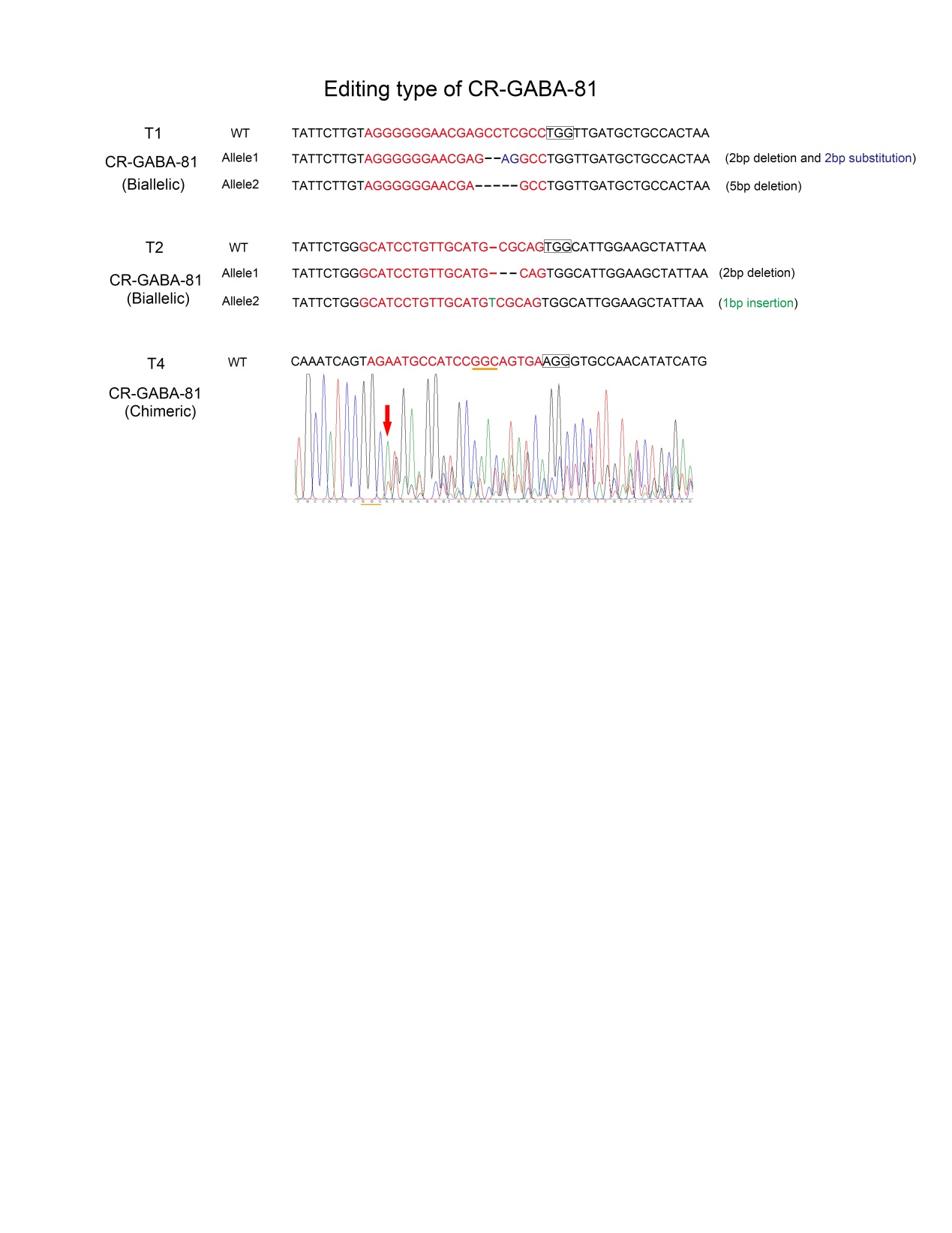

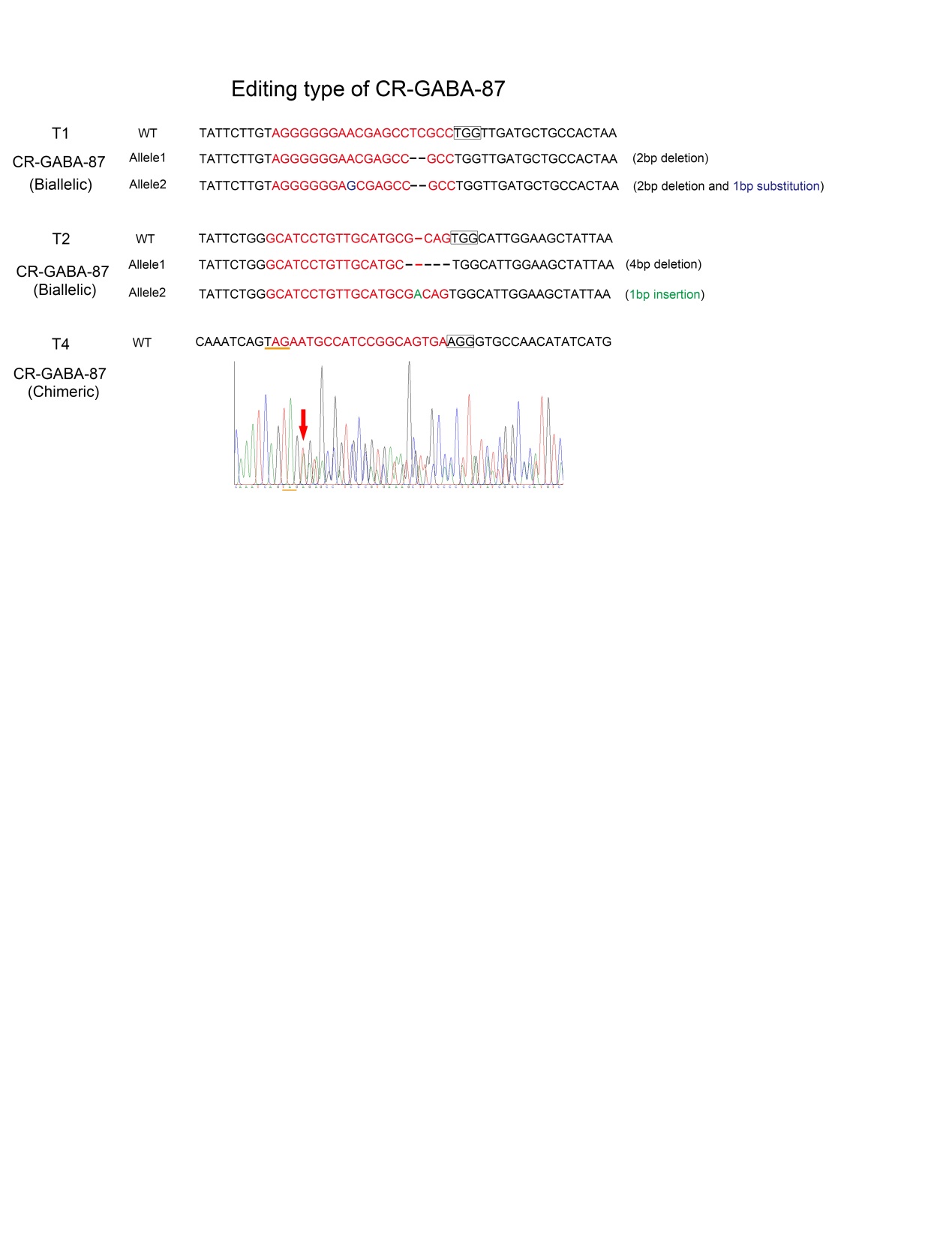
**

**
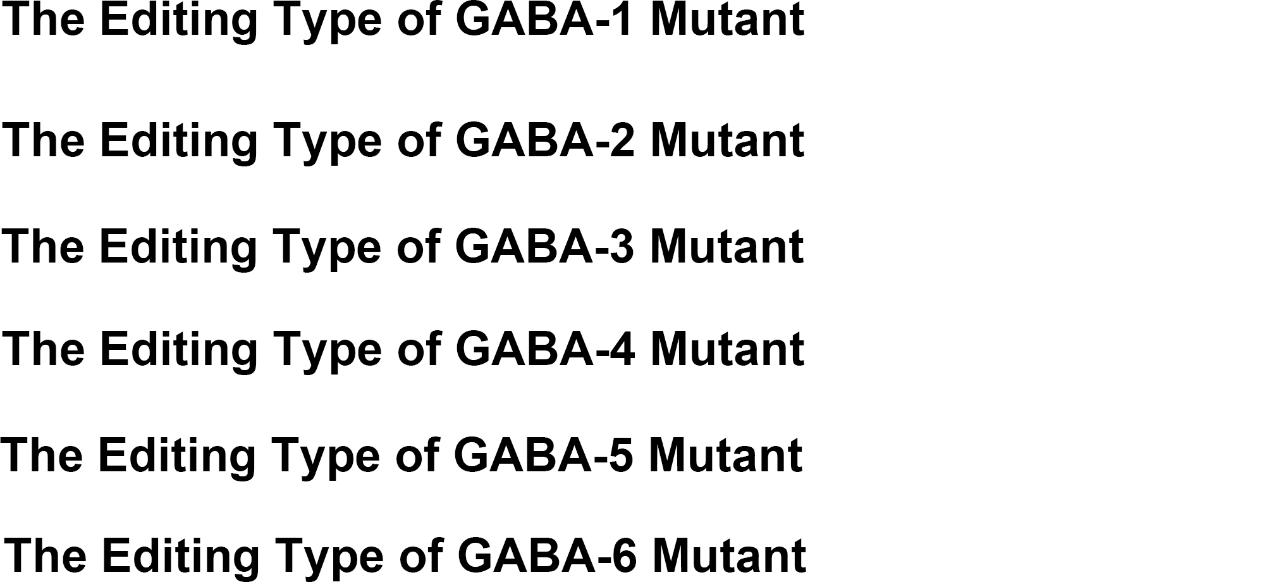

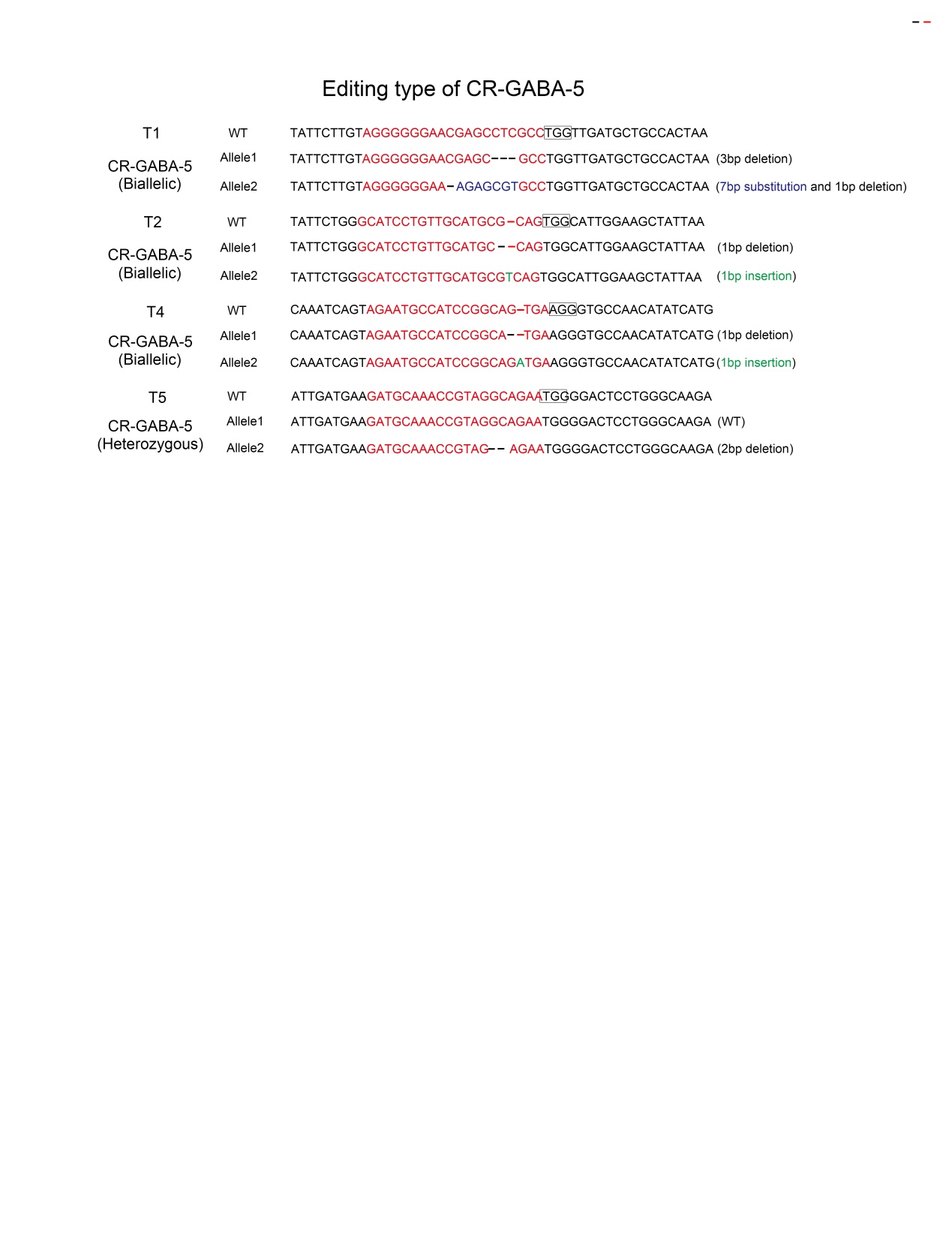
**

**
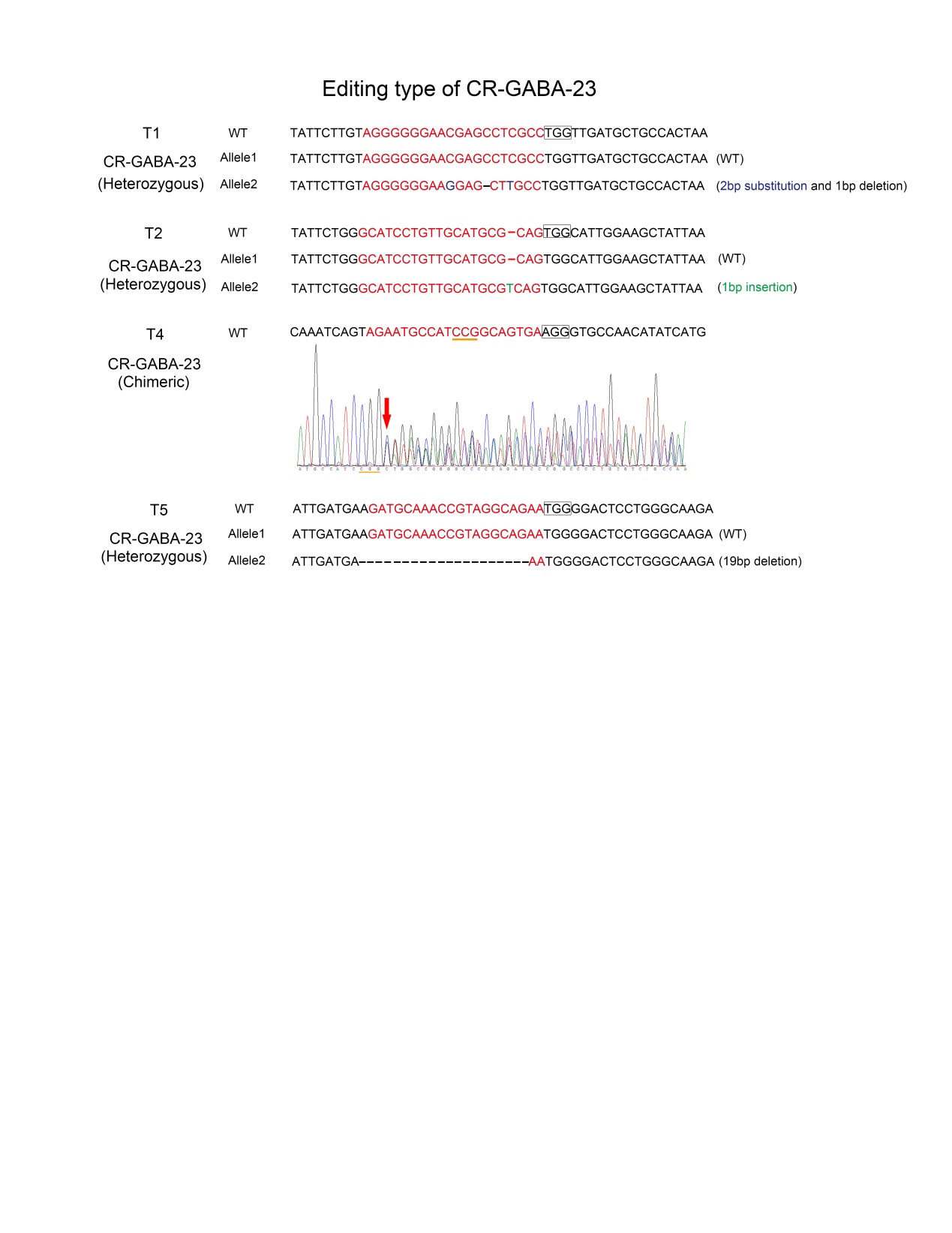

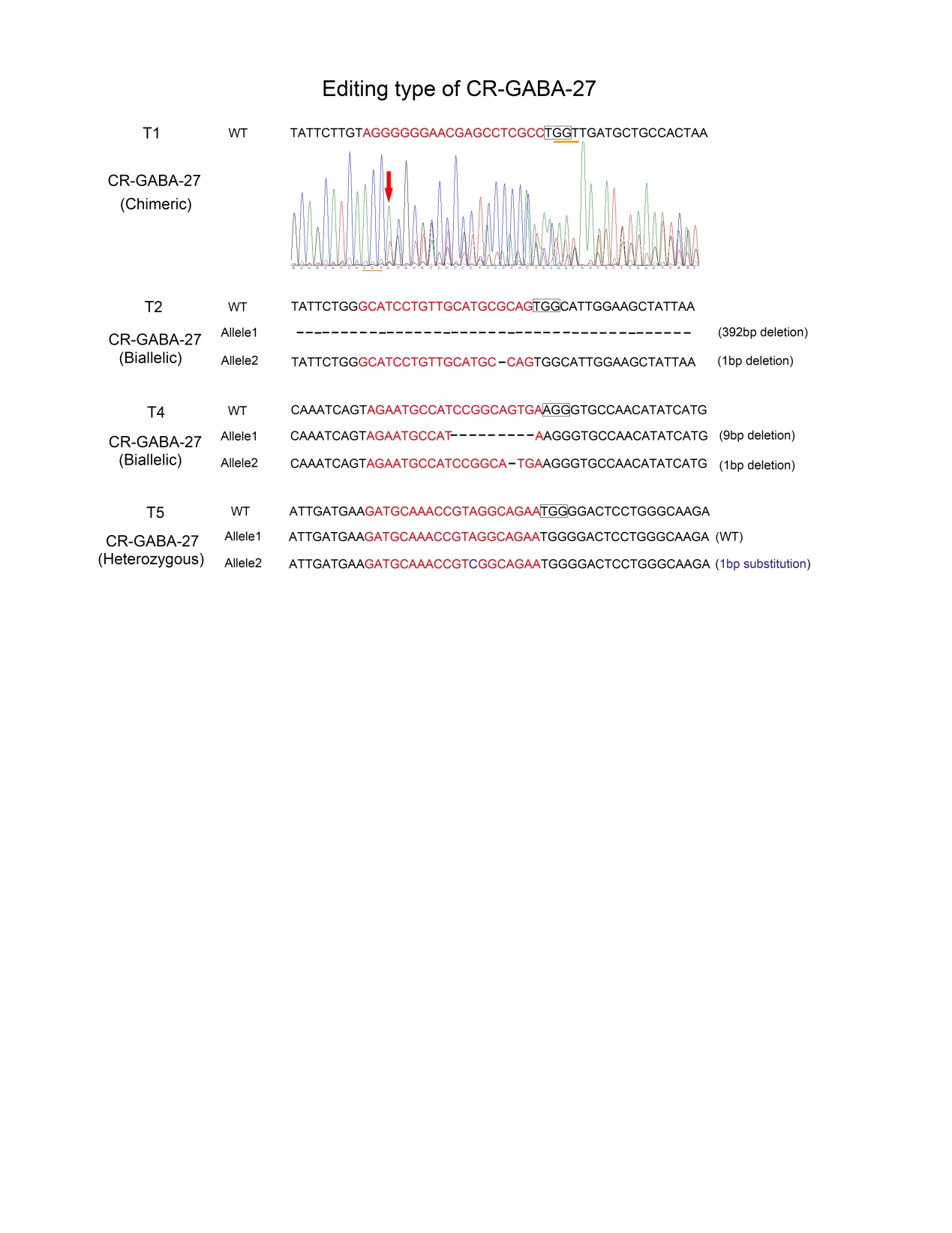

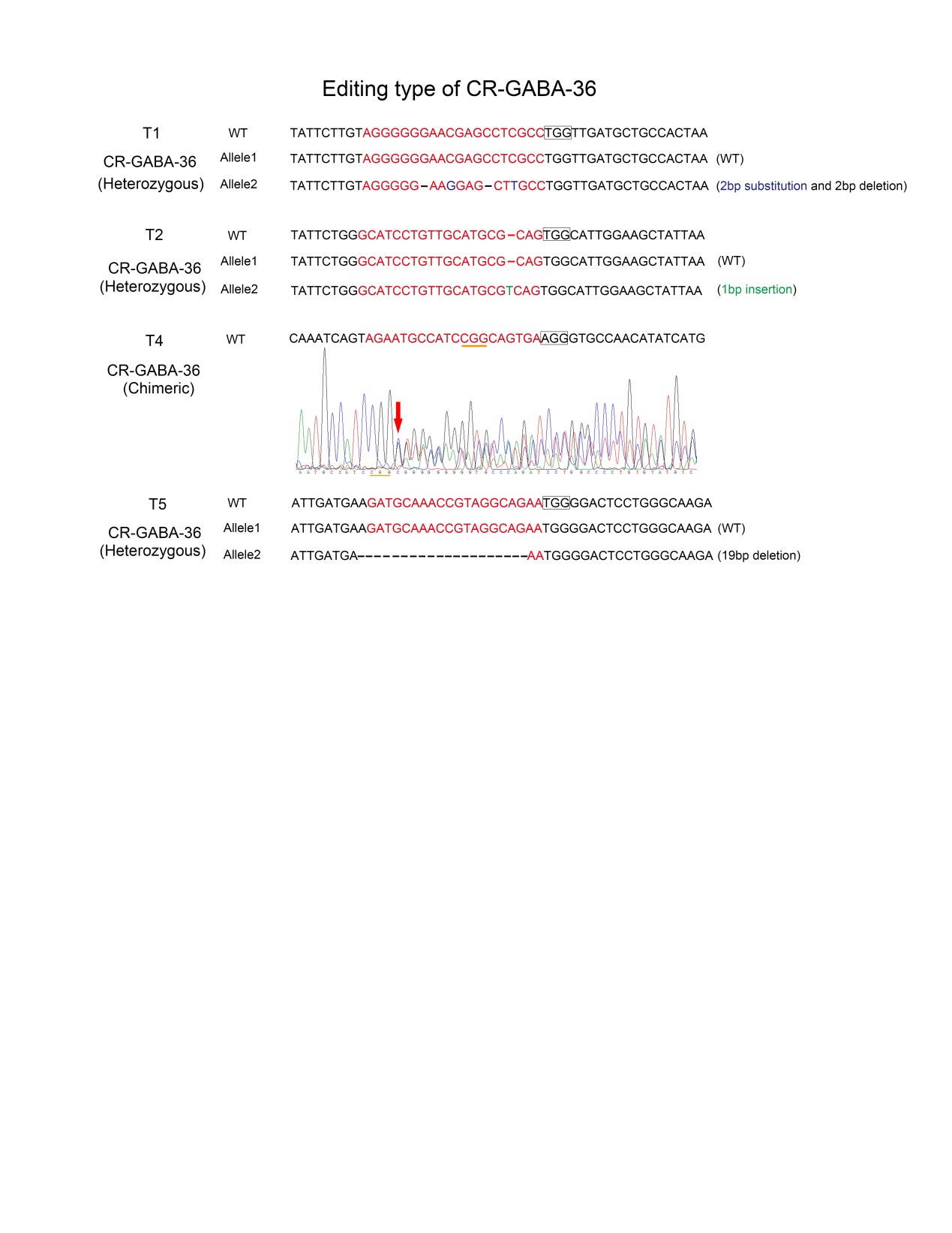

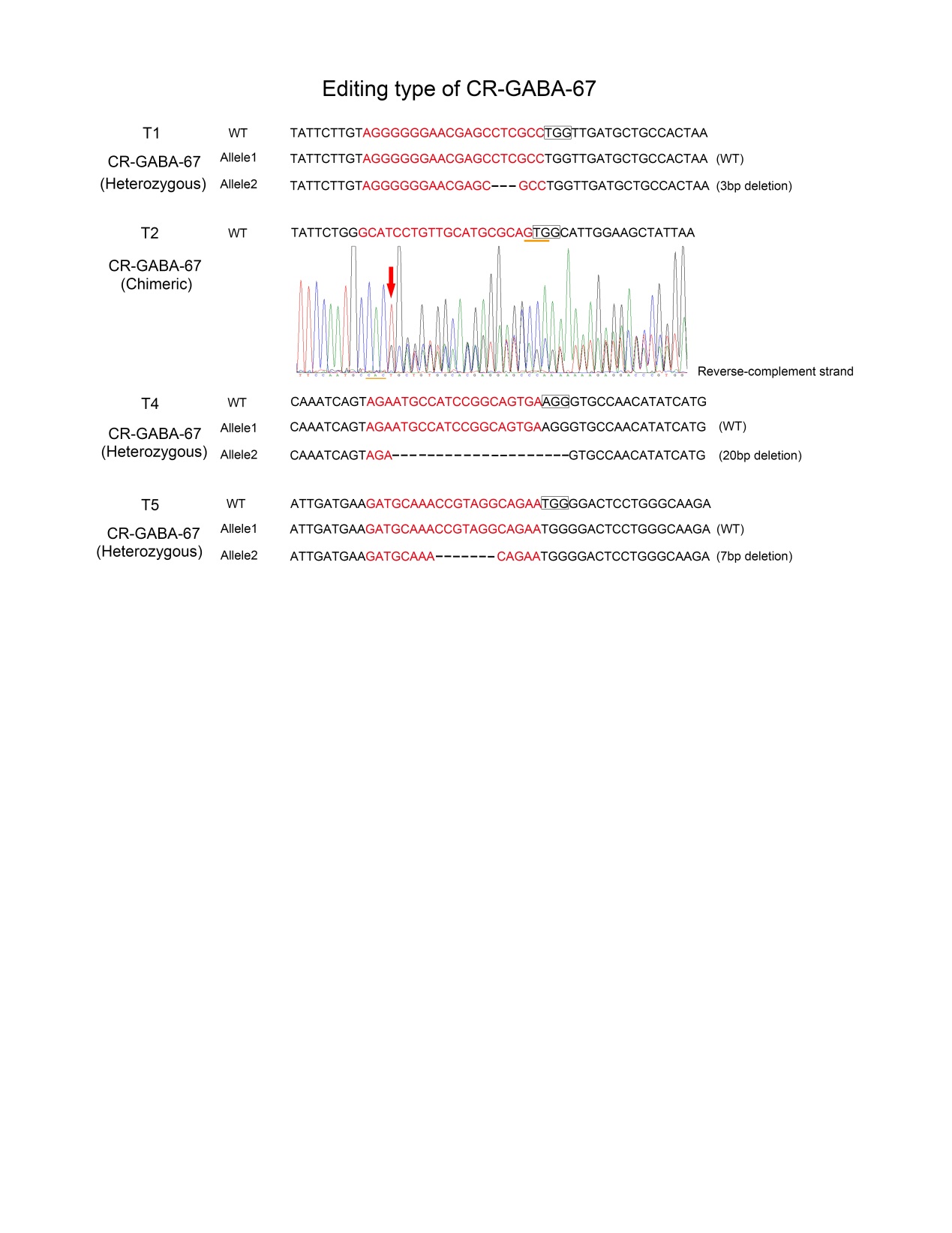
**


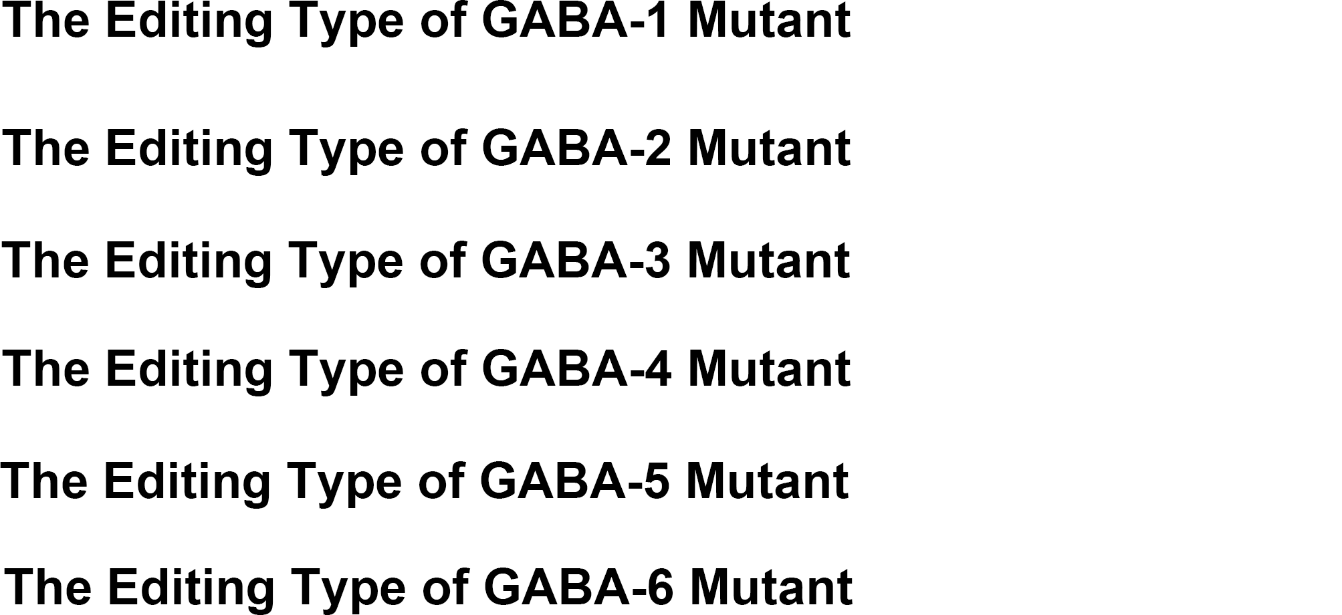
**
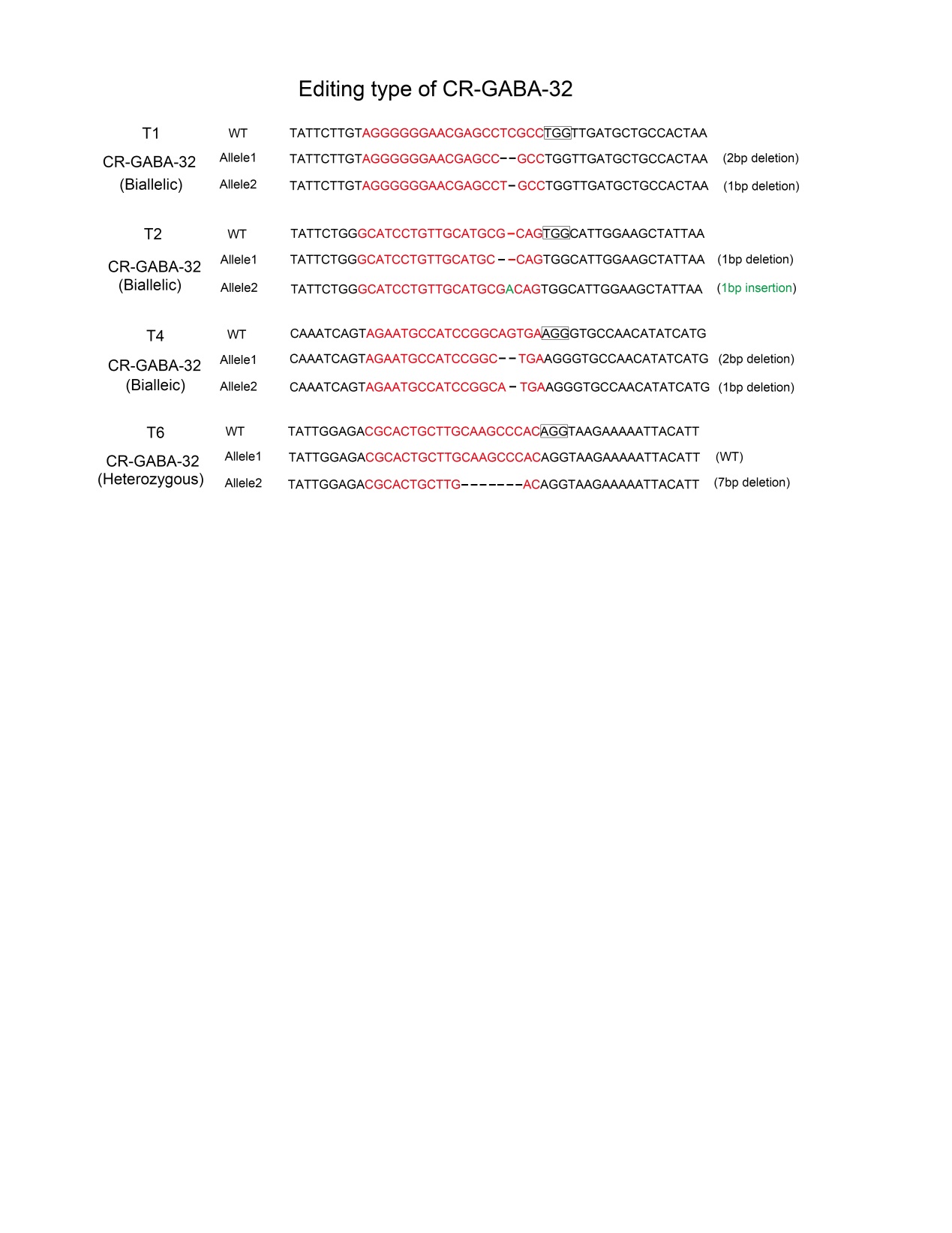
**

**
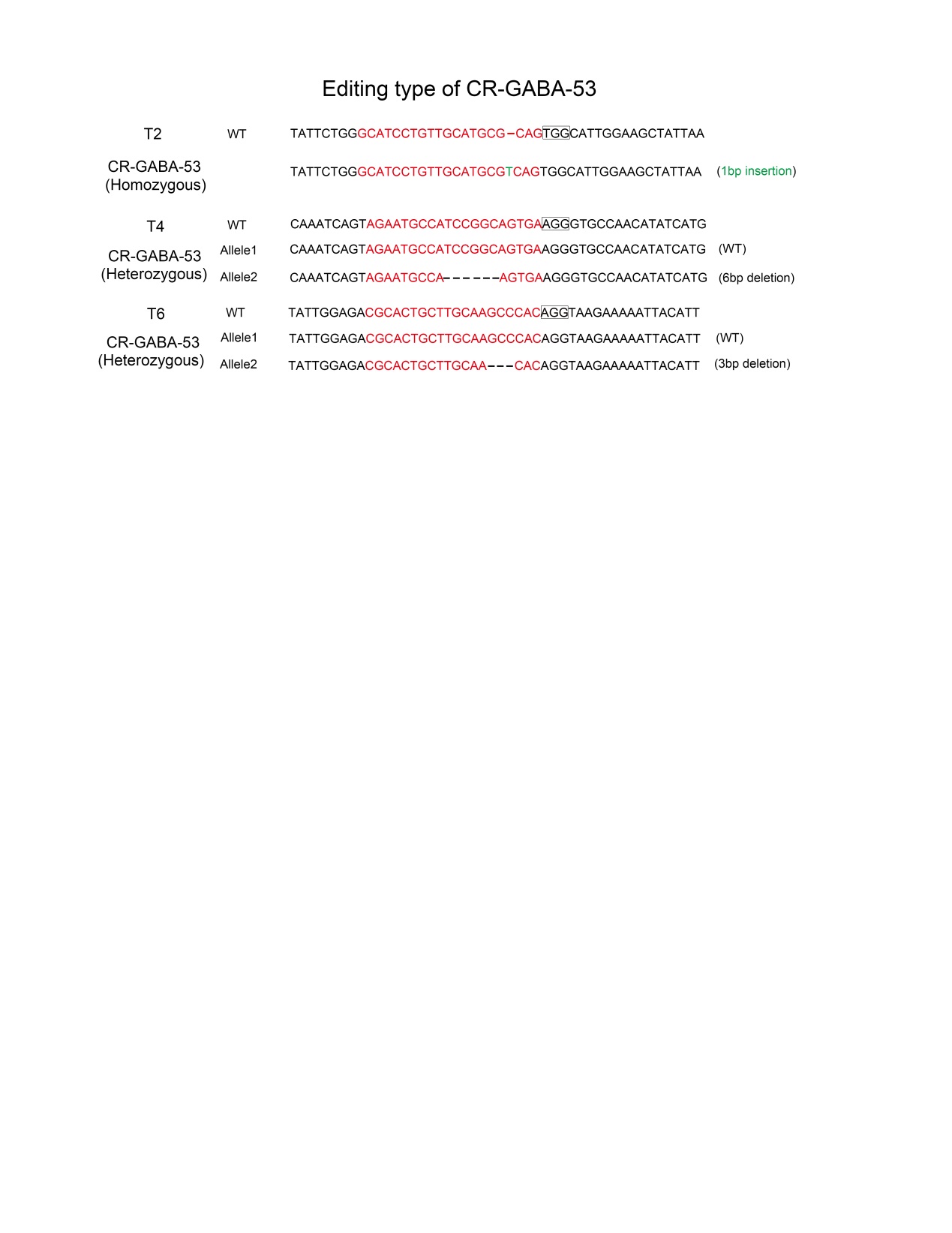

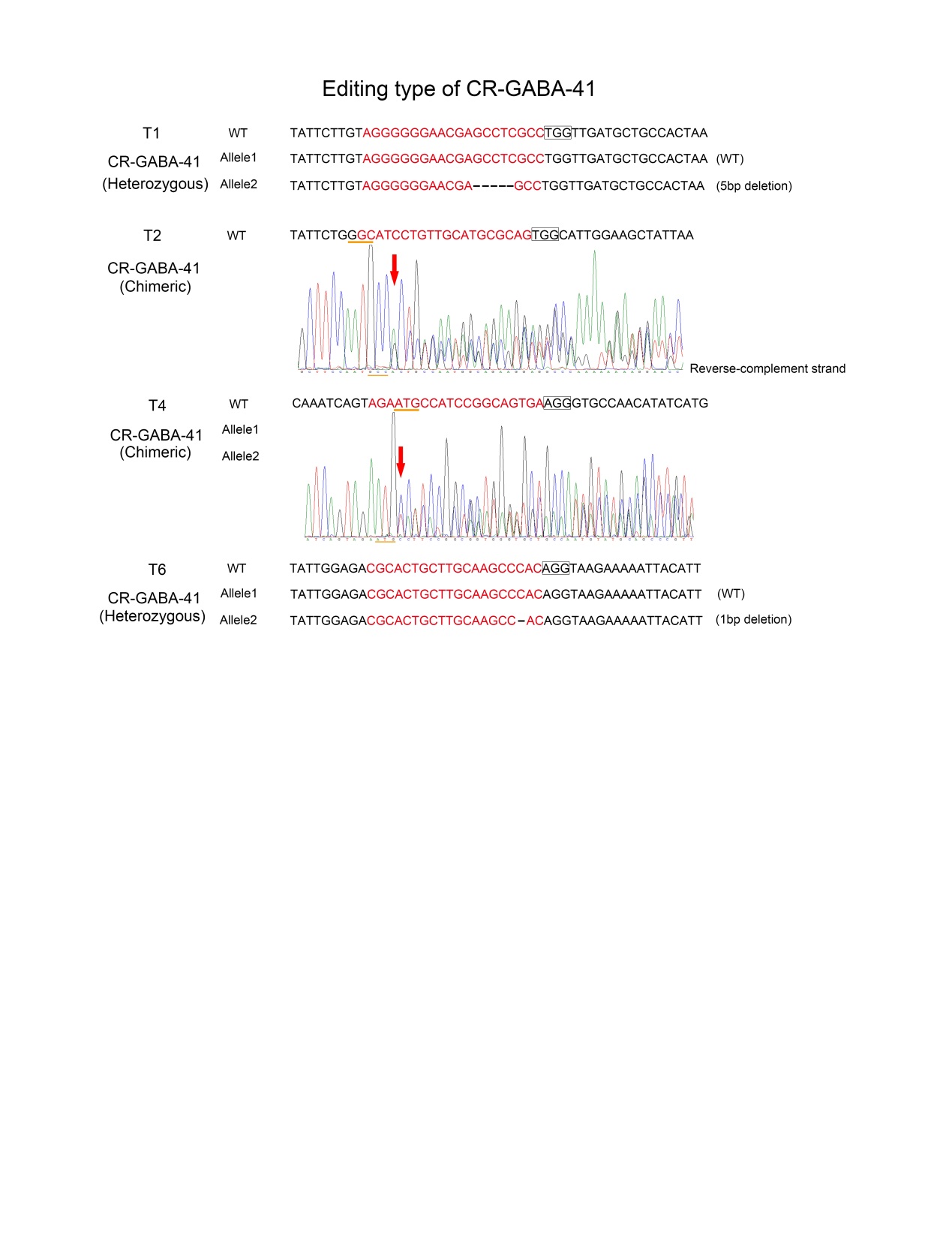

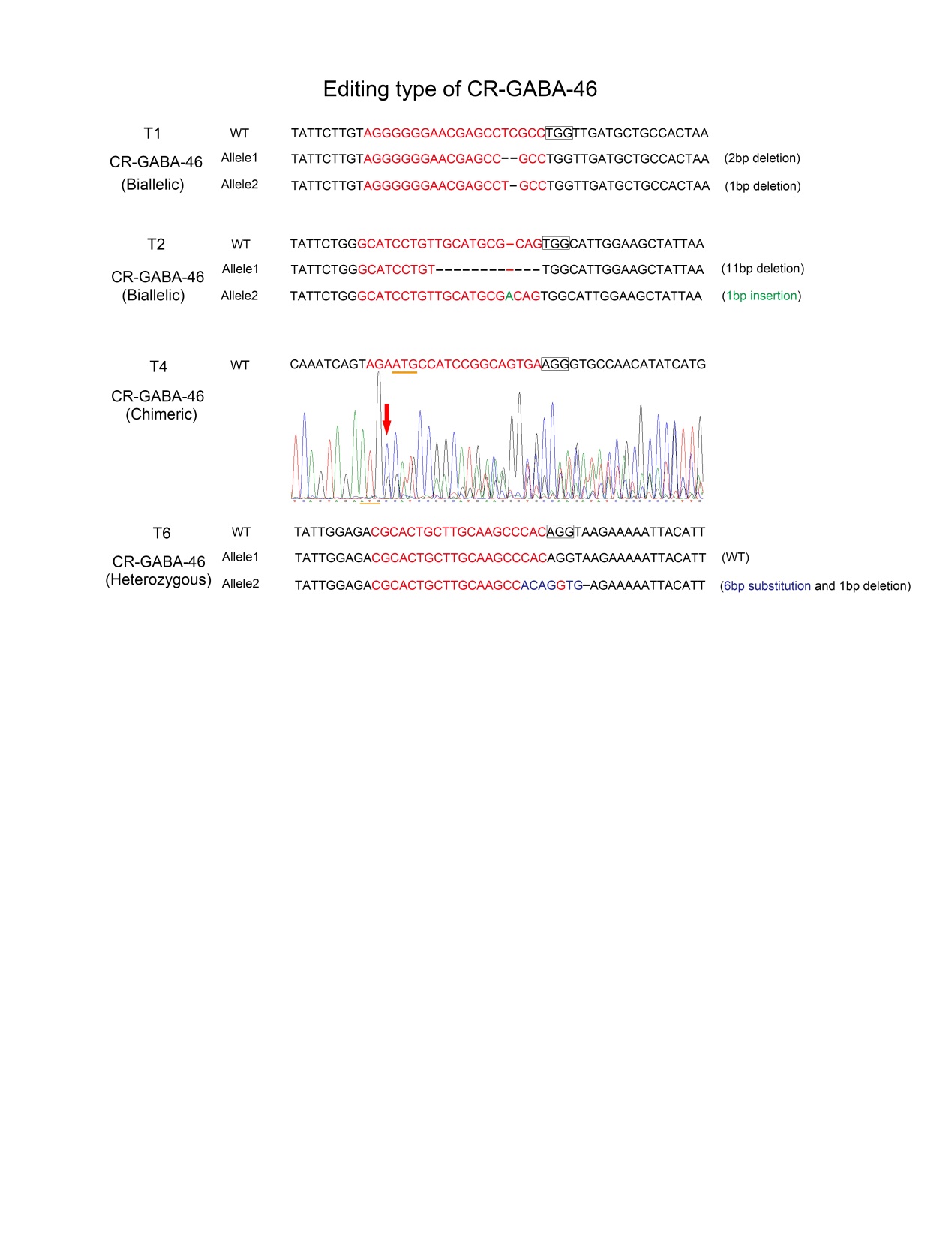

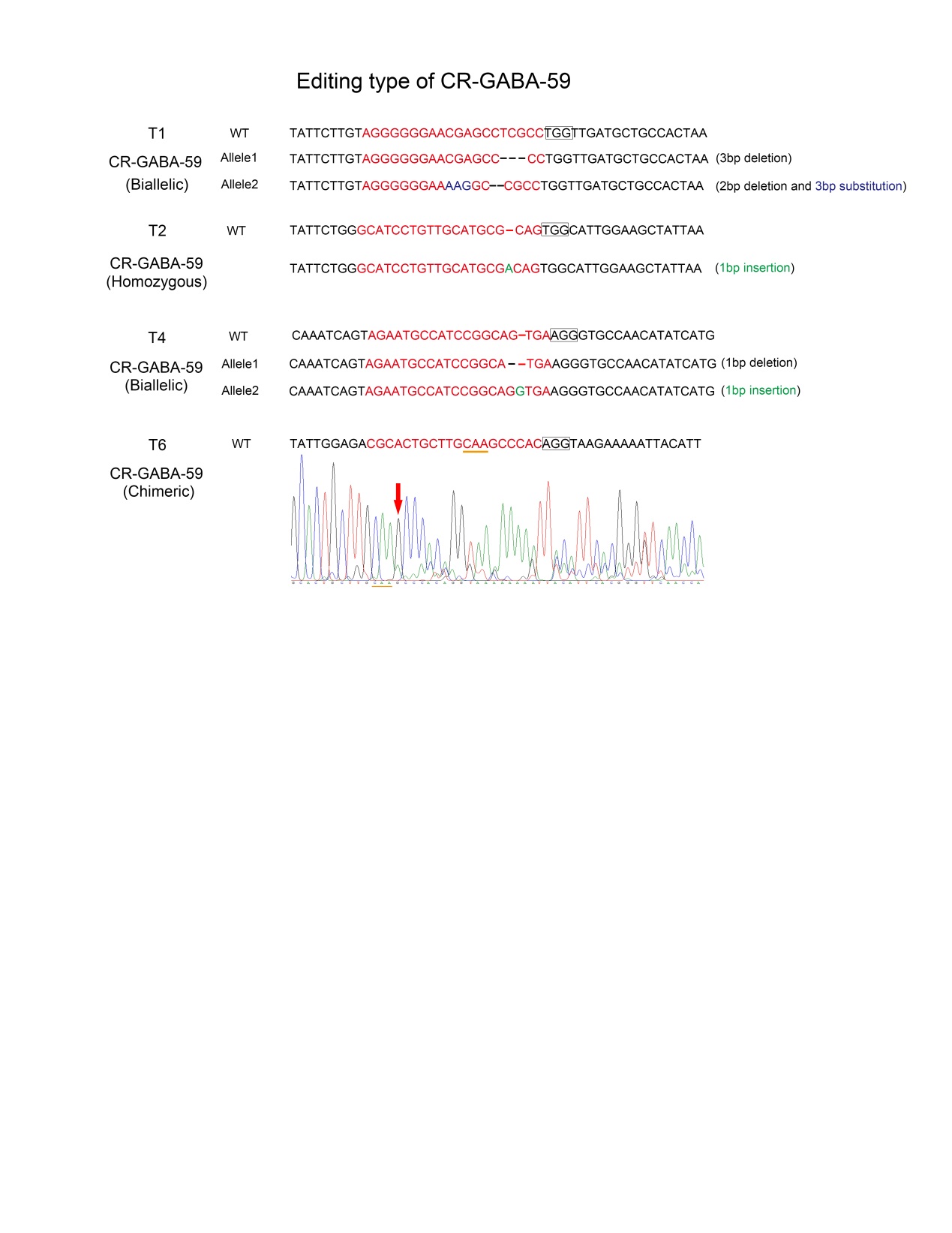

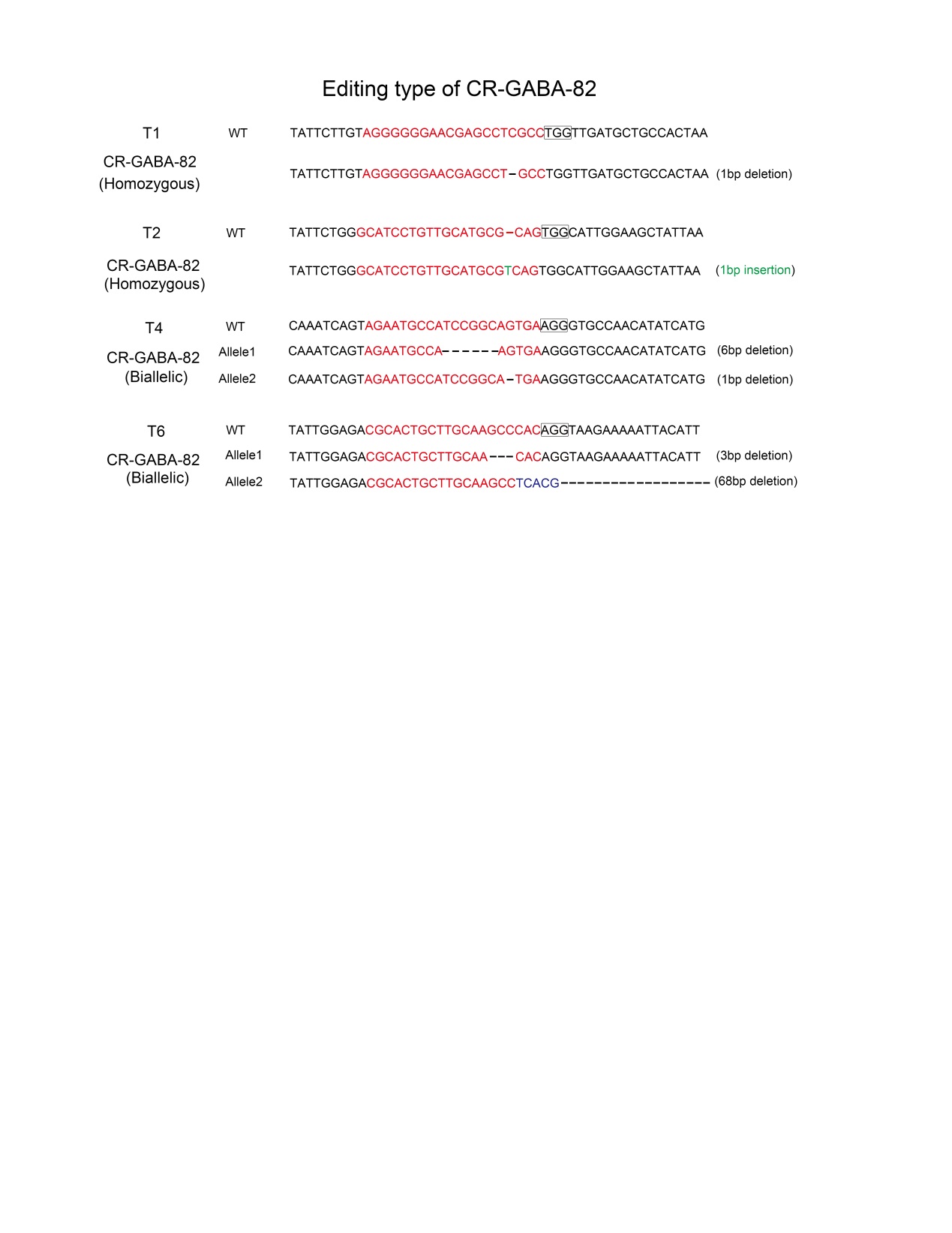

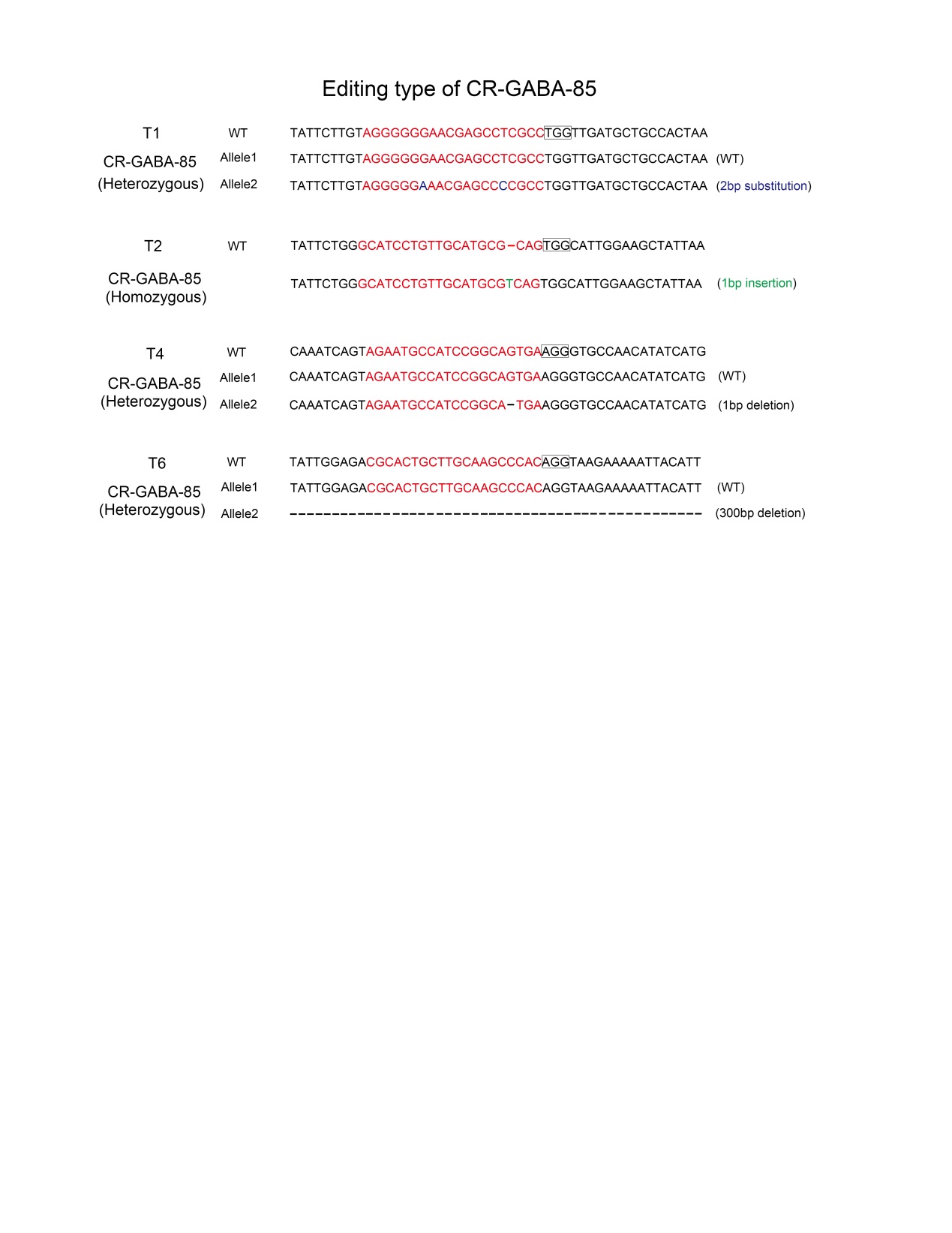
**


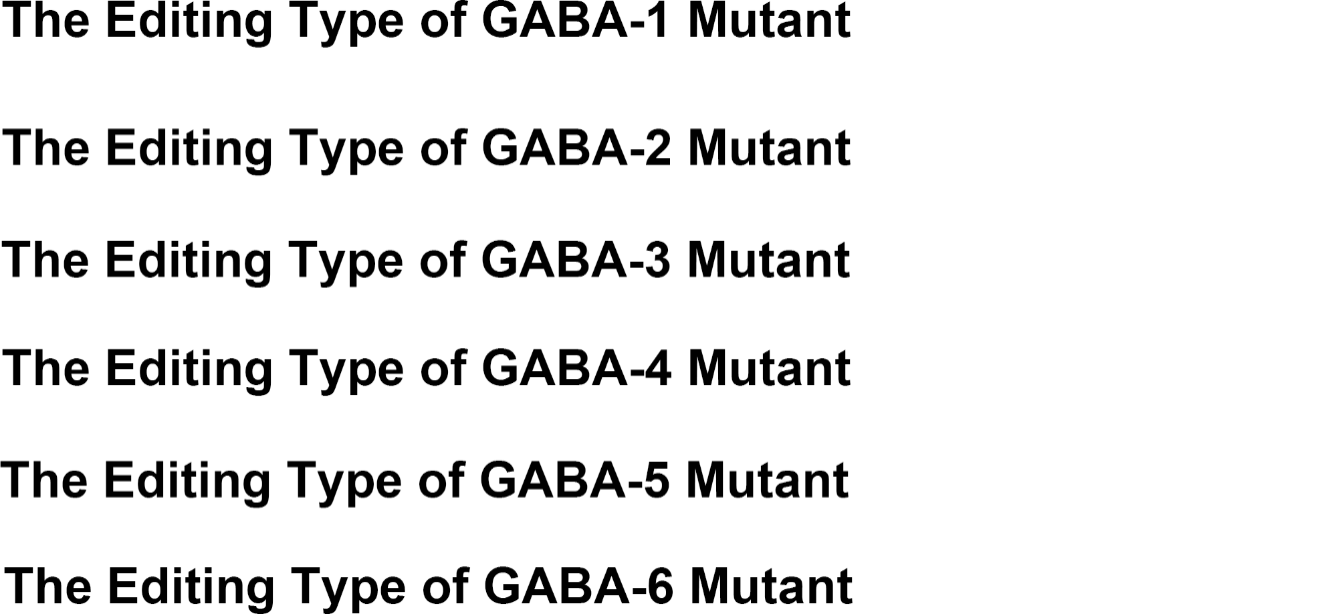
**
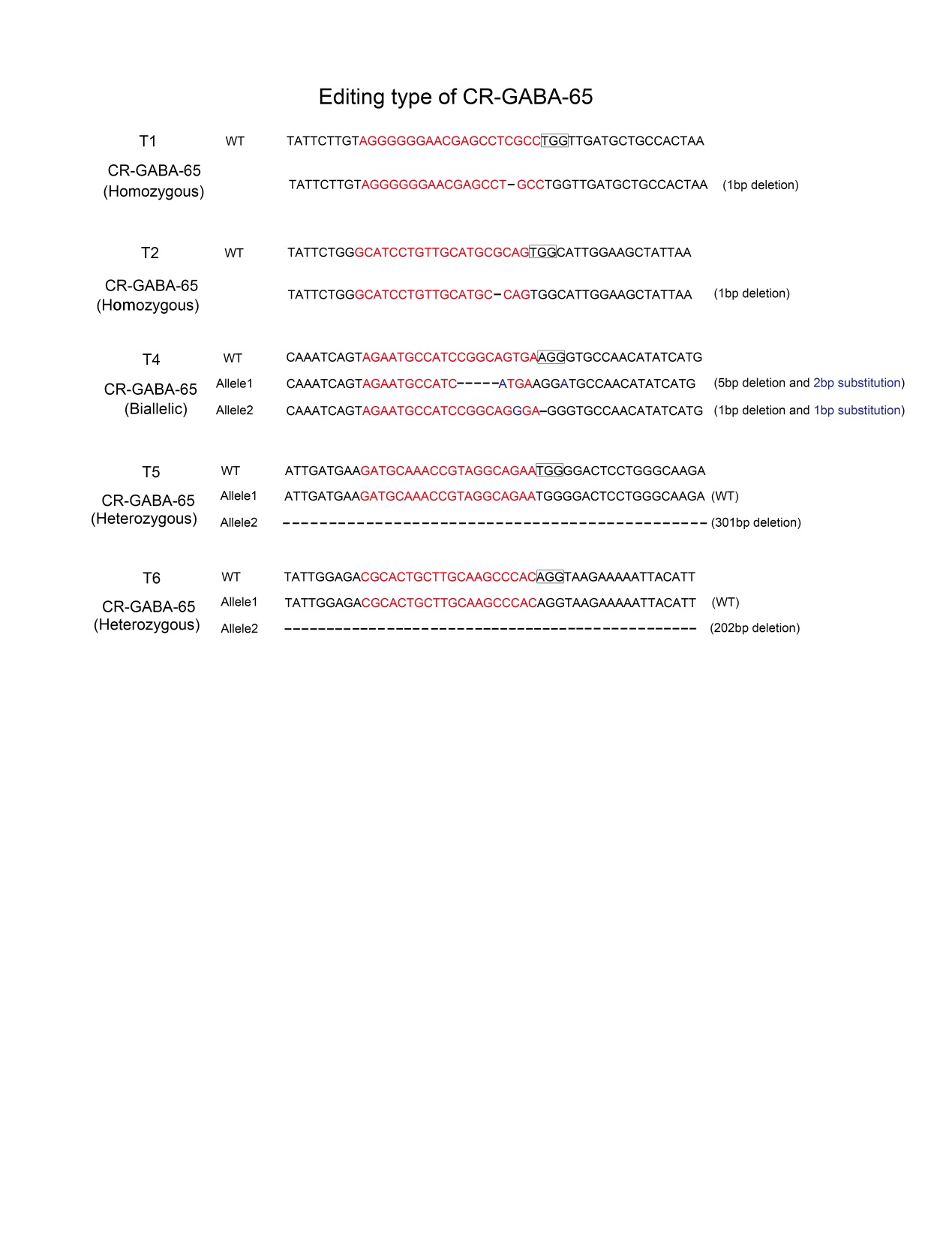
**

**Figure S3. Genome editing type of six kinds of GABA mutants.**

Red letters indicate the target site, minus symbols represent deletions, blue letters represent base substitutions, green letters represent base insertion. Chimeric mutations were shown with sequencing chromatogram. The letters with yellow underline were with single sequencing peak. The red arrow indicated the beginning of chimeric mutation with multi sequencing peak. Target regions in plants CR-GABA-20 and CR-GABA-56 were amplified and cloned into a TA-cloning vector. Each clone was sequenced. *, number of independent clones with a same sequence.


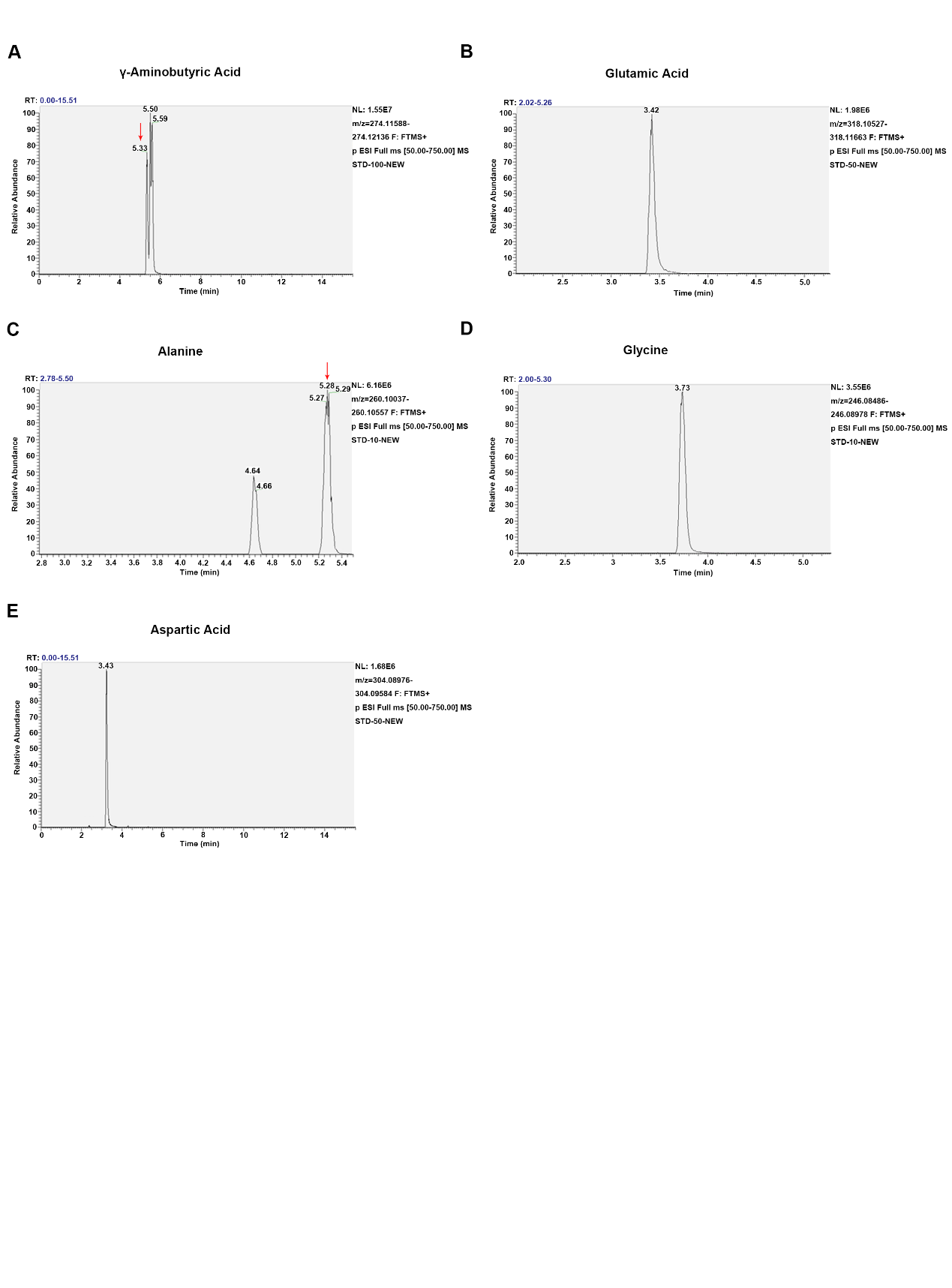


**Figure S4.** **HPLC-MS** **Chromatogram of GABA and GABA metabolism associated amino acids.**

(A) HPLC-MS Chromatogram of GABA. Because amino mixture standard was used in HPLC-MS analysis, α-aminobutyric acid, β-aminobutyric acid and GABA were shown in chromatogram and GABA was indicated by red arrow. (B) HPLC-MS Chromatogram of glutamic acid. (C) HPLC-MS Chromatogram of alanine. Red arrow indicates the chromatogram of alanine. (D) HPLC-MS Chromatogram of glycine. (E) HPLC-MS Chromatogram of aspartic acid.


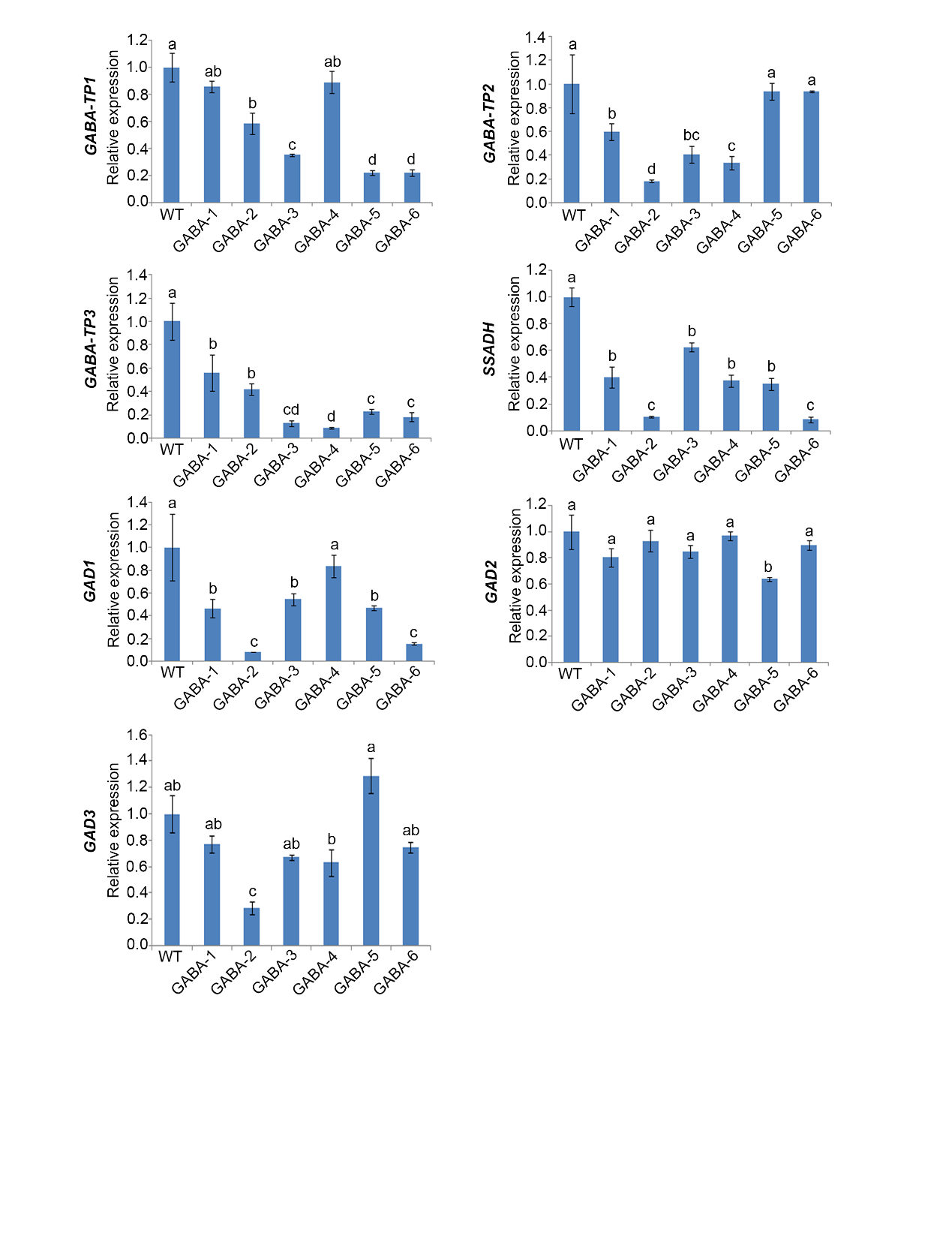


**Figure S5. qRT-PCR analysis of GABA shunt related genes in the leaves of WT and GABA mutants.**

Error bars representing standard deviation. Different lowercase letters indicate statistically signiﬁcant differences based on an ANOVA followed by Duncan’s test (*P* < 0.05).

**
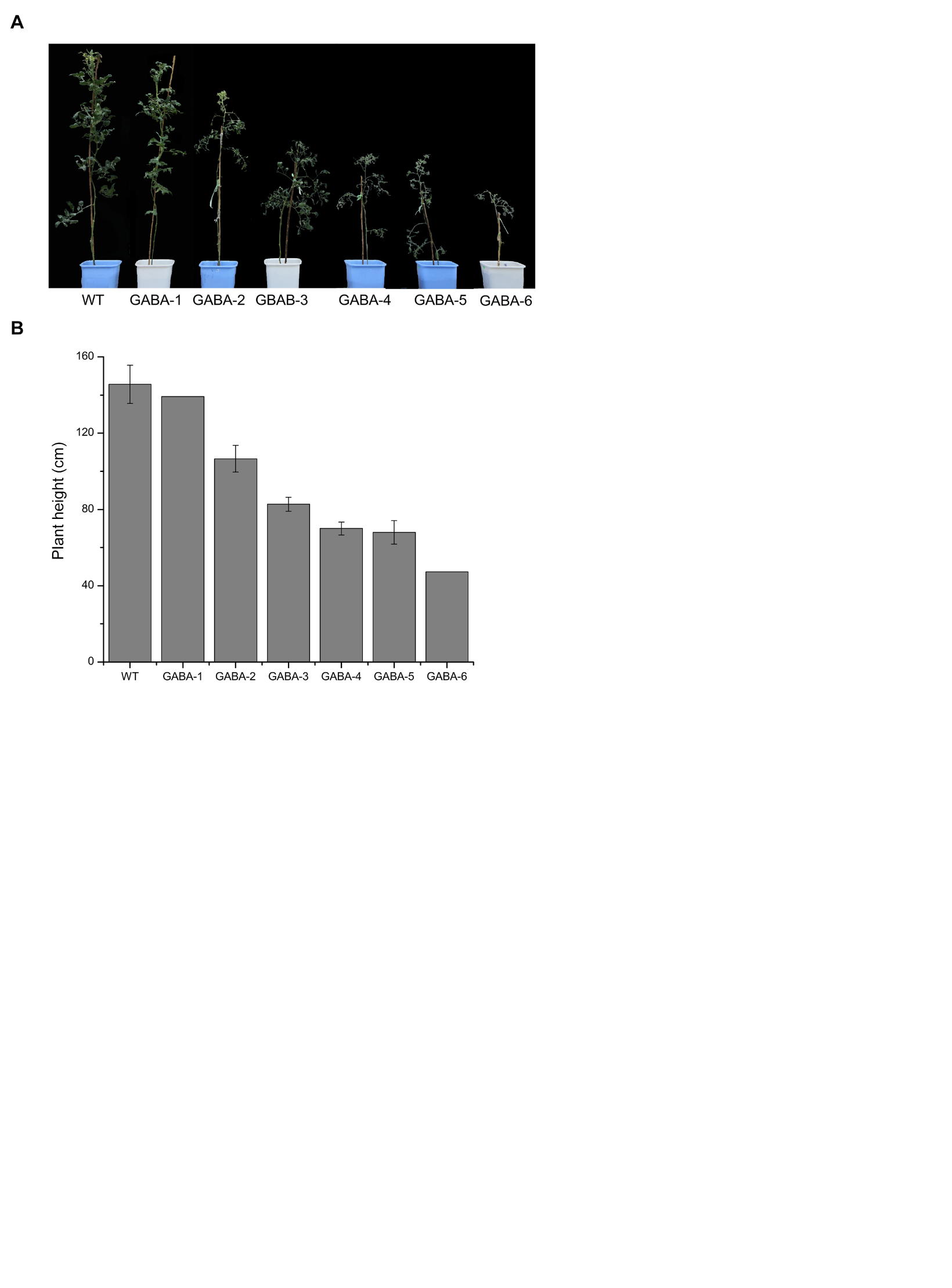
**

**Figure S6. Excessive GABA drafted tomato plants.**

(A) Photo of 60-day-old WT plant and GABA mutants. For each GABA mutant and WT plant, one representative line was selected and photographed. (B) Plant height of WT and GABA mutants. For WT, GABA-1, GABA-2, GABA-3, GABA-4, GABA-5, and GABA-6, 10, 1, 10, 10, 5, 7, and 1 plants were measured respectively. Error bars representing standard deviation.

**
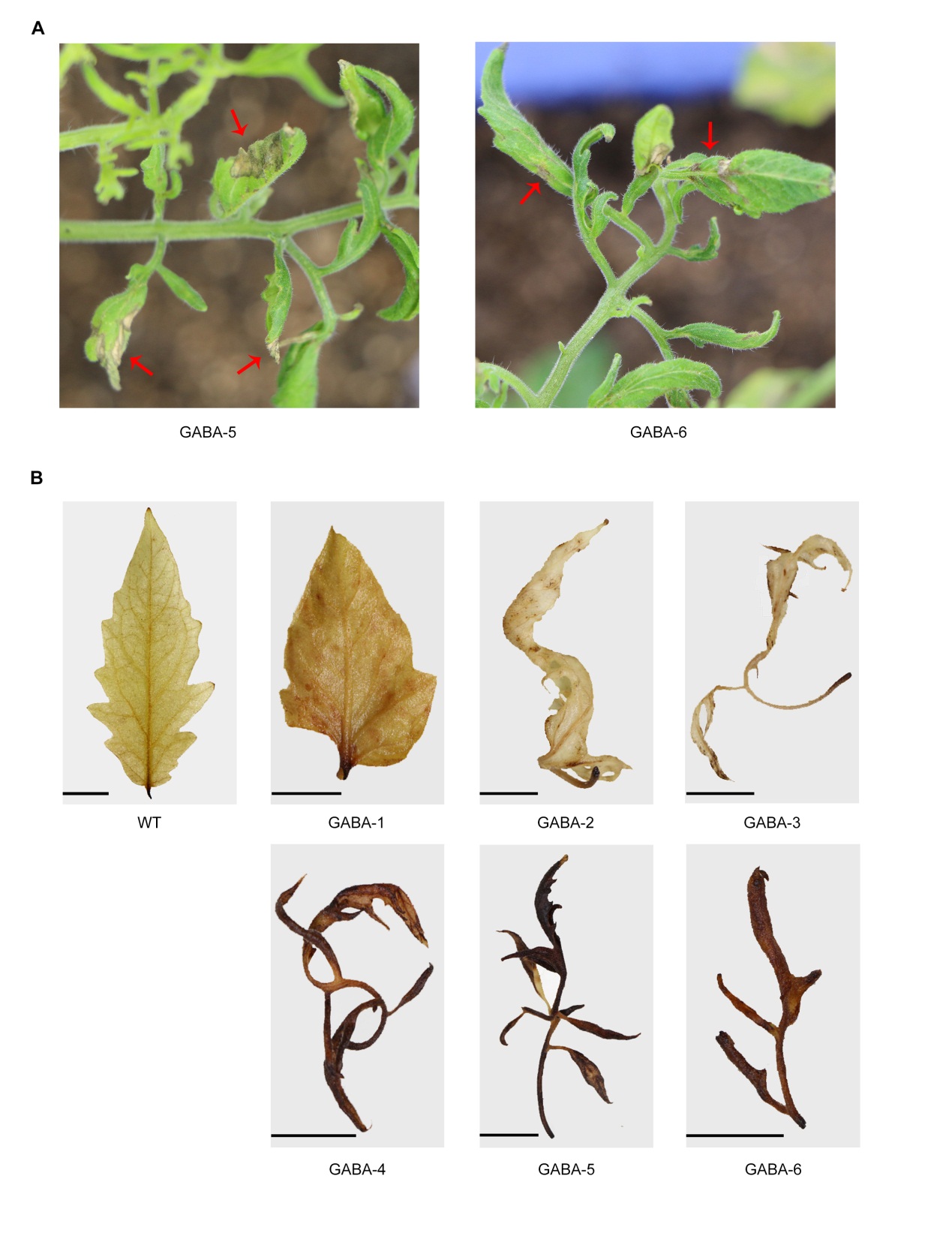
**

**Figure S7. Tissue necrosis of GABA mutants.**

(A) Image of leaves of GABA5 and GABA6 mutants. (B) Different GABA mutants show different H_2_O_2_ accumulation compared with WT. All leaves depicted are leaf no. 6. Scale bar=1cm.


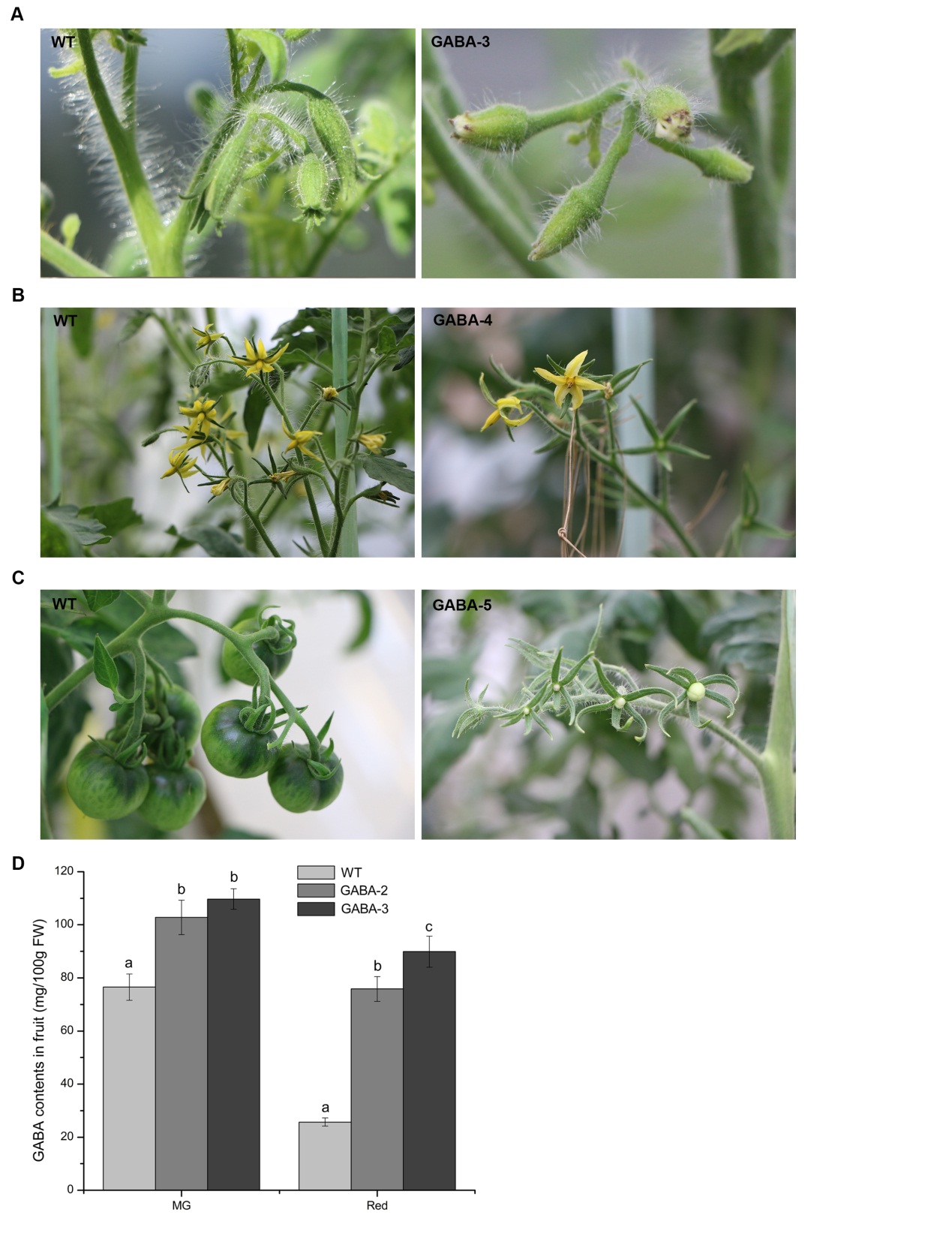


**Figure S8. Over accumulation of GABA causes smaller fruits due to impaired fertilization.**

(A) Buds formation and development were severely inhibited in GABA mutants compared with WT. (B) Few flowers in GABA mutants compared with WT. (C) Teratogeny-inactivated fruits in GABA mutants compared with WT.

**Table S1 Determination of *PDS* mutations that occurred in T0 transgenic AC plants**

| Target gene | No. of examined plants | No. of mutated plants | Mutation rate (%) | Homozygous | | Heterozygous | | Biallelic | |
| --- | --- | --- | --- | --- | --- | --- | --- | --- | --- |
|  |  |  |  | No. | % | No. | % | No. | % |
| *slyPDS* | 34 | 21 | 61.8 | 1 | 4.8 | 12 | 57.1 | 8 | 38.1 |
| Empty vector | 34 | 0 | 0 | 0 | | 0 | | 0 | |

**Table S2 Determination of *PDS* mutations that occurred in T0 transgenic MT plants**

| Target gene | No. of examined plants | No. of mutated plants | Mutation rate (%) | Homozygous | | Heterozygous | | Biallelic | |
| --- | --- | --- | --- | --- | --- | --- | --- | --- | --- |
|  |  |  |  | No. | % | No. | % | No. | % |
| *slyPDS* | 38 | 26 | 68.4 | 2 | 7.7 | 17 | 65.4 | 7 | 26.9 |
| Empty vector | 38 | 0 | 0 | 0 | | 0 | | 0 | |

**Table S3 Off-target detection in three CR-slyPDS mutants**

| Target | Name of putative off-target site | Putative  off-target locus | Sequence of the putative  off-target site | No. of  plants  sequenced | No. of  plants with mutations |
| --- | --- | --- | --- | --- | --- |
| *SlyPDS* (T1) | OFF1 | SL2.50ch04:39416782..39416804 | AGACTCCTACCAGCAATGCACGG | 6 | 0 |
|  | OFF2 | SL2.50ch07:25364070..25364092 | GGACTCTTTTAAGCAAAGCTTGG | 6 | 0 |
|  | OFF3 | SL2.50ch03:13186964..13186986 | CCCAACATTGCTTGCAAGTGTCC | 6 | 0 |
|  | OFF4 | SL2.50ch10:63927875..63927897 | TCAAGCAATGCTGGAAAGATACC | 6 | 0 |
| *SlyPDS* (T2) | OFF1 | SL2.50ch05:26011906..26011928 | GGCAGATGGGGTGACAGATGAGG | 6 | 0 |
|  | OFF2 | SL2.50ch06:45872085..45872107 | GCCACACACGGTGACAGAGGAAG | 5 | 0 |
|  | OFF3 | SL2.50ch12:25286496..25286518 | CCTCACCTCTCACCCTGTGTTGC | 6 | 0 |
|  | OFF4 | SL2.50ch01:68575795..68575817 | CCCCATCTCTCAGCCTGTTCTGC | 6 | 0 |

a, mismatching bases are marked in red.

**Table S4 Detection of mutations on off-target sites in CR-GABA mutants**

| Target | Name of putative off-target site | Putative  off-target locus | Sequence of the putative  off-target site | No. of  plants  sequenced | No. of  plants with mutations |
| --- | --- | --- | --- | --- | --- |
| *SlGABA-TP1* (T1) | OFF1 | SL2.50Ch1:18013218..18013240 | CCAGTACAGGCTCGGTCCCCTCT | 5 | 0 |
|  | OFF2 | SL2.50Ch9:8317731..8317753 | AGGGTGGACCCAGCCTCTCCTAG | 5 | 0 |
| *SlGABA-TP1* (T2) | OFF1 | SL2.50Ch1:88156177..88156199 | TCTTCCTCTAGCATGCGCAGTAG | 5 | 0 |
|  | OFF2 | SL2.50Ch1:86242265..86242287 | TCACTGCTGATGCAACAGAAAGC | 5 | 0 |
| *SlGABA-TP3*(T4) | OFF1 | SL2.50Ch12:44136954..44136976 | GGAATTCCATCAGGCATTGAAGG | 5 | 0 |
|  | OFF2 | SL2.50Ch1:65112126..65112148 | CAAATGTCATCCGACAGTGATGG | 5 | 0 |
| *SlCAT9* (T5) | OFF1 | SL2.50Ch5:17238174..17238196 | TGTGCAAATCCTAGGCAGAATAG | 5 | 0 |
|  | OFF2 | SL2.50Ch2:20580590..20580612 | GCTTTCTGCCTTAGGTTTGCTTC | 5 | 0 |
| *SlSSADH* (T6) | OFF1 | SL2.50Ch2:43257985..43258007 | CCTAATGCCTGCAAGCCCACAGG | 5 | 0 |
|  | OFF2 | SL2.50Ch2:37778181..37778203 | TCTTTGGGCTTGCAAGAACTCCG | 5 | 0 |

a, mismatching bases are marked in red.

**Table S5 Sequence of target sites**

| Gene | sequence (5’-3’) |
| --- | --- |
| *SlyPDS* | T1：GGACTCTTGCCAGCAATGCT |
|  | T2：GCCGGACAGGGTGACAGATG |
| *GABA-TP1* | T1：AGGGGGGAACGAGCCTCGCC |
|  | T2：GCATCCTGTTGCATGCGCAG |
| *GABA-TP2* | T3：ATCATCCATGCCGCAGTAAA |
| *GABA-TP3* | T4：AGAATGCCATCCGGCAGTGA |
| *SlyCAT9* | T5：GATGCAAACCGTAGGCAGAA |
| *SlySSADH* | T6：CGCACTGCTTGCAAGCCCAC |

**Table S6 Primers used for recombinant pYLCRISPR/Cas9 vector construction**

| PCR | Primer | Sequence (5’-3’) |
| --- | --- | --- |
| 1^st^ PCR | U-F | CTCCGTTTTACCTGTGGAATCG |
|  | gR-R | CGGAGGAAAATTCCATCCAC |
|  | AtU3d-slyPDS T1 Rev | AGCATTGCTGGCAAGAGTCCTGACCAATGGTGCTTTG |
|  | gR-slyPDS T1 For | GGACTCTTGCCAGCAATGCTGTTTTAGAGCTAGAAAT |
|  | AtU3b-slyPDS T2 Rev | CATCTGTCACCCTGTCCGGCTGACCAATGTTGCTCC |
|  | gR-slyPDS T2 For | GCCGGACAGGGTGACAGATGGTTTTAGAGCTAGAAAT |
|  | AtU3d-TP1 T1 Rev | GGCGAGGCTCGTTCCCCCCTgaccaatggtgctttg |
|  | gR-TP1 T1 For | GGGGGGAACGAGCCTCGCCgttttagagctagaaat |
|  | AtU3d-TP1T2 Rev | CTGCGCATGCAACAGGATGCTgaccaatggtgctttg |
|  | gR-TP1 T2 For | GCATCCTGTTGCATGCGCAGgttttagagctagaaat |
|  | AtU3b-TP2 T3 Rev | TTTACTGCGGCATGGATGATgaccaatgttgctcc |
|  | gR-TP2 T3 For | TCATCCATGCCGCAGTAAAgttttagagctagaaat |
|  | AtU3b-TP3 T4 Rev | TCACTGCCGGATGGCATTCTgaccaatgttgctcc |
|  | gR-TP3 T4 For | GAATGCCATCCGGCAGTGAgttttagagctagaaat |
|  | AtU6-1 slyCAT9 T5 Rev | TTCTGCCTACGGTTTGCATCaatcactacttcgtct |
|  | gR slyCAT9 T5 For | ATGCAAACCGTAGGCAGAAgttttagagctagaaat |
|  | AtU6-29 slySSADH T6 Rev | GTGGGCTTGCAAGCAGTGCGCaatctcttagtcgact |
|  | gR-slySSADH T6 For | CGCACTGCTTGCAAGCCCACgttttagagctagaaat |
| 2^nd^ PCR | Pps-GGL | TTCAGAggtctcTctcgACTAGTATGGAATCGGCAGCAAAGG |
|  | Pgs-GG2 | AGCGTGggtctcGtcagggTCCATCCACTCCAAGCTC |
|  | Pps-GG2 | TTCAGAggtctcTctgacacTGGAATCGGCAGCAAAGG |
|  | Pgs-GG3 | AGCGTGggtctcGtcttcacTCCATCCACTCCAAGCTC |
|  | Pps-GG3 | TTCAGAggtctcTaagacttTGGAATCGGCAGCAAAGG |
|  | Pgs-GG4 | AGCGTGggtctcGagtccttTCCATCCACTCCAAGCTC |
|  | Pps-GG4 | TTCAGAggtctcTgactacaTGGAATCGGCAGCAAAGG |
|  | Pgs-GG5 | AGCGTGggtctcGgtccacaTCCATCCACTCCAAGCTC |
|  | Pps-GG5 | TTCAGAggtctcTggacttgTGGAATCGGCAGCAAAGG |
|  | Pgs-GG6 | AGCGTGggtctcGcagatagTCCATCCACTCCAAGCTC |
|  | Pps-GG6 | TTCAGAggtctcTtctgcaaTGGAATCGGCAGCAAAGG |
|  | Pgs-GGR | AGCGTGggtctcGaccgACGCGTATCCATCCACTCCAAGCT |

**Table S7 Primers used for target site mutation analysis**

| Target site | Primer | Sequence (5’-3’) |
| --- | --- | --- |
| slyPDS-T1/T2 | For | ACACCTATGGGACTTTACTAACCT |
|  | Rev | CTGAAGAAACCTGTTCAATGCGA |
|  | Seq | CCTATGGGACTTTACTAACC |
| T1 (slyGABA-TP1) | T_1_For | TCAATGTCTGCTATGTTATCCTG |
|  | T_1_Rev | CTATCCCAAAAACGATGTTCTTC |
|  | T_1_seq | AAAACCTGGGTATCATTGGCTT |
| T2 (slyGABA-TP1) | T_2_For | TTTGGGAGGCTTGGAACA |
|  | T_2_Rev | CTGAGGCGGCAATGAGAC |
|  | T_2_seq | TCAAACCAAGTCCCCTAATC |
| T3 (slyGABA-TP2) | T_3_For | GCCAAGACTAATGGGTAAAG |
|  | T_3_Rev | CGTCATAGACATAAGAACCCT |
|  | T_3_seq | CGAAACTCAGCAAATCACCTTA |
| T4 (slyGABA-TP3) | T_4_For | ATACAGGTCAGATGGAGTAGCAC |
|  | T_4_Rev | ACGAGGCTCATTTCCACC |
|  | T_4_seq | ACATCATAGACATACGAACCC |
| T5 (slyCAT9) | T_5_For | CATCCACTTTGCCATCTTGAA |
|  | T_5_Rev | ATCCCAACTGTAACAATGCTG |
|  | T_5_seq | CATCATTGGGGAACGAAGT |
| T6 (slySSADH) | T_6_For | GGGAGGTAATAACAGATGTGCCA |
|  | T_6_Rev | TGCTGCTTCATTGATTAGAGGG |
|  | T_6_seq | GCCCTGCTCTTGCTTGTGGTT |

**Table S8 Primers used for off-target site mutation analysis**

| Off-target site | Primer | Sequence(5’-3’) |
| --- | --- | --- |
| PDS-T1 off-target site 1 | For | ATAATGGTCTAAAAGCACGCAG |
|  | Rev | CATTTTCTCCCTCTTCATCTCC |
|  | Seq | CACGCAGAATTGTTAGGATA |
| PDS-T1 off-target site 2 | For | GTATGGTCTATGTCGGTTATTGC |
|  | Rev | AAGAATACTTGGGACACGGC |
|  | Seq | TCAAGTGAGCCTTAGAATCA |
| PDS-T1 off-target site 3 | For | TCCCTCCAAAGCAAAGTGAC |
|  | Rev | TTGTGTCTGAGAAGGGTGCT |
|  | Seq | TTGGGCTCTTTTAGGAGGAG |
| PDS-T1 off-target site 4 | For | GAGAAGAGTGGCAATGTAGGAC |
|  | Rev | CAGACCCCTGCTTCTACAACTC |
|  | Seq | AAGTATTTTGGTCATGCCCC |
| PDS-T2 off-target site 1 | For | GTTGCTTATGGTCAGGGTTGGT |
|  | Rev | CTACATTCTCATCAGCATTCGT |
|  | Seq | AGGCATTGATAGCATGGGAG |
| PDS-T2 off-target site 2 | For | CTCACCGCTGGAACGGATACTA |
|  | Rev | CTAAATGGAGCATAGCCAAGCC |
|  | Seq | ACCTCGTTGCAATGGATTAT |
| PDS-T2 off-target site 3 | For | ATGACCAAGAAACGCCACCG |
|  | Rev | GTTTGTGAAGAAGAAGGATGGGT |
|  | Seq | TATACTTGGGAAATAGGTGG |
| PDS-T2 off-target site 4 | For | CAAGGGCTCGGGTCAAGTCA |
|  | Rev | TCCCTTCATCCTCCACGGTA |
|  | Seq | CATCATTGATAACTGCCTTG |
| GABA-TP1-T1 off-target site 1 | For | GTATCCATAACCCATTCCGTG |
|  | Rev | CAAGGGTAAGGCTACAGAGAA |
|  | Seq | GAGCATCCTCAAACGAGTAAGTA |
| GABA-TP1-T1 off-target site 2 | For | ATGATACTACCCTTAGCCTGGA |
|  | Rev | TGTTTGATGAGTTCTACACGGA |
|  | Seq | CCCTAAAAACTGCTACGCTAAA |
| GABA-TP1-T2 off-target site 1 | For | GATGATGGTATGTCTCCTCTGC |
|  | Rev | CATAACATTGACATTAGCCCC |
|  | Seq | AGTCTGCTCTTTCGTTATGCT |
| GABA-TP1-T2 off-target site 2 | For | GCTACTGTGACGAAGACGGAA |
|  | Rev | TCATACCTCAAATGTTCTCCCT |
|  | Seq | CAGATGAAAACAACCGTTAGCA |
| GABA-TP3-T4 off-target site 1 | For | ATCCACTTTGTCCCAATCCTA |
|  | Rev | AAAAAGGCGATAAGACACACC |
|  | Seq | AGATGATGCTCTAATCCATAACC |
| GABA-TP3-T4 off-target site 2 | For | AACAAAGTTGATTGGTGGGTAG |
|  | Rev | GGTAAGTCCTCATAGAATCGTGG |
|  | Seq | CCGCCATAACAAACAAAACTAC |
| CAT9-T5 off-target site 1 | For | TGACCCATTTAGTCGTCTTGAG |
|  | Rev | GCTATCCTCTTCGTTTCTCCCT |
|  | Seq | TGGCGATAATACCCCACTAAT |
| CAT9-T5 off-target site 2 | For | TCCTAATGTGGAGTTGACGAC |
|  | Rev | GGCTTACTTTTGGCAGGACTA |
|  | Seq | TTATTGAAGTCTCGGGTGTCTG |
| SSADH-T6 off-target site 1 | For | AAATGGAAGATAAAGCGAGTG |
|  | Rev | CGAGAGAGAAAGGGTGAGACA |
|  | Seq | TTCCTGGTCCATCTCTTCGTA |
| SSADH-T6 off-target site 2 | For | CACATTGATGGGACTCTCTTAGC |
|  | Rev | GACAACTTTGATGATTAGGGCA |
|  | Seq | CGTTATGACATCCGTCTACCA |

**Table S9 Primers used for qRT-PCR**

| Gene | Primer | Sequence (5’-3’) |
| --- | --- | --- |
| *SlGABA-TP1* | For | GCAGTTAGCAGTTTACAGGAG |
|  | Rev | GACCCTTGAACCCTTTATTATCAG |
| *SlGABA-TP2* | For | TGCCCTCATTATTGGAACTATCAC |
|  | Rev | AAGCAGCAACTGTTTCAGGAC |
| *SlGABA-TP3* | For | CTACCAACCAGGTTGGTCCT |
|  | Rev | ATCATAGACATACGAACCCTCG |
| *SlSSADH* | For | GGGAGGTAATAACAGATGTGCCA |
|  | Rev | TCCTCAGCGGAGAACTCAATA |
| *SlGAD1* | For | GATTTCCGTTTGCCTCTTGTG |
|  | Rev | CGTCCTTATAACCCTCAAAGCC |
| *SlGAD2* | For | AAATGCAAGGGTACTAAGAGAAGG |
|  | Rev | GATGTCCATAACCAGTCGCTC |
| *SlGAD3* | For | TAAACAAACTGGATGGGACAC |
|  | Rev | CCAATACCAGCATAAACAAGCC |

**Table S10 Elution program of HPLC-MS**

| 20mM NH_4_Ac (pH 5.0) | ACN:H_2_O=80:20 | Time (min) |
| --- | --- | --- |
| 97% | 3% | 0 |
| 88% | 12% | 2 |
| 66% | 34% | 9.5 |
| 0% | 100% | 10 |
| 0% | 100% | 12 |
| 97% | 3% | 12.5 |
| 97% | 3% | 15.5 |
